# The Economics and Econometrics of Gene–Environment Interplay

**DOI:** 10.1093/restud/rdaf034

**Published:** 2025-06-11

**Authors:** Pietro Biroli, Titus Galama, Stephanie von Hinke, Hans van Kippersluis, Cornelius A. Rietveld, Kevin Thom

**Affiliations:** University of Bologna, Italy; University of Southern California, USA; VU University Amsterdam, The Netherlands; University of Bristol, UK; Institute for Fiscal Studies, UK; Erasmus University Rotterdam, The Netherlands; Erasmus University Rotterdam, The Netherlands; University of Wisconsin, USA

**Keywords:** ALSPAC, Gene–environment interplay, Genoeconomics, Polygenic indices, School entry policies, Social science genetics, D1, D3, I1, I2, J1

## Abstract

We discuss how to estimate the interplay between genes (nature) and environments (nurture), with an empirical illustration of the moderating effect of school starting age on one’s genetic predisposition towards educational attainment. We argue that gene–environment (G×E) studies can be instrumental for (i) assessing treatment effect heterogeneity, (ii) testing theoretical predictions, and (iii) uncovering mechanisms, thereby improving understanding of how (policy) interventions affect population subgroups. Empirically, we find that being old-for-grade and having a higher genetic propensity for education benefits children on assessment tests as they progress through school. In this setting, families appear to increase genetic inequalities while schools seem to reduce them.

## 1. INTRODUCTION

The debate over the relative importance of nature versus nurture in the development of human traits is amongst the oldest in the social sciences. Decades of twin studies have demonstrated that genetic factors account for approximately 25–75% of the between-individual variation in a wide range of behaviours, traits, and outcomes (Polderman et al., 2015). This finding is summarized in the *first law of behavioural genetics*: “all human behavioural traits are heritable” (Turkheimer, 2000). Of course, environmental factors are similarly important. Moreover, genes and environments do not operate in isolation. Gene-by-environment (G×E) interaction occurs when environmental factors influence the relationship between the genotype and the outcome of interest, or vice versa.^1^ Most outcomes arise from a web of both genetic and environmental influences that may interact in complex ways (Hunter, 2005; Heckman, 2007).

Rapid reductions in the cost of collecting and processing genetic material have expanded the availability of genetic data, making it feasible to study complex G×E interplay in econometric analyses. Single-nucleotide polymorphisms or SNPs (pronounced “snips”) represent the most common type of genetic variation, and occur when individuals differ in the molecules they possess at specific positions on the genome. With the ability to measure SNP-level variation, researchers can study the influence of a single SNP, or the combined influence of millions of SNPs aggregated into a polygenic index (PGI).^2^ PGIs have substantially greater predictive power than do single SNPs, and are now available in many rich (longitudinal) datasets. Since anyone can now explore the role of G×E in shaping individual outcomes, even without much knowledge of genetics, economists need to be aware of the complexities of genetic data and the interpretation of G×E results. We aim to provide this essential guidance here.

We build on previous surveys of the use of genetic data in economic analyses,^3^ and focus on the economics and econometrics of G×E interplay. Our contribution is two fold. In the first part of the paper, we introduce key concepts, discuss recent developments in the field, and highlight the intricacies of understanding and interpreting G×E estimates. In the second part, we offer practical guidance on how to use genetic data to explore nature–nurture interplay. We do this by testing for the presence of G×E interactions in the context of school entry policies in the U.K. Specifically, we examine interactions between the age of school entry and a PGI for educational attainment (EA) in shaping performance on standardized tests throughout childhood, from ages 4 to 16.

There is a long-standing debate (Heath et al., 1985) on whether educational institutions moderate inequalities induced by the “genetic lottery” (Harden, 2021a). Policies related to entry into the formal schooling system—including the age of school entry—are of particular interest since they determine the moment at which the bulk of skill development is transferred from parents to schools. With current, state-of-the-art EA PGIs explaining 12–16% of the variation in EA, on par with some of the strongest environmental determinants such as parental education and income (Lee et al., 2018; Okbay et al., 2022), molecular genetic data can now be used to shed light on its role in shaping inequalities.

In this paper, we study whether and how school starting age moderates the relationship between genotype and academic achievement, and quantify the magnitude of such G×E effects. In the U.K., strict birth-date cut-offs dictate that individuals born just before September 1 start formal schooling at age 4 (making them relatively young in their class), while those born on September 1 or just after start around age 5 (making them relatively old in their class). This quasi-randomly assigned treatment is multifaceted, as the children born just after September 1 (i) enter school and take tests at an older age, (ii) are relatively older than their classmates, and (iii) receive an extra year of exposure to their parental rearing environments before starting formal schooling. The existing literature—discussed more extensively below—has found that children who are older when they start schooling (and therefore also older relative to their peers) perform substantially better on grade-level tests.

We estimate the treatment effect—*i.e.* being old-for-grade—on a series of five standardized tests. The first test is taken upon entering primary school, *i.e.* before any exposure to formal schooling. For this first test, we find a strong positive interaction between the genetic propensity for EA (as measured by the EA PGI) and being old-for-grade. That is, old-for-grade children perform better on the Entry Assessment test, and high EA PGI children gain more, on average, from entering school later. Since these tests are taken before children enter school, the treatment reflects biological maturation and having spent more time at home (*e.g.* more parental investments). Interestingly, for standardized tests taken several years after the start of formal schooling, we generally find a pattern of negative interactions between assignment to old-for-grade and the EA PGI. That is, children with lower EA PGI values gain more from being old-for-grade in the formal schooling system. In addition to reflecting biological maturation and more time at home, the treatment now also includes effects that operate through the school environment, suggesting that formal schooling reduces genetic inequality for EA. In Section 4.1, we discuss how a simple economic model can help with the interpretation of these opposing G×E estimates.

The study of G×E interplay may advance basic science by improving our understanding of the role of nature and nurture in shaping human capabilities. Beyond this, we contend that studying G×E interplay is of general interest, even for those with no inherent interest in the biological origins of economic outcomes, for at least three reasons:

### (1) Treatment effect heterogeneity and inequality:

Researchers and policy-makers are often interested in characterizing the distribution of treatment effects associated with a particular intervention or policy change. Their impact depends on which individuals are most affected, and how they respond. G×E studies can reveal whether and how individuals with different genetic endowments are differentially affected, extending our ability to describe treatment effect heterogeneity beyond standard observables. A review of recent papers in this literature, with a focus on EA, suggests that G×E interactions are quantitatively important and around 1.5–3 times smaller than the main effects (see Supplementary Appendix D). In our empirical analysis, we find that old-for-grade children score 0.38 standard deviations higher on the standardized test taken at ages 10–11, and this effect is 25% higher for children with a PGI one standard deviation below the mean.

Knowing whether a policy moderates differences arising from genes is of interest because people exhibit very different moral intuitions for inequalities arising from genetics or from early life socioeconomic conditions (*e.g.* Sandel, 2020; Harden, 2021a). Some consider genetic gradients a signal of meritocracy (*e.g.* Rimfeld et al., 2018), whereas others view genetic differences as yet another layer of inequality of opportunity since genes—like rearing environments—are unchosen and unearned (*e.g.* Kweon et al., 2020; Harden, 2021a). Understanding treatment effect heterogeneity arising from genes also holds a special significance for studying intergenerational mobility: since genes are inherited, policies that benefit one generation may be propagated to the next and thereby affect long-run inequalities.

Results from G×E studies may also enable the targeting of scarce resources, or the development of personalized interventions. In medicine, genetic data are already widely used for diagnostic purposes and for the tailoring of treatments. G×E effects for complex economic outcomes like EA raise the possibility of similar applications in skill development. In the short run, it seems unlikely that school systems or other institutions would incur the substantial costs of creating personalized experiences informed by genetics. It is also debatable whether the use of genetic data will ever be informative and beneficial enough to justify its institutional use given the large ethical and privacy concerns (Meyer et al., 2023). However, our empirical application highlights that G×E research might be more immediately useful at the level of household decision-making. Well-identified G×E studies are a prerequisite for assessing the viability of such applications. Even here, enthusiasm over such applications may be premature since the current predictive power of PGIs may be too low for useful *individual*-level recommendations (Morris et al., 2020b; Turley et al., 2021). Still, depending on preferences and constraints, households on the margin could conceivably use genetic information as one input among many when deciding the best environment for their child. For example, our empirical results suggest that genetic information may help parents on the margin determine an appropriate age at entry into formal schooling within educational systems that allow such choice.

### (2) Testing theoretical predictions:

Economic theory predicts that idiosyncratic characteristics like preferences, health endowments, and abilities shape individual choices and generate differences in how individuals respond to a common environmental change. For example, models of skill formation often assume that parents actively respond to the endowments of their children (*e.g.* Becker and Tomes, 1976; Behrman, 1997; Currie and Almond, 2011), and that abilities and endowments are productive complements with parental investments (*e.g.* Ben-Porath, 1967; Becker and Tomes, 1986; Cunha and Heckman, 2007). Since primitive variables, such as endowments and abilities, are typically hard to measure, testing such predictions can be challenging. Observable genetic variation—especially random variation within families—offers a new and powerful way to measure such characteristics, and test theoretical predictions. For example, Breinholt and Conley (2023), Fletcher et al. (2023), Sanz-de-Galdeano and Terskaya (2023), and Houmark et al. (2024) provide evidence that parental investments indeed respond to children’s genotypes. The presence (or absence) of G×E interactions can similarly test assumptions or theoretical predictions. For example, Muslimova et al. (2024) find that children with higher EA PGI values relative to their siblings gain more from being the firstborn. Since parental investments are a dominant channel driving birth order effects, these results are consistent with complementarity between genetic endowments and parental inputs in the formation of human capital.

In our application, G×E results for age at entry have implications for the role of formal schooling and patterns of substitutability in the human capital production function. Although high PGI students benefit the most from being older in terms of entry skills *prior to the start of formal schooling*, low PGI students benefit the most in *in-school* standardized testing. This combination of results suggests that being old-for-grade during the formal schooling years brings about a series of environments or investments that together act as technical substitutes for genetic endowments. These results place limits on how strongly past skills, genetic endowments, and being old-for-grade complement one another in the context of the formal schooling system.

### (3) Uncovering mechanisms:

Evidence on G×E interplay can also provide clues about the economic or behavioural mechanisms through which genetic factors operate. For example, Barth et al. (2020) find that access to defined benefit pension plans substantially moderate the relationship between the EA PGI and household wealth. This suggests that genetic endowments related to schooling influence household wealth not only through earnings and consumptionsavings choices, but also through financial decision-making and portfolio choice. Researchers developing and estimating quantitative life-cycle models of these outcomes have to choose how to incorporate heterogeneity (*e.g.* which primitive parameters to make random, and what kinds of covariance structures to allow). Since genetic factors may constitute a substantial portion of “unobserved heterogeneity”, evidence on the mechanisms through which they operate may help guide modelling choices (Benjamin et al., 2012b). For example, the results from Barth et al. (2020) suggest that in life-cycle models of education and inequality, it may be important to allow sources of heterogeneity that shift education choices to be correlated with heterogeneity in primitive parameters relative to financial decision-making or rates of return on invested wealth.

Finally, just as the empirical analysis of G×E can provide novel insights for economists, there is great potential to using the toolbox of economics to better design G×E studies and thus advance the field of genetics in general and social-science genetics in particular. Since G×E interplay often stems from endogenous behavioural adjustments, economic theory can help clarify why and when such interplay might occur and what it implies for policy. Empirically, both genetic endowments and environmental factors are typically endogenous in the study of a particular outcome. Ongoing advances in methods and data are creating opportunities for the causal inference of genetic factors. Economists have substantial experience with exploiting exogenous variation in environmental exposures and have developed a large toolbox to deal with endogeneity. Given the importance of establishing which causal environmental exposures moderate what genetic predispositions, economists are well-positioned to improve our understanding of the complex interplay between nature and nurture in shaping life outcomes.

This paper is organized as follows. Section 2 discusses the measurement of G, distinctions between environments that act as moderators or mediators of genetic effects, the interpretation of genetic effects, and how the natural experiment of genetic inheritance can be exploited to analyse causal genetic effects. In Section 3, we discuss the intricacies of interpreting an empirical G×E model, providing a systematic categorization of G×E analyses and a discussion of the direction and nature of the bias in G, E, and G×E for each case relative to the ideal (unbiased) case, in which both G and E are exogenous. Readers familiar with the (social science) genetics literature may wish to skip directly to Section 4, where we uncover a novel G×E interaction between being old-for-grade in school (E; exogenous due to sharp cut-offs in month of birth (MoB) determining eligibility for school entry) and the genetic propensity for EA (G; exogenous since the child’s genotype is random conditional on the parental genotypes) on test scores at different ages throughout childhood. Section 5 concludes by providing a brief discussion of our results.

## 2. MEASURING AND INTERPRETING G

The following discussion assumes a minimum level of understanding of genetic concepts, with the Glossary in Supplementary Appendix A providing definitions of the genetic terminology used in the paper. We suggest that those new to genetics start with Supplementary Appendix B for a short primer.

More than 15 years of genome-wide association studies (GWAS) show that virtually all outcomes that social science researchers are interested in are highly “polygenic” (Visscher et al., 2008; Loos, 2020; Abdellaoui et al., 2023). That is, there is no “gene for” a certain outcome, but individuals rather fall somewhere on a scale of the genetic predisposition to a certain outcome that reflects the aggregation of numerous small contributions of millions of genetic variants (SNPs). Most studies, therefore, tend to use PGIs (Becker et al., 2021) instead of individual SNPs, where a PGI is a weighted sum of individual SNPs.

While a PGI constitutes the best linear genetic predictor of an outcome (Mills et al., 2020; Becker et al., 2021), it is important to emphasize that this holds within the environmental and demographic context of the discovery sample (Domingue et al., 2020; Mills et al., 2020). Most existing PGIs are derived from discovery samples based on European-ancestry populations, with limited “portability” to populations with different ancestries (Benjamin et al., 2024). Thus, the association between a PGI and an outcome cannot be interpreted as an immutable biological relationship (*e.g.* Kweon et al., 2020; Mostafavi et al., 2020): the effects depend on the context, *i.e.* on the environment. For example, even though alcohol metabolism and dependence are partially determined by genetic factors, in an environment where alcohol consumption is illegal or low because of cultural reasons (Cho et al., 2015), the genetic effects would be (close to) zero.

Environmental factors may influence the relationship between the PGI and the outcome of interest through at least three channels: as *moderators, confounders*, and *mediators*. As a *moderator*, the environment could change the strength of the relationship between a PGI and the outcome. This is precisely the topic of this paper, where environmental moderation would be reflected by the G×E interaction term.

**Figure 1:**
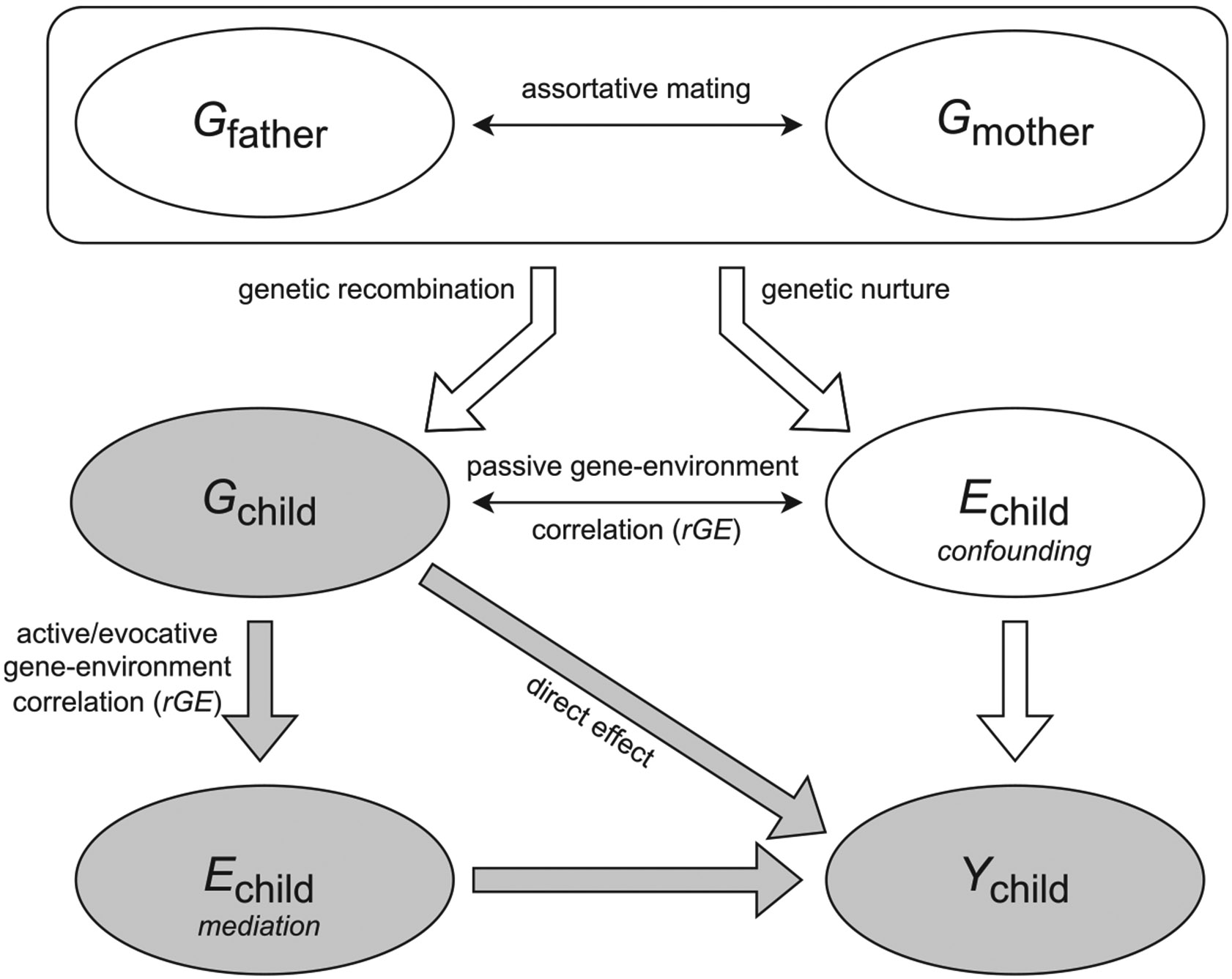
The relationships between parental genes ($G_{\text{father}}$ and $G_{\text{mother}}$), the child’s genes ($G_{\text{child}}$), environmental factors ( $E_{\text{child}}$), and the outcome ($Y_{\text{child}}$). The grey area represents the “causal” region of the diagram (explained further in the text)

To understand the environment as a *confounder* and *mediator* of the PGI effect, we consider EA as the outcome variable. Figure 1 shows a schematic of the relationships between the parental genotypes ($G_{\text{father}}$ and $G_{\text{mother}}$), the environment ($E_{\text{child}}$), the child’s genotype ($G_{\text{child}}$), and the child’s outcome ($Y_{\text{chil}}$). The top part of the diagram shows how the genotypes of mothers and fathers may be correlated through assortative mating.^4^ The arrow from $G_{\text{father}}$ and $G_{\text{mother}}$ to $G_{\text{child}}$ (genetic recombination) reflects the notion that a child inherits her genotype from her parents through recombination of the parental genotypes. The child’s genotype, in turn, has a direct effect on the outcome (arrow from $G_{\text{child}}$ to $Y_{\text{child}}$, “direct effect”), while the parental genotypes may be associated with the environment (arrow from $G_{\text{mother}}$ and $G_{\text{father}}$ to $E_{\text{child}}$). That is, parents with genotypes conducive to education may provide an environment more beneficial to their child’s learning (so-called genetic nurture; Bates et al., 2018; Kong et al., 2018; Wertz et al., 2018), and/or genotypes of parents may simply be correlated with environmental advantages that originate from older ancestries (so-called dynastic effects, Nivard et al., 2024). This environment, in turn, may raise the child’s educational achievement (arrow from $E_{\text{child}}$ to $Y_{\text{child}}$). Here, the child’s environment acts as a *confounder* because $E_{\text{child}}$ not only influences the outcome $Y_{\text{child}}$ but is also correlated with the child’s genotype $G_{\text{child}}$ through the parental genotypes. Genetic nurture and dynastic effects are examples of a so-called “passive gene–environment correlation” (*rGE*) (double thin arrow between $G_{\text{child}}$ and $E_{\text{child}}$), which occurs when individuals’ genotypes relate to their environment, but that environment is not a consequence of the child’s genotype $G_{\text{child}}$.^5^

In general, gene–environment correlation (*rGE*) describes the phenomenon of certain environments being more prevalent among carriers of certain genotypes (Plomin et al., 1977; Fletcher and Conley, 2013). In addition to *passive rGE*, there are two other types of *rGE*: active and evocative *rGE* (Plomin et al., 1977). *Active* gene–environment correlation occurs when individuals with certain genotypes self-select into certain environments $E_{\text{child}}$ (“mediation”). For example, someone with a high genetic predisposition for education may find it easier to apply for and be accepted into a selective, high-quality university. *Evocative* gene–environment correlation occurs when someone’s genetic predisposition $G_{\text{child}}$ invokes a certain environmental response; $E_{\text{child}}$ (“mediation”). For example, a child with a high genetic predisposition for calmness may be treated differently by her parents and teachers, creating an environment that may be more conducive to learning. Hence, both active and evocative *rGE* imply that the environment $E_{\text{child}}$ is a *consequence* of the child’s genotype $G_{\text{child}}$. These environments, in turn, may influence the child’s EA $Y_{\text{child}}$. Hence, through active and evocative *rGE*, the environment may act as a *mediator*, a variable that is influenced by the child’s genotype $G_{\text{child}}$ and that in turn influences the outcome $Y_{\text{child}}$.

Genotypes have the useful property of being fixed at conception. Thus, the outcome cannot affect the genotype (*i.e.* there is no reverse causality). We adopt the view that the causal effect of genotype can be thought of as a *variant substitution* effect (Lee and Chow, 2013; Morris et al., 2020a). That is, the causal effect of a genetic variant is the counterfactual change in an individual’s outcome that would occur had that genetic variant been different at conception, with all else held constant.^6^ In the diagram, the mechanisms through which the child’s genotype $G_{\text{child}}$ operate can be through direct pathways (*e.g.* gene expression) but can also be environmentally driven (*e.g.* through active or evocative *rGE*). Both direct and indirect genetic pathways fall within the causal part of the diagram (shown in grey).

The existence of (passive) *rGE* implies that the offspring’s genotype $G_{\text{child}}$ and outcome $Y_{\text{child}}$ are simultaneously influenced by the parental genotypes. This implies that the offspring genotype $G_{\text{child}}$ is endogenous. As we discuss in Section 3.1, controlling for the parental genotype can fully address confounding due to passive *rGE*, allowing for causal inference. This is because, conditional on the parental genotypes, the child’s genotype is as good as random (“Mendel’s Law”), breaking the link between child genotype $G_{\text{child}}$ and the confounding child environment $E_{\text{child}}$.^7^ Controlling for parental genotypes can also solve biases arising from assortative mating, which typically inflates the association between $G_{\text{child}}$ and the outcome $Y_{\text{child}}$ (Young, 2023).^8^ Controlling for the parental genotypes, therefore, isolates a clearly defined causal effect, namely, the change in the expected value of the outcome variable that results from a hypothetical change of the allele count of a given SNP at conception (Benjamin et al., 2012a). These effects will be aggregated over all SNPs in a PGI and averaged over all individuals in the data.

In the next section, we discuss approaches to addressing the endogeneity of the PGI and the environment in analyses of G×E interplay and the more general question of how to estimate the *moderating* effect of an environment on a genotype.

## 3. EMPIRICAL APPROACHES TO ESTIMATING G×E INTERPLAY

The core idea behind gene–environment interplay (G×E) is that effects of nature and nurture are not additive and separable but intrinsically joined and non-linear. Interaction effects have also be referred to as synergies, complementarities, supermodularity, or heterogeneity of treatment effects (Mullahy, 1999). Consider a data-generating process in which the outcome $Y_{i}=F(a^{*},G_{i},E_{i},e_{i})$ is a function of genetic endowments $G_{i}$, the environment $E_{i}$, individual choices $a^{*}=a^{*}(G_{i},E_{i},e_{i})$, and random factors $e_{i}$. To test for the existence of G×E, one needs to test for non-linearities in the function F(⋅), specifically that $\partial^{2}F∕\partial G\partial E\neq 0$. After taking a second-order Taylor approximation, we focus on the identification of G×E in a typical linear regression model:

(1) $$Y_{i}=\beta_{0}+\beta_{G}G_{i}+\beta_{E}E_{i}+\beta_{G\times E}(G_{i}\times E_{i})+\beta_{G^{2}}G_{i}^{2}+\beta_{E^{2}}E_{i}^{2}\;\;+\beta_{1}X_{i}+\beta_{2}(G_{i}\times X_{i})+\beta_{3}(E_{i}\times X_{i})+\varepsilon_{i}.$$

where we include control variables $X_{i}$, which should not be caused by G and E to avoid bias due to “bad controls” (*e.g.* Angrist and Pischke, 2008). We also include a full set of interactions between the control variables and the genetic and environmental measures—($G_{i}\times X_{i}$) and ($E_{i}\times X_{i}$), respectively—to ensure that the coefficient of interest $\beta_{G\times E}$ does not capture spurious correlations between $X_{i}$ and either $G_{i}$ or $E_{i}$ (Keller, 2014; Feigenberg et al., 2023).

In Table 1, we present nine possible scenarios for estimating gene–environment interplay based on (exogeneity) assumptions for G and E. We also highlight a representative study for each cell (where possible), which we discuss in more detail in Supplementary Appendix D. We distinguish between three possible scenarios for genotype G: (1) exogenous G and a PGI obtained from a parent–child or sibling GWAS (top row); (2) exogenous G and a PGI based on a regular (between-family) GWAS (middle row); and (3) endogenous G and a PGI based on a regular GWAS (bottom row).^9^ Exogenous G refers to a situation where genotyped family data allow one to control for (imputed) parental genotype, or where sibling data are available, allowing one to include family fixed effects, such that the variation in offspring genotypes is randomly assigned.

We also distinguish between three categories for the environmental measure: exogenous (left column), predetermined (middle column), and non-predetermined E (right column; the latter two being endogenous). Predetermined measures of environment E are defined as those set before conception or not caused by genotype G. Examples of predetermined environments E could be family income or air pollution levels before conception. Such environmental exposures are clearly not caused by one’s genes but are likely correlated with other environmental exposures (which we refer to as $E^{*}$) and possibly influenced by parental genotype.

In the following subsections, we discuss each of the nine scenarios. For reasons of space and exposition, we mostly focus on the interpretation and biases in the main effects of G and E and do not separately discuss the G×E interaction term. However, in specific cases where the interpretation of the interaction term does not follow naturally from the main G and E effects, we discuss those separately.

### 3.1. The ideal experiment: exogenous G and exogenous E

Unbiased estimates of the coefficients on G, E, and G×E can be obtained by combining a quasi-experimental design that isolates exogenous variation in environmental exposure with genetic data from multiple family members to control for parental genotype, along with summary statistics from a well-powered parent–child or sibling GWAS to construct the PGI. This scenario, illustrated in the top row, left column of Table 1, is likely to emerge in the near future but does not yet exist. In this ideal experiment, the constructed PGIs are perfect estimates of the genotype G, though measurement error in the PGI may result in downward bias as we highlight in Section 3.2.2. Since exogenous variation in E is commonly used in economics, we focus in our discussion on the estimation of exogenous G.

The source of variation in one’s genotype is well-understood. As stated by Mendel’s first law, one’s genotype is the result of the random segregation of one’s parental genotypes during meiosis. Thus, conditional on parental genotype, the genotype of the child is random. Consider the following simplified relation between the outcome $Y_{i}$ of child i and her genotype $G_{i}$, conditional on the genotype of her mother $G_{m(i)}$ and father $G_{f(i)}$ (Kong et al., 2020):

(2) $$Y_{i}=\beta_{0}+\beta_{G}G_{i}+\beta_{G_{m}}G_{m(i)}+\beta_{G_{f}}G_{f(i)}+\varepsilon_{i},$$

where $\beta_{0}$ is a constant term, $\beta_{G}$ captures the direct genetic effect of the child’s genotype $G_{i}$, $\beta_{G_{m}}$ and $\beta_{G_{f}}$ capture indirect genetic effects from the mother and father, respectively, and $\varepsilon_{i}$ denotes the error term.

We can think of equation (2) in terms of either a GWAS stage or an analysis stage. The analysis stage represents the use of PGIs for the child and parents ($G_{i}$ and $G_{m(i)}∕G_{f(i)}$, respectively) based on results from a GWAS, and applied to explain variation in the outcome of interest $Y_{i}$. The GWAS stage instead reflects a series of J regressions that estimate the $\beta_{j}$ effect sizes for each of J SNPs, conditioning on parental genotype. Following our definition of variant substitution, any association between a genetic variant and the outcome of interest in a GWAS that conditions on parental genotypes reflects a causal genetic effect.

In practice, such analyses do not always require the availability of trios (two parents and one or more children). Pairs of close relatives can be sufficient since one can impute the genotype of the “missing” third individual in a trio (Kong et al., 2020; Young et al., 2022) by leveraging the Mendelian laws of genetic inheritance and the observed alleles of relatives. The aim is to estimate the four parental alleles: two for the mother and two for the father. When all four are observed (*e.g.* the two children have four different alleles), parental genotypes can be accurately reconstructed. When only two or three parental alleles are observed, the missing parental alleles can be imputed, for example, using the average allele frequency in a reference panel—a dataset containing genetic data on a comprehensive sample of individuals with similar ancestry. This approach results in consistent and unbiased estimates of the direct genetic effect (for a detailed proof, see Young et al., 2022).

An alternative strategy to establish causal genetic effects in the GWAS stage is to use a sample of sibling pairs and to run a sibling GWAS by including family fixed effects (Howe et al., 2022). Instead of equation (2), we then have

(3) $$Y_{ij}=\beta_{0j}+\beta_{G}G_{ij}+\varepsilon_{ij},$$

where $Y_{ij}$ is the outcome for individual i in family j, $G_{ij}$ is the genotype of individual i in family j, and $\beta_{0j}$ represents a family fixed effect absorbing the parental genotype.^10^ The analysis compares differences in sibling genotypes $G_{ij}$ to differences in their outcomes $Y_{ij}$. Such analyses exploit the fact that the genotypic variation between siblings is randomly assigned given that siblings draw from the same shared genetic pool: their parental genes.

When PGIs constructed from the results of such a GWAS are applied in a G×E analysis that conditions on parental genotypes, the PGI coefficient represents the causal genetic effect $\beta_{G}$. Indeed, controlling for parental genotypes in the analysis stage fully addresses the confounding that results from passive *rGE*: the links between the grey (causal) and white (confounding) parts in Figure 1 are broken. When combined with an exogenous source of variation in the environment, such analyses would constitute the ideal experiment: when both G and E are exogenous, the estimated effects of the PGI G, the environment E, and their interaction G×E will all be unbiased and can thus be interpreted as causal (Table 1, top row, left column).

One potential advantage of the family fixed effects approach over the parent–child trio approach in the G×E *analysis* stage is that it may render the assumption of exogeneity of the environmental exposure more plausible. However, it also carries four important limitations. First, because the family fixed effects strategy requires at least two siblings from the same family, it cannot be used to study single-child families. Second, when one sibling’s genotype directly affects another sibling’s outcome (sibling effects), this will bias the coefficient of G in a family fixed effects model (see Kong et al., 2020 and Supplementary Appendix C.1 for details). In contrast, bias due to sibling effects does not exist in parent–child trio analyses, since sibling genotypes are randomly assigned conditional on parental genotype, and hence independent of each other.

Third, even though the main genetic effects are identified based on within-family variation in equation (3), the G×E interaction term in these fixed effects regressions might be partially identified from between-family variation (see Shaver, 2019; Giesselmann and Schmidt-Catran, 2022, and Supplementary Appendix C.2 for details). A final limitation is that members of a sibling pair have to be exposed to different exogenously determined environmental circumstances, because the model is identified from variation within families. This obviously puts restrictions on any natural experiment in a sibling approach to studying G×E interplay. In contrast, parent– child trio analyses enable the study of a single exogenous environmental shock affecting a single child or all siblings within a family because such analyses can exploit variation across families.

### 3.2. The current state of the literature

Due to the limited availability of large-scale datasets with parent–child or sibling genotypes, there is currently only one example of a sibling GWAS (Howe et al., 2022) and one of a parent–child GWAS (referred to as a “family GWAS” in Tan et al., 2024). The explanatory power of the resulting PGIs, however, is limited, because the size of the discovery sample is relatively small. As a result, we empirically find ourselves in the middle or bottom row of Table 1.

#### 3.2.1. Exogenous G and a PGI based on a regular GWAS.

When (i) using PGIs from a regular GWAS and (ii) controlling for parental PGIs or family fixed effects in the G×E analysis stage, one generally underestimates the effect of the child’s PGI (see Supplementary Appendix C.3 for details). To understand the intuition, consider a family fixed effects specification that exploits sibling differences in PGIs that are obtained from a regular GWAS. The PGI coefficient not only captures the direct genetic effects but also genetic nurture, assortative mating, sibling effects and ancestry (see Figure 1). Hence, if one sibling carries more SNPs that reflect genetic nurture effects than does the other, the within-sibling PGIs will differ. However, genetic nurture is arguably identical across siblings, reflecting the parental environment shared by siblings. The difference in the estimated PGIs across siblings, therefore, effectively constitutes measurement error. This leads to an attenuation bias in the PGI coefficient (Trejo and Domingue, 2018) and its interaction (see the second row of Table 1), and therefore reflects conservative estimates.^11^ Combining exogenous G with a PGI based on a regular GWAS and random variation in an environment (exogenous E) represents the current “state of the art” and our empirical application is an example of this (see Section 4).

#### 3.2.2. Endogenous G.

Because of the scarcity of genotyped parent–child/sibling data, the PGI is typically endogenous in contemporary G×E analyses (bottom row of Table 1). Hence, even when the environment is exogenous (Table 1, left column, bottom row), G may pick up the effects of parental G and associated environments $E^{*}$ shaped by the genotypes of parents and other ancestors (see Figure 1). As discussed in Trejo and Domingue (2018), the genetic effect $\beta_{G}$ is likely to be biased upward in this case, as the PGI coefficient captures both direct genetic effects and environments shaped by parental genes, which typically have the same sign (*e.g.* childgenetic variants associated with higher EA are generally associated with familial environments more conducive to education). In most cases, the coefficient on the interaction term will also be biased upward. However, since G here reflects the sum of direct genetic effects and passive *rGE*, in theory, they could have opposite signs, leading to bias in an unknown direction.

Although not explicitly included in Table 1, another source of endogeneity in the estimation of the effect of PGIs is measurement error. Since the discovery GWAS samples are not infinitely large, there is measurement error in the estimated *GWAS coefficients* and hence the constructed PGI is a noisy proxy of the “true” PGI (see Supplementary Appendix B.3). A promising way to address the resulting attenuation bias is to apply instrumental variable (IV) techniques. As first suggested by DiPrete et al. (2018), by splitting the GWAS analysis sample into two parts, one can construct two “independent” PGIs in the analysis sample using the two sets of GWAS summary statistics and use these as instruments for one another. The most efficient way to combine this information is to use the “obviously related instrumental variable” method (Gillen et al., 2019; Van Kippersluis et al., 2023). When the discovery and prediction sample come from a very different context, or for small GWAS discovery samples, an alternative approach is offered by Becker et al. (2021), who propose to scale the coefficients of the PGI by a factor based on the SNP-based heritability. These methods are well-established and recommended for purging measurement error in the main effect of the PGI in between-family settings. However, for G×E studies and for within-family settings much remains to be explored.

#### 3.2.3. Predetermined E.

A commonly analysed environmental measure is the childhood environment (*e.g.* area-level unemployment or death rates, distance to facilities, and family income). When such predetermined measures are analysed in a family-based sample (*i.e.* with controls for parental genotype or with family fixed effects) and when the PGI is constructed based on a parent–child or sibling GWAS, any statistically significant G×E interaction coefficient indicates the existence of a “true” G×E effect. The intuition is that by virtue of the family-based nature of the GWAS and analysis stage, the measure of G is unbiased and “randomized” with respect to the environment. Detecting a G×E interaction, therefore, implies the existence of such an interaction rather than a G×G or E×E interaction. However, predetermined environmental characteristics tend to cluster together. For example, areas with high unemployment rates tend to have fewer facilities; and family income is strongly associated with parental education and occupation. When G×E is identified, the interaction term may therefore reflect $G\times E^{*}$, where $E^{*}$ is some unobserved correlate of the putative environmental characteristic E (Table 1, top row, middle column).

When parent–child/sibling GWAS results are not available for constructing the PGI, but the analysis stage includes controls for parental genotype or family fixed effects, the coefficients on G and G×E will be downward biased (Table 1, middle row, middle column). The reason for the downward bias is identical to the case of exogenous E (Table 1, middle row, left column).

Without access to family-based datasets (neither in the GWAS nor the analysis stage), the predetermined environmental characteristic E may reflect parental G (Table 1, bottom row, middle column) as a result of familial influences (passive *rGE*). For example, if parental G influences the location of residence or family income E, this may lead to bias in an unknown direction. As before with endogenous G (Table 1, bottom row, left column), the coefficient of G is upward biased since the PGI captures both direct genetic effects and environments shaped by parental genes, which typically have the same sign.

Most existing studies fall into the category of endogenous G and predetermined E (see Supplementary Figure G.1 in Appendix G). Therefore, a possible correlation between G and E—often family SES—is arguably the most important source of bias in existing G×E applications. This form of bias is not difficult to imagine: with assortative mating based on a moderately heritable trait like EA, a correlation between G and socioeconomic advantage naturally arises over generations (Abdellaoui et al., 2022). It is not straightforward to assess the magnitude of this correlation, because virtually all existing PGIs capture both direct as well as indirect genetic effects, the latter being itself an environmental component. This biases any correlation between the PGI and family socioeconomic advantage upward. At the same time, measurement error in existing PGIs biases the correlation downward. Despite these challenges, evidence from Papageorge and Thom (2020) and Ronda et al. (2022) suggests that although a significant (yet modest) correlation exists—*e.g.* individuals from high and low SES families differ on average 0.20 (0.27) of a standard deviation in the EA PGI in the US (Denmark)—in both cases the more striking observation is the substantial overlap in the distribution of the EA PGI across individuals from advantaged and disadvantaged socioeconomic groups (see Figure 3 in Papageorge and Thom, 2020, and Figure 1 in Ronda et al., 2022).

#### 3.2.4. Non-predetermined E.

The last column of Table 1 presents the case of non-predetermined E. Endogeneity of E may arise from five sources: reverse causality, omitted variable bias, measurement error, mediation, and correlation of the GWAS sample selection with the analysed environment E. The first three sources of endogeneity are common to many econometric analyses (see, among others, Wooldridge, 2002; Angrist and Pischke, 2008; Cunningham, 2021), and so we relegate a detailed discussion of these possible biases to Supplementary Appendix C.

Non-predetermined E can lead to an endogeneity problem through mediation. For example, E can be shaped by G through active or evocative *rGE*. In this case, if the environment to which one is exposed is partially shaped by one’s genes, E essentially becomes a “bad control” (*i.e.* E is itself an outcome of G) in the relationship between Y and G.^12^ It is no longer clear whether the coefficients on E and G×E genuinely reflect policy-relevant parameters (Wagner et al., 2013). This can lead to spurious detection of a G×E effect when, in fact, one is measuring the effect of G×G (if E is shaped by one’s genes through active *rGE*) or of $G\times E^{*}$ (through correlated environments). These scenarios are presented in the last column of Table 1.

Endogeneity in G×E analyses can also arise when the treatment group in the analysis sample closely mirrors the GWAS discovery sample, even without overlap between the samples. This resemblance can lead to significant G×E effects being estimated, even if no true interaction exists, because the GWAS-based PGI reflects the genetic effects within the environmental context of the original GWAS sample. If the treatment group is more similar to this context, G may be more predictive among the treated group than the controls, leading to an apparent G×E . This issue can occur even if the environment E is exogenous.^13^ To check for this, one can test if G differs across environments, akin to testing for *rGE*, but focused on exogenous environments. If *rGE* is found, the environment may actually be endogenous to the GWAS sample. The solution is to use alternative GWAS summary statistics that are not endogenous to the environment of interest.

#### 3.2.5. Summary.

Our discussion of the nine scenarios in Table 1 shows that one can keep bias in G×E analyses manageable when exploiting exogenous variation in both genetics and environments. Exogenous variation in E can be analysed with the usual toolkit available to the applied econometrician: randomized controlled trials, difference-in-differences methods, regression discontinuity designs (RDDs), instrumental variables, or other (quasi-)experimental methods (Schmitz and Conley, 2017a). In this way, the environmental measure is independent of G, and one can draw causal conclusions from the environmental exposure. With current within-family GWAS samples too small to construct PGIs, for the time being regular (between-family) GWAS results will be used to construct PGIs. The coefficient of G in such analyses will be upward or downward-biased depending on whether family- or population-based analysis samples are used. In a within-family setting, the effect of G can be interpreted as causal, running through biological and/or environmentally driven (*i.e.* through active or evocative *rGE*) pathways (Figure 1).

### 3.3. Functional form

The literature on G×E interplay typically specifies G as a continuous PGI, which in turn is interacted with a certain environmental exposure. At first sight, it may appear incorrect to construct a PGI based on an additive GWAS model and use this additive index to test for non-linearities with a certain environmental exposure. However, starting from a less restrictive linear mixed model, Miao et al. (2022) show that the common practice of modelling G×E using PGI × E interactions has a clear interpretation in terms of an underlying data generating process with SNP main effects and SNP × E interactions. The coefficient of the PGI × E term is proportional to the covariance between the SNP coefficients and the SNP × E interaction coefficients. Therefore, the interaction term in typical PGI × E applications captures systematic linear amplification or reduction of the SNP main effect across the entire genome by the environmental moderator E.^14^

It therefore follows that measuring G as a continuous variable, *i.e.* as a PGI, is currently the natural starting point. The relevant PGI is typically pinned down by the choice of the outcome of interest. For example, if one studies EA, a natural choice for G is the PGI for education. However, this need not always be the case. Any PGI could be used if warranted by theory or for empirical reasons. For example, the PGI for EA has been shown to predict a variety of outcomes, such as social mobility and wealth (Belsky et al., 2016, 2018; Barth et al., 2020; Papageorge and Thom, 2020). Because the PGI has no natural metric, its effects are typically reported in standard deviations on an underlying latent scale of (loosely speaking) genetic propensity (Becker et al., 2021).

A final consideration is how to model the outcome variable. Most existing applications adopt linear regressions because of the well-known challenges with interaction terms in non-linear specifications (Ai and Norton, 2003). However, when the prevalence of a binary outcome is relatively low, adopting a linear probability model may lead to spurious G×E effects (see, *e.g.* Domingue et al., 2020, 2024 for details).

## 4. AN EMPIRICAL ILLUSTRATION OF G×E INTERPLAY: SCHOOL STARTING AGE AND TEST SCORES

Our empirical application examines a school entry policy in England, where month of birth (MoB) determines when pupils start school. They start school in the school year (the period beginning September 1st and ending August 31st) in which they turn five. At one extreme, children born on August 31 start their primary schooling when they are 4 years and 1-day-old, whereas those who are born one day later, on September 1, start on their fifth birthday. Hence, the latter group is a full year (minus one day) older than students born on August 31. At age 4, this is 25% of their lifetime—a non-negligible difference.

We study the effect of this English school entry policy on skills measured by standardized (so-called Key Stage) tests administered during years 2, 6, 9, and 11 of formal schooling (*i.e.* ages 6–7, 10–11, 13–14, and 15–16) as well as a test taken before children start school, which we refer to as the Entry Assessment test. A student’s MoB induces quasi-exogenous variation in the policy environment (E) shaping student performance on these tests (Y). In particular, the policy dictates that some students enter school later and take these tests when they are older than other students. This treatment, therefore, (i) shifts the age at which students take the test, (ii) changes the amount of developmental time spent at home, and (iii) alters the relative age of students compared to their peers. We ask whether exposure to this treatment affects students differently based on their genetic endowments measured by the PGI for EA (G).^15^

### 4.1. Background and theoretical framework

The existing literature provides evidence that age at entry policies matter for pupils’ outcomes: older pupils perform better on educational tests than their younger counterparts in the same grade (see, *e.g.* Fredriksson and Ockert, 2005; Bedard and Dhuey, 2006; Crawford et al., 2010; Black et al., 2011; Ritchie and Tucker-Drob, 2018). This has significant long-term implications (Page et al., 2019) such as for the likelihood of attending university (Bedard and Dhuey, 2006) and individual earnings (Fredriksson and Ockert, 2005). Whether effects are heterogeneous across child or family characteristics is less clear. Some studies report differences by child gender (*e.g.* Cornelissen and Dustmann, 2019), while others do not (*e.g.* Fredriksson and Ockert, 2005). Black et al. (2011) find little evidence of heterogeneous effects by socioeconomic status for IQ, mental health, teen pregnancy, education, or social assistance receipt, while Fredriksson and Ockert (2005) find stronger effects among children with lower educated parents, suggesting that a later starting age is particularly beneficial among those from disadvantaged backgrounds.

Several possible mechanisms could give rise to these effects (see Crawford et al., 2010). First, as exams are generally taken at a fixed date, any differences can be due to *absolute age effects* (or test age effects): older pupils in the school year will be up to one full year older at the time they sit the exam than their younger counterparts. Second, there can be a direct effect of starting formal schooling too early, when pupils are simply not yet ready. This is referred to as the *school starting age effect*. Third, there may be a *length of schooling effect*, particularly in school systems that allow pupils to start later in the year depending on their date of birth. With a fixed exam date, these pupils will have experienced a shorter length of schooling compared to those who start at the beginning of the school year. Finally, there may be a direct effect on your test scores of being younger than your peers when starting school; the *relative age effect*.

The strongest evidence is found for absolute age effects as a mechanism through which age at entry impacts on student outcomes (Crawford et al., 2010; Black et al., 2011). Other school-related age effects also appear to play a role in pupils’ performance, albeit with smaller impacts. For example, examining the effect of *school starting age* net of any absolute age effects, Black et al. (2011) find that, although a later starting age does not impact EA, it does lead to better mental health at age 18 and negatively impacts individuals’ earnings, an effect that disappears by age 30. Starting school older also reduces the probability of teenage pregnancy. Similarly, Cornelissen and Dustmann (2019) focus on identifying the *length of schooling effect* in a setting with universal early schooling (England), conditional on age at entry. They find that increasing exposure to schooling by starting school earlier increases pupils’ cognitive and non-cognitive outcomes at early ages, but only the effects on non-cognitive skills persist (at least until age 11). Effects are larger for boys (but not girls) in low socioeconomic status households.

Here, we introduce a stylized economic model of skill accumulation, parental investment, and test outcomes. The model distinguishes between skill at school entry and skill as students progress through school, and highlights how a particular environmental exposure (school starting age policy) can interact with genetic endowments to produce G×E effects through a variety of channels. Let us consider a school system where at the end of τ periods of schooling, students take an exam which measures their accumulated skill, $\theta_{i}^{\tau }$. This is our key outcome of interest. We assume that $\theta_{i}^{\tau }$ is determined by a dynamic skill accumulation process with two distinct stages: time at home before school entry, and the τ years spent in the formal schooling system. Let $a_{i}^{e}$ represent a student’s age at school entry, which can be thought of as our environmental exposure $E_{i}$. Some children are randomly assigned an “old” starting age ($a_{i}^{e}=5$), while others are assigned a “young” starting age, ($a_{i}^{e}=4$). Also, let $G_{i}$ represent the child’s genetic endowment, and let $\theta_{i}^{e}$ refer to the child’s stock of skills at school entry.

#### Skill accumulation before entry:

Age at entry can impact later schooling outcomes through several potential channels, the first of these being the effect of age at entry on the stock of skills that students possess when they enter formal schooling, $\theta_{i}^{e}$. Let $\theta_{i4}^{e}$ denote the stock of skills the student would have at entry if they were assigned entry at age 4, and let $\theta_{i5}^{e}$ denote the stock under assigned entry at age 5. If a child enters school at age 4, we assume entry skills are an increasing function of genes: $\theta_{i4}^{e}=\theta_{i4}^{e}(G_{i})$. However, if a child enters school at age 5, then $\theta_{i5}^{e}$ emerges as a function of $\theta_{i4}^{e}(G_{i})$ (initial skills at age 4), genotype ($G_{i}$), and parental investments between ages 4 and 5, $x_{i5}$. This technology is summarized by the production function:

(4) $$\theta_{i5}^{e}=\theta_{i5}^{e}(\theta_{i4}^{e}(G_{i}),G_{i},x_{i5}).$$

#### Skill accumulation during formal schooling:

Once students enter formal schooling, their skills evolve as a function of skill at entry, $\theta_{i}^{e}$, their genetic endowments, $G_{i}$, and their age at entry, $a_{i}^{e}$:

(5) $$\theta_{i}^{\tau }=\theta_{i}^{\tau }(\theta_{i}^{e},G_{i},a_{i}^{e}).$$

Here, $\theta_{i}^{\tau }(\cdot )$ can be interpreted as a stylized summary of a more complicated dynamic skill accumulation process throughout the τ years of schooling. We assume that $\theta_{i}^{\tau }$ is increasing and concave in entry skills $\theta_{i}^{e}$ and in the genetic endowment $G_{i}$ (the two continuous inputs): $\frac{\partial \theta_{i}^{\tau }}{\partial \theta_{i}^{e}}>0$, $\frac{\partial^{2}\theta_{i}^{\tau }}{\partial \theta_{i}^{e2}}<0$, $\frac{\partial \theta_{i}^{\tau }}{\partial G_{i}}>0$, $\frac{\partial^{2}\theta_{i}^{\tau }}{\partial G_{i}^{2}}<0$.^16^ For simplicity, we abstract from several sources of complexity that may be important. First, we do not model the role of parental characteristics and parental investments during school. Parental genetic factors $G_{i}^{p}$ could be an additional input in the production function, giving rise to the various sources of confounding discussed in Section 3.2. We also do not explicitly model peer effects, except that entry age $a_{i}^{e}$ could affect skill production through a relative age effect (*e.g.* benefiting from being surrounded by older or younger peers, or because they receive different attention from teachers). Finally, we also abstract from the role that siblings might play in this process.

#### Optimization problem:

The only choice variable in the model is parental investment between ages 4 and 5, if the child is assigned to $a_{i}^{e}=5$. Parents choose $x_{i5}$ to maximize child i’s school test score $\theta_{i}^{\tau }=\theta_{i}^{\tau }(\theta_{i}^{e},G_{i},a_{i}^{e})$ net of a convex cost function $C_{i}(x_{i5},G_{i})$. That is, the parental decision problem can be formalized as maximizing the following value function V:

(6) $$V_{i}=\max_{x_{i5}}\;\theta_{i}^{\tau }\;(\theta_{i5}^{e}(\theta_{i4}^{e}(G_{i}),G_{i},x_{i5}),G_{i},5)-C_{i}(x_{i5},G_{i}).$$

Limiting the scope for parental action to ages 4–5 is no doubt a simplification, but still allows for endogenous parental choices to shape the differential effect of school entry age across genotypes. A more detailed version of the model would endogenize investment throughout early childhood, and would also recognize the trade-offs that parents face when choosing how to invest across multiple children with potentially different endowments.^17^

The first-order condition for an optimum requires:

(7) $$\frac{\partial \theta_{i}^{\tau }}{\partial \theta_{i}^{e}}(\theta_{i5}^{e},G_{i},5)\frac{\partial \theta_{i5}^{e}}{\partial x_{i5}}(\theta_{i4}^{e},G_{i},x_{i5})-\frac{\partial C_{i}}{\partial x_{i5}}(x_{i5},G_{i})=0.$$

The solution to the first-order condition gives rise to the policy function $x_{i5}^{*}=x_{i5}^{*}(G_{i})$. Also, let $\theta_{i5}^{e*}=\theta_{i5}^{e}(\theta_{i4}^{e}(G_{i}),G_{i},\theta_{i5}^{*}(G_{i}))$ refer to the endogenous level of entry skills for those assigned to $a_{i}^{e}=5$, given the optimal investment choice. In this model, the treatment effect of moving a child from school entry at $a_{i}^{e}=4$ to $a_{i}^{e}=5$ on skills $\theta_{i}^{\tau }$ at time τ is given by

(8) $$\Delta_{i}^{\theta }=\theta_{i}^{\tau }(\theta_{i5}^{e*},G_{i},5)-\theta_{i}^{\tau }(\theta_{i4}^{e},G_{i},4).$$

Utilizing a first-order linear approximation of the function $\theta_{i}^{\tau }(\cdot )$ around $\theta_{i}^{e}=\theta_{i4}^{e}$,^18^ and rearranging equation (8), we obtain

(9) $$\Delta_{i}^{\theta }=\underset{\text{Pre-entry skill accumulation effect}}{\underset{︸}{\left(\theta_{i5}^{e*}-\theta_{i4}^{e})\frac{\partial \theta_{i}^{\tau }}{\partial \theta_{i}^{e}}(\theta_{i4}^{e},G_{i},5\right)}}+\underset{\text{Technological age effect}}{\underset{︸}{\theta_{i}^{\tau }(\theta_{i4}^{e},G_{i},5)-\theta_{i}^{\tau }(\theta_{i4}^{e},G_{i},4)}}.$$

The treatment effect of age at entry is comprised of two distinct parts. The first is a pre-entry skill accumulation effect. Individuals who start formal schooling 1 year later will have accumulated more skills at home before entry, $\theta_{i5}^{e*}-\theta_{i4}^{e}$, and these extra skills will produce more later-stage skills at the rate $\frac{\partial \theta_{i}^{\tau }}{\partial \theta_{i}^{e}}$ ($\theta_{i4}^{e}$, $G_{i}$, 5). However, children who start school later will have other advantages besides a higher initial stock of skills. Age itself may be an input in the human capital production function while in school, or it may increase the productivity of other inputs. For example, an extra year of maturity or biological age could increase the marginal productivity of teacher time or quality. We call this the technological age effect, which is represented by $\theta_{i}^{\tau }(\theta_{i4}^{e},G_{i},5)-\theta_{i}^{\tau }(\theta_{i4}^{e},G_{i},4)$. As a result of this effect, two children starting schooling at different ages might end up with different skills after τ periods, even if they both entered with the same stock $\theta_{i4}^{e}$.

We next differentiate equation (8) with respect to the genetic endowment $G_{i}$ to characterize possible interactions between genotype and age at entry in skill accumulation, which yields:

(10) $$\frac{\partial \Delta_{i}^{\theta }}{\partial G_{i}}=\frac{\partial \theta_{i}^{\tau }}{\partial \theta_{i}^{e}}(\theta_{i5}^{e*},G_{i},5)\frac{d\theta_{i5}^{e*}}{dG_{i}}+\frac{\partial \theta_{i}^{\tau }}{\partial G_{i}}(\theta_{i5}^{e*},G_{i},5)\;\;-\frac{\partial \theta_{i}^{\tau }}{\partial \theta_{i}^{e}}(\theta_{i4}^{e},G_{i},4)\frac{d\theta_{i4}^{e}}{dG_{i}}-\frac{\partial \theta_{i}^{\tau }}{\partial G_{i}}(\theta_{i4}^{e},G_{i},4)\;,$$

where $\frac{d\theta_{i5}^{e*}}{dG_{i}}$ is the total derivative, given that $\theta_{i5}^{e*}$ is a function of $\theta_{i4}^{e}(G_{i})$, $G_{i}$ and $x_{i5}$ (see equation (4)), whereas $\theta_{i4}^{e}$ is only a function of $G_{i}$. After a few manipulations, aimed at separating distinct skill and age at school entry effects,^19^ we arrive at the following derivative, which decomposes the theoretical object we seek to estimate:

(11) $$\frac{\partial \Delta_{i}^{\theta }}{\partial G_{i}}=\underset{(1)\;\text{Differential pre-entry accumulation}}{\underset{︸}{\frac{\partial \theta_{i}^{\tau }}{\partial \theta_{i}^{e}}(\theta_{i4}^{e},G_{i},4)\left[\frac{d\theta_{i5}^{e*}}{dG_{i}}-\frac{d\theta_{i4}^{e}}{dG_{i}}\right]}}+\underset{(2)\;\text{Effect on productivity of entry skills}}{\underset{︸}{\left[\frac{\partial \theta_{i}^{\tau }}{\partial \theta_{i}^{e}}(\theta_{i5}^{e*},G_{i},5)-\frac{\partial \theta_{i}^{\tau }}{\partial \theta_{i}^{e}}(\theta_{i4}^{e},G_{i},4)\right]\frac{d\theta_{i5}^{e*}}{dG_{i}}}}\;\;+\underset{(3)\;\text{Entry skill}-\text{Gene interaction}}{\underset{︸}{\frac{\partial^{2}\theta_{i}^{\tau }}{\partial G_{i}\partial \theta_{i}^{e}}(\theta_{i4}^{e},G_{i},5)[\theta_{i5}^{e*}-\theta_{i4}^{e}]}}+\underset{(4)\;\text{Age}-\text{Gne interaction}}{\underset{︸}{\frac{\partial \theta_{i}^{\tau }}{\partial G_{i}}(\theta_{i4}^{e},G_{i},5)-\frac{\partial \theta_{i}^{\tau }}{\partial G_{i}}(\theta_{i4}^{e},G_{i},4)}}.$$

There are four separate channels through which such an interaction might operate. The first term reflects differential pre-entry skill accumulation. An extra year at home before formal schooling could differentially affect a child’s skill at entry depending on their genotype, captured by $\frac{d\theta_{i5}^{e*}}{dG_{i}}-\frac{d\theta_{i4}^{e}}{dG_{i}}$. Individuals with higher genetic endowments $G_{i}$ might accumulate skills faster during an extra year at home, even given the same level of inputs. In addition, differences in $G_{i}$ could induce differences in parental investment before entry, $x_{i5}^{*}$, which in turn impacts the level of (entry) skills. This can be seen by noting that $\frac{d\theta_{i5}^{e*}}{\partial G_{i}}=\left(\frac{\partial \theta_{i5}^{e*}}{\partial \theta_{i4}^{e}}\frac{d\theta_{i4}^{e}}{dG_{i}}+\frac{\partial \theta_{i5}^{e*}}{\partial G_{i}}+\frac{\partial \theta_{i5}^{e*}}{\partial x_{i5}}\frac{dx_{i5}^{e*}}{dG_{i}}\right)$, which thus includes the parental behavioural response $\frac{dx_{i5}^{e*}}{dG_{i}}$. As we discuss in Section 2 such evocative *rGE* represents a mediator and is part of the causal effect of interest. These differences in entry skill formation, in turn, translate into differences in skills at time τ, reflected in $\frac{\partial \theta_{i}^{\tau }}{\partial \theta_{i}^{e}}$. This first term in equation (11), operating through the production $\theta_{i}^{e}$ of school entry skills (equation (4)), captures the school starting age effect described above.

The second term in equation (11) is a source of G×E interaction arising from an effect on the productivity of entry skills. Starting school older could have an effect on the marginal productivity of entry skills: $\frac{\partial \theta_{i}^{\tau }}{\partial \theta_{i}^{e}}(\theta_{i5}^{e*},G_{i},5)-\frac{\partial \theta_{i}^{\tau }}{\partial \theta_{i}^{e}}(\theta_{i4}^{e},G_{i},4)$. Children who are old-for-grade (regardless of genotype) could be more mature in ways that either increase or decrease the productivity of skills at entry. For example, more mature children could evoke more favourable attention from teachers or peers, which may complement their existing skills at entry. Since children with higher values of $G_{i}$ will have higher starting skills on average ($\frac{\partial \theta_{i5}^{e*}}{\partial G_{i}}>0$), such a boost in the productivity of starting skills could generate a positive interaction between $G_{i}$ and $E_{i}$. However, it is also plausible for this interaction to be *negative*. For example, the maturity that comes with older age could flatten the entry skill versus completed skill relationship if it allows older children to more easily ask questions or seek help when struggling.

The third term in equation (11) reflects a possible interaction between entry skills and genotype in the production function while in school. Suppose that all children increase entry skills $\theta_{i}^{e}$ by a homogenous $\theta_{i5}^{e*}-\theta_{i4}^{e}$ during an extra year at home. These extra skills could add more or less to later-life skills for children with different levels of $G_{i}$, depending on the strength of complementarity in skill production between pre-entry skills and genotype, $\frac{\partial^{2}\theta_{i}^{\tau }}{\partial G_{i}\partial \theta_{i}^{e}}$. If $G_{i}$ and $\theta_{i}^{e}$ are complementary—*i.e.* children with higher $G_{i}$ are better able to use existing skills to produce new skills—then we would expect this to generate a positive G×E interaction. However, it may also be the case that $\frac{\partial^{2}\theta_{i}^{\tau }}{\partial G_{i}\partial \theta_{i}^{e}}<0$. Higher values of $G_{i}$ might reduce the relationship between entry skills and completed skills if those with higher $G_{i}$ can more easily overcome deficits in initial skill.

Finally, the fourth term in equation (11) highlights an interaction that does not involve the influence of skills produced at-home before entry. Individuals with different levels of $G_{i}$ could experience systematically different effects of biological maturation on skill accumulation, holding entry skills constant. This difference in the technological age effect could arise for several reasons with ambiguous implications for the sign of G×E interactions here. For example, endowments $G_{i}$ could affect brain function in ways that become more pronounced over time (or whose effects compound over time), regardless of entry starting skills. It may also be that higher values of $G_{i}$ make current biological age less important. Students that more easily learn (perhaps because of higher $G_{i}$) may gain less from a later entry if such an ability can substitute for the benefits of being old-for-grade (*e.g.* more advantageous interactions with teachers and peers).

Equation (11) highlights two important points to consider when interpreting econometric studies of G×E. First, such interactions at least partially arise from the endogenous choices of optimizing agents. In particular, in our very stylized model, parents react to an exogenous change in age at school entry by adjusting their parental inputs $x_{i5}^{*}$ during time spent at home. Since G×E interactions are not purely technological parameters, better understanding of how and why these interactions arise (and what they imply for policy) requires economic theory. A second point is that G×E results are useful for researchers interested in the technology of skill formation and the behavioural processes that shape it. The treatment effect of moving a child from school entry age 4 to age 5 and its interaction with genotype, $\frac{\partial \Delta_{i}^{\theta }}{\partial G_{i}}$, depends on general features of the production technology, including the marginal productivity of entry skills, the marginal productivity of parental investments, and the effect of age. Estimates of $\frac{\partial \Delta_{i}^{\theta }}{\partial G_{i}}$ (together with other analyses) may thus shed light on important features of the production technology, regardless of whether one has an interest in genetics or not.

One final insight is that there is a potentially important distinction between G×E interactions in determining an outcome and G×E interactions in determining welfare. Equation (11) accounts for the sources of G×E interactions for the observable outcome of test scores $\theta_{i}^{\tau }$. However, agents in this model care not only about the production of skills, but also about the costs associated with the investment. Indeed, one could also consider expressing G×E interactions in terms of the value function $V_{i}$ (equation (6)). Here, that would be

(12) $$\frac{\partial \Delta_{i}^{V}}{\partial G_{i}}=\frac{\partial \Delta_{i}^{\theta }}{\partial G_{i}}-\frac{\partial C_{i}}{\partial x_{i5}}\frac{\partial x_{i5}}{\partial G_{i}}-\frac{\partial C_{i}}{\partial G_{i}}.$$

The literature often interprets the presence of G×E as an effect on inequality. Finding $\frac{\partial \Delta_{i}^{\theta }}{\partial G_{i}}<0$ (equation (8)) for a particular policy might be interpreted normatively as demonstrating that the policy reduced inequality due to genetic factors. While this may be true at the level of the outcome, there may be fundamentally different implications for the level of welfare, which might be the more important margin, depending on the preferences of a policy maker.

### 4.2. Data

We investigate the effect of age at entry using the Avon Longitudinal Study of Parents and Children (ALSPAC). ALSPAC is a cohort study in which pregnant women living in Avon (U.K.) with an expected delivery date between 1 April 1991 and 31 December 1992 were invited to take part. The initial number of pregnancies was 14,541, including 14,676 foetuses. Among these, 13,988 children were alive at age 1.^20^

We use ALSPAC for three main reasons. First, the cohorts covered by the data had to abide by strict rules on when children could start school relative to their date of birth,^21^ resulting in a strong discontinuity in children’s starting age between those born just before and just after the threshold of September 1. Although births are often planned, implying that MoB is a choice variable and therefore endogenous, we find no evidence that being born just before or after the end of August is systematically related to children’s or parental background characteristics; we show this below. Second, the majority of cohort members, a substantial number of their mothers, and a small number of fathers have been genotyped. We exploit this feature of the data and use the software package snipar (Young et al., 2022) to impute the remaining paternal genotypes using data on the cohort members and their mothers, allowing us to optimally utilize ALSPAC’s family-design. We create the EA PGI by meta-analysing GWAS summary statistics from the UK Biobank and 23andMe, correcting for linkage disequilibrium (LD) between SNPs with the software package LDpred (Vilhjalmsson et al., 2015).^22^ We standardize the PGI to have mean 0 and standard deviation 1 in the analysis sample. Third, ALSPAC contains an extremely rich set of child outcomes, including from administrative sources. Specifically, we use children’s performance on exams taken at five time points. We use an Entry Assessment test, taken by all pupils about to start primary school (at ages 4–5; *i.e.* before the start of formal schooling), and four nationally set examinations taken in Years 2, 6, 9, and 11 of formal schooling (ages 6–7, 10–11, 13–14 and 15–16), also known as Key Stage 1 (KS1), 2 (KS2), 3 (KS3), and 4 (KS4 or GCSE) examinations, respectively. Children’s scores are obtained from the National Pupil Database, a census of all pupils in England within the state school system, which is matched to ALSPAC.^23^ For each of the tests, we use an average score for the child’s mandatory subjects,^24^ which we standardize to have mean 0 and standard deviation 1.

### 4.3. Identification strategy

To identify the effect of age at entry, we use a RDD, specifying the treated and control groups as pupils born after and before September 1, respectively. To empirically check the validity of our identification strategy, we begin by exploring the raw data, plotting the trends in test scores by MoB and examining the correlations between treatment, test scores, and the PGI.

**Figure 2:**
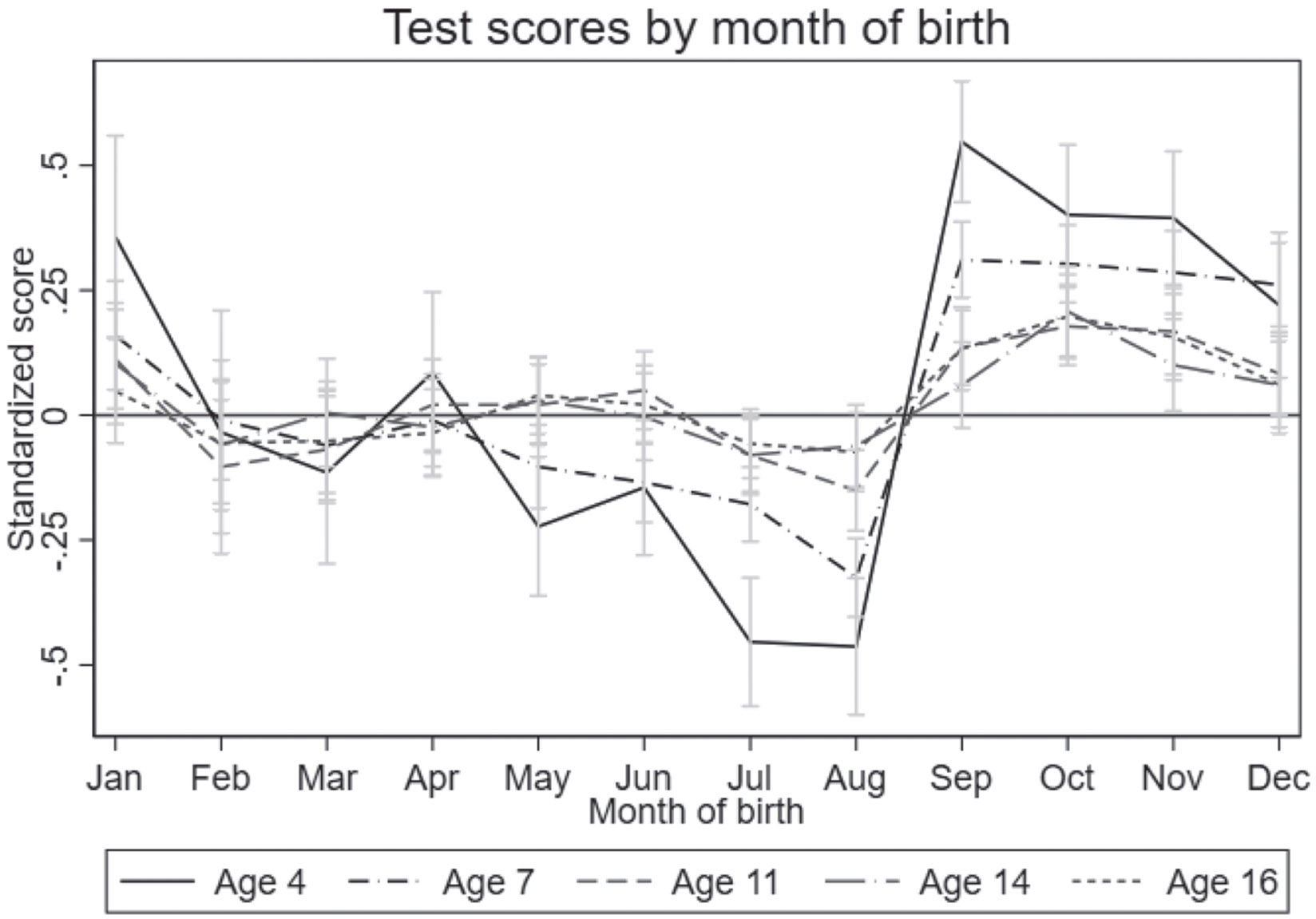
Standardized test scores at different ages by MoB

Figure 2 presents standardized test scores at ages 4–5, 6–7, 10–11, 13–14, and 15–16 as a function of pupils’ MoB. It shows a clear discontinuity in test scores for those born in September and beyond in comparison to those born before September, with the latter group performing significantly worse on all five tests: at ages 4–5, those born in September perform approximately one standard deviation better than their August-born peers. This difference reduces as children age (*i.e.* as the relative difference in age across the cut-off decreases), but it remains sizable and statistically significant across all assessments. Our main interest is in the discontinuity in test scores at the cut-off and in investigating whether (and how) this varies with an individual’s genetic predisposition for EA. Thus, in the empirical analyses, we restrict the sample to those born between June and November: three months before and three months after the threshold.^25^

To explore whether one’s MoB is as good as random, Table 2 reports descriptive statistics for a set of pupil and family characteristics by treatment status. We include only covariates observed before (or at) birth, as any variables measured later in life could be directly or indirectly affected by the treatment. Although Table 2 shows some significant differences, they are very small and do not survive corrections for multiple hypothesis testing. There are also no differences by treatment status in the child’s, mother’s, or father’s PGI for education (*i.e.* there is no evidence of *rGE*; bottom three rows). This suggests that individuals’ MoB is unrelated to the various child and family background characteristics observed here.

To more formally check for *rGE*, we explore whether the treated and control groups have systematically different PGIs, which would suggest selection into treatment based on genotype. The top panel of Supplementary Figure G.1 in Appendix G plots the density of the child’s PGI for the treatment and control groups, showing little difference in their distributions. The bottom left and right-hand panels present the PGI for the children’s mothers and fathers, respectively, by treatment and control group, showing similarly overlapping distributions. The polychoric correlations between the treatment indicator and the child’s PGI (ρ=−0.013, s.e. 0.019), the maternal PGI (ρ=0.009, s.e. 0.023), and the paternal PGI (ρ=−0.021, s.e. 0.023) are very small and statistically indistinguishable from zero. This suggests there is no gene–environment correlation.

### 4.4. Predictive power of the PGI

Table 3 explores the predictive power of the PGI for the five test scores at different ages. Panel A presents the estimates without controls for the parental PGIs, showing that a one standard deviation increase in the child’s PGI is associated with an increase in test scores of 0.163 standard deviations at ages 4–5, 0.255 standard deviations at ages 6–7 (KS1), and between 0.318 and 0.357 standard deviations at ages 10–16, suggesting that the predictive power of the PGI increases with child age. The PGI explains between 8.5% and 13.4% of the variation in test scores.

Panel B of Table 3 shows the same estimates, additionally adjusting for the maternal and (imputed) paternal PGIs. This shows somewhat smaller, but surprisingly similar, point estimates of the child’s PGI, which again increase with child age. The maternal PGI explains additional variation in child test scores, but the paternal PGI does not, except for Key Stage 1. This may be explained by increased measurement error in the paternal PGI due to imputation, but could also reflect that the maternal PGI is more important in shaping child outcomes.^26^

### 4.5. Functional form

**Figure 3:**
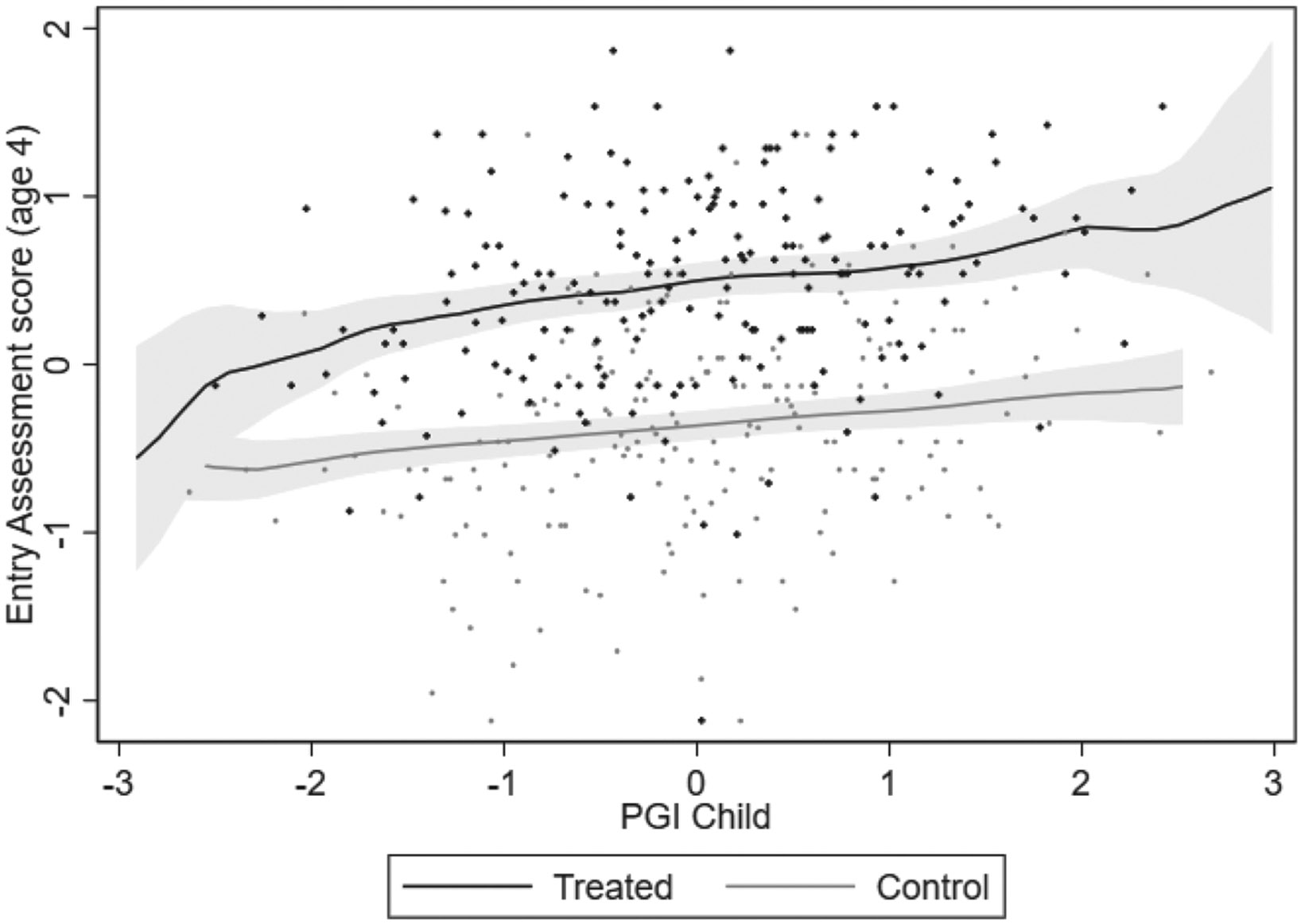
The relation between PGI Child and the Entry Assessment (ages 4–5) test score in the treatment and control group *Notes:* Black dots refer to the treated group; grey dots to the control group. The PGI distribution is trimmed to be between −3 and +3 to avoid non-linear overfitting of outliers.

We motivate the functional form used in our main analysis by graphically examining the relationship between the PGI and test scores for our treatment (September–November borns) and control (June–August borns) group. We illustrate this with the ages 4–5 Entry Assessment. Figure 3 plots the non-parametric relationship between the PGI and pupils’ performance on this test, distinguishing between treatment and control groups. The figure shows that both relationships are positive, with some suggestion that the slope is slightly steeper for the treatment group. Furthermore, the relationship between the PGI and the outcome is approximately linear for both the treatment and control group. We, therefore, use a linear specification in our main analysis. However, we also explore the robustness of our results to non-linearities in the PGI in Supplementary Appendix F.

### 4.6. Empirical specification

We are interested in the discontinuity in test scores between those born before and after September. To estimate the main effect of the PGI ($PGI_{i}$) and treatment status ($Treated_{i}$) as well as their interaction, we adopt a standard RDD:

(13) $$TestScore_{i}=\delta_{0}+\delta_{G}PGI_{i}+\delta_{E}Treated_{i}+\delta_{G\times E}(PGI_{i}\times Treated_{i})\;\;+\delta_{1}MoB_{i}+\delta_{2}(MoB_{i}\times PGI_{i})+\delta_{3}(MoB_{i}\times Treated_{i})\;\;+\delta_{4}(MoB_{i}\times PGI_{i}\times Treated_{i})+\delta_{5}(X_{i},PGI_{i},Treated_{i})\;\;+\delta_{6}\left(\sum_{p=1}^{10}{PC}_{i}^{p},PGI_{i},Treated_{i}\right)+e_{i},$$

where $TestScore_{i}$ is the test score for child i, $PGI_{i}$ is pupils’ EA PGI and $Treated_{i}$ is the environment of interest: a dummy that equals one for treated individuals and zero for the controls; $MoB_{i}$ is the running variable, capturing the trend in $TestScore_{i}$ by MoB for those born before September (note that this variable runs from −3 to 2, capturing the calendar months June–November, with September set to 0). The coefficients for $MoB_{i}\times Treated_{i}$ and $MoB_{i}\times PGI_{i}$ capture changes in slope for those born from September onwards and those with higher PGIs, respectively. The control variables $X_{i}$ include a gender dummy, a dummy for birth in 1992 (capturing potential differences in test scores between the birth cohorts), and the first ten principal components of the genetic data $\left(\sum_{p=1}^{10}PC_{i}^{p}\right)$. We also include interactions between the main effects and all (demeaned) controls $X_{i}$, represented by $\delta_{5}(\cdot )$ and $\delta_{6}(\cdot )$, as suggested by Keller (2014) and Feigenberg et al. (2023).^27^ Hence, the coefficient for $PGI_{i}$, $\delta_{G}$, captures the change in test scores associated with a one standard deviation increase in the PGI for the control group (E=0) with average characteristics $X_{i}$, while $\delta_{E}$ estimates the treatment effect for pupils with a PGI of 0 and average characteristics $X_{i}$. Finally, $\delta_{G\times E}$ is our estimate of interest, capturing whether the discontinuity in test scores differs by individuals’ PGI.

Table 2 suggests that our environment, *i.e.* being older in one’s grade, is as good as random. This suggests that $\delta_{E}$ is unbiased (see Table 1), capturing the causal effect of being older in one’s grade on one’s test scores. In contrast, since the specification above does not control for the parental genotypes, the PGI potentially captures a spurious correlation with the family environment. To deal with this, we include both the maternal and (imputed) paternal PGIs as additional control variables. In doing so, we additionally control for all interactions between the parental PGIs and $PGI_{i}$ and between the parental PGIs and $Treated_{i}$.^28^

In summary, our setting is rare: the environment is as good as randomly assigned and the data allow for the inclusion of the parental PGIs as covariates to account for parental genetic influences. Random $Treated_{i}$ ensures $\delta_{E}$ is unbiased and the inclusion of the parental PGIs ensures $\delta_{G}$ captures a direct (*i.e.* causal) genetic effect, removing any genetic nurture or passive *rGE* effects that enter via the parental genotype. This, in turn, implies that also $\delta_{G\times E}$ is net of any such influences and captures the causal G×E effect. Since no sufficiently well-powered parent–child or sibling GWASs exist, the coefficient on G and G×E is potentially downward biased, though they remain causal effects.

### 4.7. Results

#### Entering formal schooling:

We report the estimated main effects and their interactions for the analysis of the Entry Assessment score in Table 4. Columns (1) and (2) present the results from estimating equation (13), first without and then with the interaction term. For both specifications, we find that the treatment effect is positive and sizable (1.138 and 1.133, respectively): old-forgrade pupils (born in September–November) score on average just over one standard deviation higher on their entry test than those who are young-for-grade (born in June–August). This is consistent with the raw data in Figure 2 where treatment (here roughly the change from August to September) is about 1 standard deviation. Furthermore, our estimates suggest that among the controls, being born one month later (MoB) is associated with a 0.148 standard deviation reduction in the average test score; this trend is slightly less pronounced (though insignificantly so) for the treated (differing from 0.148 by a statistically insignificant 0.055). The PGI coefficient in column (1) suggests that a one standard deviation increase in the EA PGI is associated with an increase of 0.156 standard deviations in the Entry Assessment score—a result similar to the average predictive power of the PGI of 0.163 in Table 3.

Considering the G×E estimate (Treated × PGI Child) in Column (2), we find that the discontinuity in the Entry Assessment score by treatment status is larger for those with a higher PGI: a one standard deviation increase in the PGI is associated with an additional 0.088 standard deviation increase in the discontinuity. This is consistent with the descriptive analysis of Figure 3, showing a steeper line in the treatment versus the control group. Taken at face value, the regression results suggest that a delayed entry-age increases skill inequalities at ages 4–5 (*i.e.* before the start of formal schooling) associated with genetic endowments, although the effect is only marginally significant at the 10% level.

We next show the estimates that account for the parental PGIs as well as interactions between the parental PGIs and G and between the parental PGIs and E: our preferred specification. In line with the prediction from Table 1, compared to Column (2), the effect of the treatment remains roughly similar while the effect of the EA PGI decreases in Column (3). The results show that our main effect of interest ${\hat{\delta }}_{G\times E}$ increases both in size and in precision when including the parental PGIs and their interactions with PGI Child and Treated (0.126, s.e. 0.021, p<0.01), suggesting that the interaction effect in column (2) is unlikely to be driven by genetic nurture or passive *rGE*.

#### Progressing through formal schooling:

To explore how the G×E effect changes as children age and progress through the formal schooling system, Table 5 shows the analysis that uses the four Key Stage tests as the outcomes of interest. The main treatment effect is consistent with the previous literature: those who are older in their grade have test scores that are approximately 0.7, 0.4, 0.2, and 0.3 standard deviations higher at ages 6–7, 10–11, 13–14, and 15–16, respectively; the treated perform better than the controls on all Key Stage tests, though the difference declines as children age. The downward trend in test scores by MoB is also visible in all specifications and is less steep for those born after September. Focusing on the G×E interaction term (Treated × PGI), we find a *negative* effect that is significant across all Key Stages other than Key Stage 3, where the coefficient is close to zero. This finding suggests that although the treated have higher test scores on average, the discontinuity is *smaller* for those with a higher PGI. In other words, the benefits of delayed entry on test performance are larger for those with lower PGIs. Our preferred specification, however, is one that controls for the parental PGIs and its interactions. We find that our estimates are generally robust to their inclusion (the even columns). One exception is the KS1 assessment, for which adding these extra controls increases the standard error on the G×E interaction term substantially and prevents us from rejecting the null. However, even here the point estimate is consistent with estimates from specifications omitting these controls. In fact, adding these controls *increases* the magnitude of the estimate.

The estimated interactions, summarized in Supplementary Figure G.2, are economically meaningful. For example, for Key Stage 4, we find an average treatment effect of 0.27–0.28 standard deviations associated with being assigned to old-for-grade. The estimated interaction between treatment and the PGI suggests that two children with a one standard deviation difference in the EA PGI are expected to have differences in this treatment effect of 0.04–0.05 standard deviations, or 15–20% of the overall treatment effect. In Supplementary Appendix F, we investigate the robustness of the results with respect to non-linearities in the PGI and the chosen bandwidth of our RDD. The results remain very similar in terms of magnitude, sign, and significance. When allowing for non-linearities in addition to an extensive set of interaction terms, precision is considerably reduced—occasionally increasing *p*-values above commonly employed thresholds for statistical significance. However, even then the sign and magnitude remain very similar to our main results.

### 4.8. Interpretation and discussion

Our estimates of the treatment effect and the PGI are in line with the existing literature. We estimate that being old-for-grade has positive main effects across all available test scores: individuals assigned a later age of school entry have an educational advantage relative to their younger peers. This effect declines in magnitude for later grades, perhaps reflecting a decline in the relative age differences between treated and untreated students as they all grow older. Unsurprisingly, children with higher EA PGIs also have an advantage, and the magnitude of the main PGI effect appears to be relatively stable across grades.

The G×E interactions may seem more surprising. They are qualitatively different when considering performance on the Entry Assessment test taken before school entry (ages 4–5) compared to performance on the four Key Stage assessment tests taken as pupils progress through the schooling system (ages 7–16). Being older at school entry benefits children on the Entry Assessment test (ages 4–5), but more so for those with a higher genetic propensity for education, exacerbating genetic inequalities. By contrast, while being old-for-grade also benefits children on the assessment tests at later ages (ages 7–16), it benefits those with a lower genetic propensity for education more, reducing genetic inequalities. This qualitative difference in the G×E interaction suggests an interesting role of the formal schooling system in reducing genetic inequality. Here, we put forward an interpretation of our results given the theory outlined in Section 4.1. While speculative, this exercise highlights the potential value of incorporating economic theory into G×E studies.

The positive G×E interaction for the Entry Assessment test is in line with the broader literature on parental investment in skill formation. Children benefit both from a higher genetic propensity and from being older at school entry. This literature stresses the existence of complementarities between inputs, including between parental investments and past skills (*e.g.* Cunha and Heckman, 2007; Cunha et al., 2010; Muslimova et al., 2024). Sources of advantage tend to compound one another and magnify inequality. This makes the negative interactions for the Key Stage tests, taken after teachers start investing in children’s human capital, more surprising and of interest for public policy. Students with lower EA PGIs experience greater gains on the Key Stage tests from being old-for-grade. These opportunities for substitutability emerge within the formal schooling system, and may mitigate disparities arising from genetic factors and differences in pre-entry skills. Data on teacher-child interactions would allow us to understand the interplay within the classroom and thereby explore potential mechanisms for our findings. Unfortunately ALSPAC does not contain such data.

Nevertheless, our collection of results can still shed light on the signs and magnitudes of the mechanisms by which genotype and age at entry interact to influence later Key Stage test scores. Consider mechanism (1) from equation (11): differential pre-entry skill accumulation, formalized as $\frac{\partial \theta_{i}^{\tau }}{\partial \theta_{i}^{e}}(\theta_{i4}^{e},G_{i},4)[\frac{d\theta_{i5}^{e*}}{dG_{i}}-\frac{d\theta_{i4}^{e}}{dG_{i}}]$. Since we observe entry skills $\theta_{i}^{e}$ through the Entry Assessment, we directly evaluate this mechanism in Table 4, where we find that being old-for-grade benefits those with a high PGI more on the entry test (Treated × PGI Child is positive, 0.126 [column 3]), implying that $\frac{d\theta_{i5}^{e*}}{dG_{i}}-\frac{d\theta_{i4}^{e}}{dG_{i}}$ is positive. Assuming entry skills positively influence later skills ($\partial \theta_{i}^{\tau }∕\partial \theta_{i}^{e}>0$), and ignoring other drivers, mechanism (1) should generate a positive G×E interaction in the later Key Stage tests, $\theta_{i}^{\tau }$. The fact that we estimate a negative G×E interaction suggests that the other three mechanisms (2, 3, and 4) collectively generate a sufficiently strong negative interaction to more than offset differential pre-entry skill accumulation.

Consider mechanism (3): entry skill–gene interaction: $\frac{\partial^{2}\theta_{i}^{\tau }}{\partial G_{i}\partial \theta_{i}^{e}}(\theta_{i4}^{e},G_{i},5)[\theta_{i5}^{e*}-\theta_{i4}^{e}]$. Since being old-for-grade positively impacts entry skills, $\theta_{i5}^{e*}>\theta_{i4}^{e}$, this mechanism would be consistent with our finding if the productivity of entry skills were lower for individuals with higher PGIs, $\frac{\partial^{2}\theta_{i}^{\tau }}{\partial G_{i}\partial \theta_{i}^{e}}(\theta_{i4}^{e},G_{i},5)<0$. One (imperfect) way to evaluate this is to control for entry skills and its interaction with the PGI in our regressions. If mechanism (3) were negative, adding these controls would make the G×E interaction coefficient more positive.

We perform this exercise in Table 6 where, for each Key Stage test, we present three specifications. The first column repeats the specification found in Table 5 with our full control set (including parental PGIs) as a baseline. The second column repeats this specification, but restricts the sample to the set of children for whom we observe non-missing values of the ages 4–5 entry assessment. The third column adds a control for the Entry Assessment score, and an interaction between the Entry Assessment score and the PGI. These tests are not perfect. First, sample sizes drop substantially when restricting the sample to the subset for which we have Entry Assessment scores. More importantly, the Entry Assessment score, and its interactions with the EA PGI, are endogenous, which makes it difficult to interpret the G×E coefficient after controlling for these regressors. Comparing the second and third column in Table 6, the additional controls, if anything, make the interaction effects more negative. Moreover, we find no evidence of statistically significant interactions between entry skills and the PGI, casting doubt on mechanism (3) as an important driver of our results. Hence, while we caution the reader not to over-interpret these regressions, we consider them suggestive.

Since mechanism (1) should generate a positive interaction, and mechanism (3) does not appear to be important, we are left with mechanisms (2) and (4). Our data do not allow us to separate mechanisms (2) and (4), nor do they allow us to draw firm conclusions about how these mechanisms operate in our specific context. Nevertheless, we can offer some speculative possibilities. Since higher PGI students on average tend to have higher entry skills, ($\frac{d\theta_{i5}^{e*}}{dG_{i}}>0$), mechanism (2) could explain our results if being old-for-grade reduces the marginal productivity of entry skills in the production function in a way that disadvantages individuals with higher PGIs: $\left[\frac{\partial \theta_{i}^{\tau }}{\partial \theta_{i}^{e}}(\theta_{i5}^{e*},G_{i},5)-\frac{\partial \theta_{i}^{\tau }}{\partial \theta_{i}^{e}}(\theta_{i4}^{e},G_{i},4)\right]<0$. Higher PGI children might have higher entry skills, but this could matter less among those who are old-for-grade. On the other hand, mechanism (4), $\frac{\partial \theta_{i}^{\tau }}{\partial G_{i}}(\theta_{i4}^{e},G_{i},5)-\frac{\partial \theta_{i}^{\tau }}{\partial G_{i}}(\theta_{i4}^{e},G_{i},4)$, can explain our results if being old for grade reduces the relationship between the genetic component and skill accumulation while in school for the same level of entry skills.

Mechanisms (2) and (4) are similar because they both show how age can compensate for other factors in skill development at school, serving as a substitute for either initial skills or genetic endowments. A speculative but illustrative example could involve relational maturity—older children could be more mature or confident in ways that allow them to better ask for help, or extract assistance from teachers or peers. Among old-for-grade children, this might offset advantages that students have from genetic endowments in formal schooling. Children with low PGIs who are old-for-grade may thus be able to more easily catch-up with their peers if they start at a skills deficit. Another possibility is that teachers may target their time and attention in the classroom on those who are falling behind. Indeed, a potentially important feature of the UK institutional education setting is the fact that children do not repeat school years. Hence, to ensure that all children reach the minimum required standard to progress to the next year, teachers will spend more time with lower-performing students. Since these are more likely to be August-borns and low-PGI pupils (who on average perform worse on educational tests), our negative G×E effects are consistent with the existence of complementarities between genetic endowments and teacher inputs. This also means that our findings may be specific to our setting; it will be interesting to explore whether they replicate in other educational systems.

Whatever the specific micro-foundations, it is noteworthy that these opportunities for substitutability emerge within the formal schooling system. Under the right conditions (*e.g.* when children are older at entry), the instruction and personal interactions provided by the formal schooling system may work to mitigate disparities arising from genetic factors and differences in pre-entry skills. Such possibilities may not be obvious from the existing literature, which stresses complementarities between endowments and early, pre-schooling skills. Our findings are, however, consistent with those of Arold et al. (2022) and Cheesman et al. (2022), who find that children with lower EA PGIs benefit more from high-quality teachers and schools. Taken together, these findings provide a fruitful area for future theoretical and empirical research on differential interactions between endowments and investments in the home versus school setting.

Depending on policy preferences regarding equity and efficiency, our finding that children with lower PGIs appear to gain the most from delaying entry into formal schooling provides important information to set school entry age policies and determine appropriate public funding for preschools. More speculatively, one could also think in terms of the targeting of interventions or school policies. In many school systems, families have some control over when their children enter the formal schooling system. Indeed, if the ALSPAC cohort were starting school now, families with children born between April 1 and August 31 could decide to hold them back a year. In such settings, individual families on the margin might use privately acquired genetic test results to help guide the decision about when to have a child enter formal schooling. It is certainly not clear that the magnitudes of the interactions we find would make genetic information useful for this purpose, particularly given the current limitations on using genetic data for *individual*-level prediction of outcomes such as EA (Morris et al., 2020b). However, whether a particular effect size is large depends on the preferences, risk-tolerances, and expectations of individual families.

## 5. CONCLUSION

Recent advances in the collection and processing of genetic data have created new opportunities for improving our understanding of how nature and nurture interact in shaping individual outcomes, illuminating some of the oldest questions in the social sciences from a new angle. Economists can benefit from these advances, even if they are not interested in genetic effects. This is because G×E analyses can (i) assess treatment effect heterogeneity, (ii) test theoretical predictions, and (iii) uncover economic, social and behavioural mechanisms. We demonstrated best practice in a study of G×E interplay, examining interactions between one’s age at school entry and one’s genetic propensity for EA. Our results suggest (previously unobserved) differential productivity by child genotype, where children with a lower genetic propensity for education benefit more in terms of their test scores from delayed school entry.

Our empirical application is one of few studies (see Supplementary Table G.1 in Appendix G) exploiting random variation in both genotype and environment, enabling causal inferences in both G and E. While this is an important contribution on its own, our extensive methodological review also illustrates the power of interdisciplinary research. Combining approaches from genetics with its focus on the estimation of causal genetic effects, and economics with its focus on causal environmental effects, allows for what we consider the “ideal experiment”. In this ideal experiment, randomized variation in genotype (ideally based on a within-family GWAS) is combined with quasi-random variation in the environment.

Governments are investing heavily in large biobanks for research. The UK Biobank (~500,000 genotyped individuals) has been available for some years, and next-generation biobanks such as the All of US in the U.S. and Our Future Health in the U.K. are ambitiously building biobanks of, respectively, 1 and 5 million individuals. These datasets will alter the playing field of future GWASs, increasing the number of outcomes for which genetic data explains a substantial part of its variation. These data collection efforts will also increase the availability of genetic data from relatives, allowing for well-powered within-family GWASs. This would pave the way for the “ideal experiment”.

There are ethical issues involved in working with genetic data and researchers have obligations to preserve the highest standards of privacy, confidentiality, and responsible communication. Researchers must also take seriously the need to help the public understand how to interpret research findings based on genetic data and to clarify what conclusions can and cannot be drawn (Nature, 2013). We would argue that the potential uses of genetic data carry societal implications and associated risks, but simply denying the existence of genetic differences across individuals is unlikely to be the right solution (Raffington et al., 2020; Harden, 2021b). Research in economics and the social sciences on G×E interplay can help identify causal pathways involved in individual development and refute genetic or environmental determinism while identifying policy-relevant environments that can reduce socioeconomic or genetic inequalities. In doing so, it may help improve the well-being of the population.

## Supplementary Material

Supplementary Appendices A to G

Supplementary data are available at *Review of Economic Studies* online.

## Figures and Tables

**TABLE 1:** Estimation scenarios for G×E effects in gene–environment interaction models

	Exogenous E	Endogenous E	
		Predetermined E	Non-predetermined E
Exogenous G (parent–child/sibling data) & PGI on basis of parent–child/sibling GWAS	✓G unbiased (causal)	✓G unbiased (causal)	✓G unbiased (causal)
	✓E unbiased (causal)	↑↓ E may reflect (predetermined) $E^{*}$ through correlated environments	↑↓ E may reflect $E^{*}$ through correlated environments or G through active/evocative *rGE*
Exogenous G (parent–child/sibling data) & PGI on the basis of regular GWAS	↓ G downward biased (within-family measurement error)	↓ G downward biased (within-family measurement error)	↓ G downward biased (within-family measurement error)
	✓ E unbiased (causal)	↑↓ E may reflect (predetermined) $E^{*}$ through correlated environments	↑↓ E may reflect $E^{*}$ through correlated environments or G through active/evocative *rGE*
	Ex. Muslimova et al. (2024, Birth order; UK Biobank)	Ex. Houmark et al. (2022, Family circumstances; iPSYCH)	Ex. Cheesman et al. (2022, Social context; MoBa)
Endogenous G (between family data) & PGI on the basis of regular GWAS	↑ G upward biased; may reflect $E^{*}$ or parental G	↑ G upward biased; may reflect (predetermined) $E^{*}$	↑ G upward biased; may reflect E, $E^{*}$ or parental G
	✓ E unbiased (causal)	or parental G	↑↓ E may reflect $E^{*}$ or parental G, or G through active/evocative *rGE*
		↑↓ E may reflect (predetermined) $E^{*}$ or parental G	
	Ex. Schmitz and Conley (2017b, Vietnam draft; HRS)	Ex. Papageorge and Thom (2020, Family circumstances; HRS)	Ex. Arold et al. (2022, Teacher quality; AddHealth)

*Notes:* The bias discussed in the nine estimation scenarios focus on the G×E analysis (rather than the GWAS discovery) stage. G stands for genotype, E for environment, $E^{*}$ for environments *other than* those of interest, and *rGE* for gene–environment correlation. A predetermined environment E is defined as an environment not causally influenced by one’s genes G yet possibly correlated with other environmental characteristics $E^{*}$ and potentially shaped by parental genes. In addition to the sources of bias presented in the table, any classical measurement error will lead to attenuation bias. Dataset acronyms (*e.g.* HRS) are spelled out in Supplementary Appendix D, where we discuss the six representative examples from the literature cited in the current table.

**TABLE 2:** Descriptive statistics of child and family characteristics by treatment status

	Treated		Control		*t*-test
	*N*	Mean	*N*	Mean	*p*-value
Mother’s age at first pregnancy (years)	2,062	25.138	2,168	25.257	0.431
Mother smoked cigarettes during pregnancy^a^	1,927	0.167	2,052	0.168	0.896
Mother’s anxiety score during pregnancy	1,888	4.651	2,037	4.659	0.946
Mother’s depression score during pregnancy	1,887	4.245	2,038	4.211	0.714
Mother’s marital status^a^	2,061	0.843	2,169	0.859	0.153
Mother has vocational training^a^	2,047	0.096	2,146	0.088	0.361
Mother has O-levels^a^	2,047	0.338	2,146	0.366	0.056
Mother has A-levels^a^	2,047	0.248	2,146	0.241	0.609
Mother has a university degree^a^	2,047	0.157	2,146	0.156	0.882
Father has vocational training^a^	1,976	0.082	2,085	0.071	0.188
Father has O-levels^a^	1,976	0.197	2,085	0.218	0.101
Father has A-levels^a^	1,976	0.275	2,085	0.278	0.836
Father has a university degree^a^	1,976	0.214	2,085	0.228	0.258
Mother in Social Class II^a^	1,713	0.325	1,864	0.326	0.948
Mother in Social Class III (non-manual)^a^	1,713	0.422	1,864	0.434	0.471
Mother in Social Class III (manual)^a^	1,713	0.067	1,864	0.072	0.576
Mother in Social Class IV^a^	1,713	0.101	1,864	0.083	0.058
Mother in Social Class V^a^	1,713	0.016	1,864	0.014	0.652
Child’s birthweight (kg)	2,089	3.448	2,189	3.451	0.875
PGI Child	2,114	0.016	2,209	0.037	0.499
PGI Mother	1,526	0.036	1,533	0.022	0.688
PGI Father	1,478	0.006	1,475	0.039	0.367

*Notes:* Sample size and means for a set of child and family characteristics observed before or at birth.

a A binary variable (0 = No;1 = Yes). Mother’s anxiety and depression scores are sub-scores of the Crown-Crisp Experimental Index, capturing maternal mental health during the pregnancy period. Higher scores mean the mother is more affected. Mother’s marital status is equal to one for those ever married (including those widowed, divorced, or separated), and zero otherwise. Maternal and paternal education is defined as vocational training, ordinary (O) level, advanced (A) level, and university degree. Social class is defined using the standard U.K. classification of class based on occupation: professional (I), managerial and technical (II), non-manual skilled (IIInm), manual skilled (IIIm), semi-skilled (IV), and unskilled (V). The last column shows the *p*-value from a *t*-test of the difference in means between the treated and control group.

**TABLE 3:** OLS (Ordinary Least Squares) estimates of the effect of the PGIs for EA on test scores at different ages

	Entry Assessment Ages 4–5	Key Stage 1 Ages 6–7	Key Stage 2 Ages 10–11	Key Stage 3 Ages 13–14	Key Stage 4 Ages 15–16
*Panel A: No parental PGI controls*					
PGI Child	0.163*** (0.028)	0.255*** (0.015)	0.344*** (0.015)	0.357*** (0.017)	0.318*** (0.016)
*R* ^2^	0.085	0.096	0.128	0.134	0.128
Observations	1,094	3,436	3,610	3,073	3,579
*Panel B: Parental PGI controls*					
PGI Child	0.163*** (0.039)	0.248*** (0.022)	0.321*** (0.022)	0.301*** (0.024)	0.302*** (0.023)
PGI Mother	0.032 (0.039)	0.081*** (0.021)	0.069*** (0.020)	0.108*** (0.023)	0.055*** (0.021)
PGI Father	−0.025 (0.042)	−0.059^**^ (0.024)	−0.016 (0.023)	0.017 (0.026)	−0.015 (0.024)
*R* ^2^	0.086	0.111	0.135	0.146	0.131
Observations	1,094	3,436	3,610	3,073	3,579

*Notes:* The test score and the PGI for EA are standardized to have mean 0 and standard deviation 1 in the analysis sample. All regressions control for gender and the first ten principal components of the genetic data, as well as a dummy if the parental PGIs are missing. Robust standard errors in parentheses. *p < 0.10, ** p < 0.05, ***p < 0.01.

**TABLE 4:** OLS estimates of the main and interaction effects of being old-for-grade (Treated) and the EA PGI on children’s Entry Assessment (ages 4–5) test score, with and without controls for parental PGIs

	(1)	(2)	(3)
Treated	1.138^***^ (0.088)	1.133^***^ (0.077)	1.151^***^ (0.077)
PGI Child	0.156^***^ (0.027)	0.024 (0.024)	−0.049 (0.025)
Treated × PGI Child		0.088^*^ (0.035)	0.126^***^ (0.021)
MoB	−0.148^**^ (0.042)	−0.150^**^ (0.039)	−0.156^***^ (0.038)
Treated × MoB	0.055 (0.045)	0.059 (0.045)	0.064 (0.041)
MoB × PGI Child		−0.080^**^ (0.021)	−0.088^***^ (0.021)
MoB × PGI Child × Treated		0.127^***^ (0.025)	0.138^***^ (0.026)
PGI Mother			0.016 (0.051)
PGI Father			0.114^**^ (0.038)
PGI Mother × Treated			0.075 (0.060)
PGI Father × Treated			−0.131^**^ (0.034)
PGI Mother × PGI Child			0.023 (0.042)
PGI Father × PGI Child			−0.018 (0.016)
*R* ^2^	0.258	0.267	0.278
Observations	1,094	1,094	1,094

*Notes:* The analysis uses a bandwidth of 3 months before and after the September cut-off (*i.e.* June till November). Additional control variables include gender, year of birth, the first ten principal components, a dummy for missing parental PGI, and interactions of all covariates with PGI Child and with treated. Robust standard errors in parentheses, clustered by MoB. *p < 0.10, **p < 0.05, ***p < 0.01.

**TABLE 5:** OLS estimates of the main and interaction effects of being old-for-grade (Treated) and the EA PGI on children’s key stage test scores

	KS1 (ages 6–7)		KS2 (ages 10–11)		KS3 (ages 13–14)		KS4 (ages 15–16)	
	(1)	(2)	(3)	(4)	(5)	(6)	(7)	(8)
Treated	0.698^***^ (0.026)	0.687^***^ (0.025)	0.389^***^ (0.021)	0.379^***^ (0.021)	0.223^**^ (0.057)	0.210^**^ (0.056)	0.281^***^ (0.021)	0.274^***^ (0.021)
PGI Child	0.316^***^ (0.002)	0.303^***^ (0.034)	0.368^***^ (0.020)	0.319^***^ (0.015)	0.321^***^ (0.016)	0.211^***^ (0.014)	0.348^***^ (0.004)	0.297^***^ (0.018)
Treated × PGI Child	−0.088^**^ (0.026)	−0.107 (0.059)	−0.052^*^ (0.023)	−0.093^***^ (0.021)	0.011 (0.022)	0.008 (0.039)	−0.050^***^ (0.010)	−0.042^*^ (0.019)
MoB	−0.083^***^ (0.015)	−0.082^***^ (0.014)	−0.096^***^ (0.009)	−0.094^***^ (0.009)	−0.023 (0.014)	−0.022 (0.014)	−0.036^**^ (0.010)	−0.035^**^ (0.010)
Treated × MoB	0.049^**^ (0.013)	0.048^**^ (0.013)	0.080^***^ (0.011)	0.079^***^ (0.011)	0.018 (0.032)	0.020 (0.031)	0.019 (0.012)	0.020 (0.012)
MoB × PGI Child	0.040^***^ (0.002)	0.040^***^ (0.003)	0.011 (0.013)	0.014 (0.013)	−0.013 (0.012)	−0.012 (0.012)	0.020^***^ (0.003)	0.022^***^ (0.004)
MoB × PGI Child×Treated	−0.012 (0.012)	−0.014 (0.013)	0.015 (0.012)	0.014 (0.012)	0.048^***^ (0.011)	0.047^**^ (0.013)	−0.002 (0.010)	−0.002 (0.008)
PGI Mother		0.093^*^ (0.036)		0.086^***^ (0.010)		0.154^***^ (0.021)		0.076^***^ (0.005)
PGI Father		−0.051 (0.036)		0.028 (0.026)		0.084^*^ (0.033)		0.035 (0.023)
PGI Mother × Treated		−0.007 (0.054)		0.025^*^ (0.012)		−0.025 (0.035)		−0.009 (0.031)
PGI Father × Treated		0.038 (0.041)		0.033 (0.027)		0.023 (0.053)		−0.013 (0.033)
PGI Mother × PGI Child		−0.009 (0.020)		0.002 (0.019)		0.020 (0.020)		0.002 (0.014)
PGI Father × PGI Child		0.007 (0.015)		0.000 (0.013)		0.002 (0.014)		−0.007 (0.012)
*R* ^2^	0.182	0.195	0.151	0.162	0.149	0.164	0.145	0.149
Observations	3,436	3,436	3,610	3,610	3,073	3,073	3,579	3,579

*Notes:* The analysis uses a bandwidth of 3 months before and after the September cut-off (*i.e.* June till November). Additional control variables include gender, year of birth, the first ten principal components, a dummy for missing parental PGI, and interactions of all covariates with PGI Child and with Treated. Robust standard errors in parentheses, clustered by MoB. *p < 0.10, **p < 0.05, ***p < 0.01.

**TABLE 6:** OLS estimates of the main and interaction effects of being old-for-grade (Treated) and the EA PGI on children’s Key Stage test scores, controlling for Entry Assessment

	KS1 (age 7)			KS2 (age 11)			KS3 (age 14)			KS4 (age 16)		
	(1)	(2)	(3)	(4)	(5)	(6)	(7)	(8)	(9)	(10)	(11)	(12)
Treated	0.687^***^ (0.025)	0.697^***^ (0.055)	0.103 (0.059)	0.379^***^ (0.021)	0.237^*^ (0.096)	−0.318^**^ (0.104)	0.210^**^ (0.056)	0.120 (0.081)	−0.320^***^ (0.064)	0.274^***^ (0.021)	0.180^***^ (0.021)	−0.279^***^ (0.047)
PGI Child	0.303^***^ (0.034)	0.330^***^ (0.024)	0.322^***^ (0.018)	0.319^***^ (0.015)	0.298^***^ (0.061)	0.319^***^ (0.033)	0.211^***^ (0.014)	0.180^**^ (0.057)	0.200^***^ (0.035)	0.297^***^ (0.018)	0.251^***^ (0.033)	0.271^***^ (0.027)
Treated × PGI Child	−0.107 (0.059)	−0.039 (0.073)	−0.093 (0.061)	−0.093^***^ (0.021)	−0.021 (0.088)	−0.131^*^ (0.054)	0.008 (0.039)	−0.006 (0.126)	−0.085 (0.128)	−0.042^*^ (0.019)	−0.091 (0.073)	−0.179^*^ (0.070)
Entry Assessment (ages 4–5)			0.545^***^ (0.037)			0.505^***^ (0.033)			0.402^***^ (0.018)			0.405^***^ (0.039)
Ent. Ass. × PGI Child			0.010 (0.036)			0.030 (0.024)			0.038 (0.043)			0.045 (0.026)
*R* ^2^	0.195	0.221	0.441	0.162	0.171	0.344	0.164	0.177	0.284	0.149	0.167	0.282
Observations	3,436	959	959	3,610	956	956	3,073	865	865	3,579	998	998

*Notes:* The analysis uses a bandwidth of 3 months before and after the September cut-off (*i.e.* June till November). The models include parental PGIs. Additional control variables include gender, year of birth, the first ten principal components, a dummy for missing parental PGI, and interactions of all covariates with PGI Child and with Treated. Robust standard errors in parentheses, clustered by MoB. *p < 0.10, **p < 0.05, ***p < 0.01. The first column in each set of results reproduces the final specification in Table 5; the second column includes the same specification but in the sample with non-missing Entry Assessment (ages 4–5) scores; the third column controls for Entry Assessment and its interaction with PGI Child.

## Data Availability

The code to reproduce this article is available on Zenodo at https://doi.org/10.5281/zenodo.14968164. The data used in this study is the ALSPAC. Due to confidentiality agreements, access to ALSPAC data must be requested directly from the data providers (University of Bristol). Details on data access can be found at: https://www.bristol.ac.uk/alspac/.

## Footnotes

1. Gene–environment interplay differs from the study of epigenetics, which focuses on the role of gene *expression*. Whereas individuals’ genotype is fixed at conception, their gene expression may change over the life course due to environmental influences. As such, epigenetic processes can provide one explanation for the existence of gene– environment interplay, but they are not the only possible mechanism. See Supplementary Appendix B.4 for more details.

2. Different terms are used to refer to the genetic component, including genotype, genetic makeup, genetic propensity, genetic endowments, genetic variants, polygenic scores, and PGI. The Glossary in Supplementary Appendix A provides definitions of the most important genetic terminology used in the paper.

3. Examples include Benjamin et al. (2012a, 2024) and Beauchamp et al. (2011), and more specifically the surveys on G×E interplay, as in Fletcher and Conley (2013) and Schmitz and Conley (2017a). See Rutter (2006), Plomin (2014), Mills et al. (2020), and Domingue et al. (2020) for related work in other disciplines.

4. Assortative mating based on EA and correlated characteristics has been shown to induce a non-negligible correlation in genotypes across mothers and fathers in the order of 0.10–0.20 (*e.g.* Robinson et al., 2017; Morris et al., 2020a; Torvik et al., 2022; Collado et al., 2023).

5. Passive *rGE* also occurs when siblings’ genotypes partially shape the environment that individuals are exposed to (*e.g.* Cawley et al., 2019). Since siblings share, on average, 50% of their DNA, this introduces a correlation between sibling genotypes and sibling environments. However, conditional on parental genotypes, the child’s genotype is as good as random, fully eliminating any confounding due to passive *rGE* (as we discuss later).

6. Other definitions, *e.g.* Young et al. (2022), also include indirect genetic effects stemming from passive *rGE* as part of the causal effect of G. From a dynastic point of view, these definitions are similar—hypothetically, a change in one’s genotype at conception will have implications for individuals and their offspring (*i.e.* may lead to passive *rGE* for the next generation). We here take an individual’s genotype as the relevant unit of analysis and therefore treat passive *rGE* arising from relatives’ genotypes as a source of bias rather than a causal effect.

7. This property is also exploited in so-called Mendelian randomization studies that use genotypes as instrumental variables for endogenous environmental exposures (Von Hinke et al., 2016). Mendel’s law also allows for calculating the expected relatedness among offspring given the relatedness of the parents. Deviations from expected relatedness (“relatedness disequilibrium”) are due to random segregation and can, therefore, be used to estimate a trait’s heritability free from environmental bias (Young et al., 2018)

8. Assortative mating on EA induces a correlation across SNPs that influence the genetic propensity to EA in future generations. It can be thought of as a form of population stratification (see also Supplementary Appendix B.2), where children from higher educated parents inherit different alleles compared with children from lower educated parents (Morris et al., 2020a). Because of environmental transmission of the propensity to higher education, the importance of particular SNPs that are over-represented in higher-educated families will be overestimated, biasing the predictive power of a PGI upwards (*e.g.* Okbay et al., 2022).

9. A fourth category exists where a researcher has access to a PGI based on the results of a parent–child or sibling GWAS but applies this in a sample without parent–child/sibling pairs. There are currently no such studies, and therefore we do not separately discuss it since the issues highlighted in scenario (3) also apply here (see Supplementary Appendix C).

10. Since the only confounding variables in a GWAS are the father’s and mother’s genotypes, the GWAS is sometimes also performed using a regression without family fixed effects but with the mean sibling’s genotype as a control variable (*e.g.* Howe et al., 2022). The inclusion of the mean sibling’s genotype is sufficient to control for the mean influence of parental genotype and therefore leads to point estimates equivalent to those from equation (3).

11. However, if parents respond differently to their children’s genetic endowments (see, *e.g.* Sanz-de-Galdeano and Terskaya, 2023), genetic nurture may differ between siblings. Moreover, in theory, it could be that the interaction between the direct genetic effect and a certain environment has an opposite sign compared to the interaction between genetic nurture and the environment, such that the G×E term is not necessarily attenuated.

12. This is not the case for a predetermined E, since the environment precedes G, implying that E is not the result of G. A predetermined E, therefore, rules out active and evocative *rGE* but not passive *rGE* (hence our distinction between exogenous, predetermined, and non-predetermined E).

13. For example, our application investigates how being old-for-grade, captured by being born in September versus August, interacts with one’s EA PGI in explaining test performance. If the original GWAS was performed only on September-borns (an unlikely scenario but useful for illustration), the PGI would likely be more predictive for this group than for August-borns since it better captures the environments experienced by September-born students (*e.g.* longer exposure to the parental rearing environment, being older than one’s peers, etc.). A regression of the outcome of interest on G, E, and G×E may then lead to a positive estimate on the interaction, merely from picking up the additional predictive power among those with September birthdays without an interaction effect necessarily existing.

14. In cases with non-systematic or non-linear environmental moderators (*e.g.* E increases the effect size of one SNP but decreases another), the interaction effect PGI × E may be zero. In other words, a null PGI × E coefficient does not preclude other types of G×E explaining part of the variation in an outcome (see Miao et al., 2022, for more details and an empirical test for this case). An alternative specification to explore G×E is a so-called genome-wide *interaction* study (GWIS), which can be used to construct PGIs for the main and interaction effects (Jayasinghe et al., 2024). However, such GWIS results are not available in sufficiently large samples yet, in particular not for the (exogenous) environments E that economists are typically interested in.

15. Throughout our empirical analysis, we follow best practice for G×E research as described above. For the applied researcher, we outline the empirical analytical steps in Supplementary Appendix E.1 and provide all syntax in our GitHub or Zenodo repository. We also conducted ex-ante power calculations, reported in Supplementary Appendix E.2. These analytical steps can be broadly applied to similar analyses.

16. We assume a monotonic relationship between the genetic endowment $G_{i}$, measured by the PGI, and the skill endowment $\theta_{i}^{e}$. This is consistent with descriptive analyses that show a monotonic relationship between the PGI and human capital outcomes like EA.

17. In particular, modelling how parents allocate investments across children would be particularly valuable for interpreting G×E results emerging from within-family designs that make use of sibling-level variation in PGIs.

18. $\theta_{i}^{\tau }(\theta_{i5}^{e*},G_{i},5)\approx \theta_{i}^{\tau }(\theta_{i4}^{e},G_{i},5)+\frac{\partial \theta_{i}^{\tau }}{\partial \theta_{i}^{e}}(\theta_{i4}^{e},G_{i},5)(\theta_{i5}^{e*}-\theta_{i4}^{e})$.

19. Add and subtract $\frac{\partial \theta_{i}^{\tau }}{\partial \theta_{i}^{e}}(\theta_{i4}^{e},G_{i},4)\frac{d\theta_{i5}^{e*}}{dG_{i}}$ and rearrange to get:

$$\frac{\partial \Delta_{i}^{\theta }}{\partial G_{i}}=\frac{\partial \theta_{i}^{\tau }}{\partial \theta_{i}^{e}}(\theta_{i4}^{e},G_{i},4)\left[\frac{d\theta_{i5}^{e*}}{dG_{i}}-\frac{d\theta_{i4}^{e}}{dG_{i}}\right]+\left[\frac{\partial \theta_{i}^{\tau }}{\partial \theta_{i}^{e}}(\theta_{i5}^{e*},G_{i},5)-\frac{\partial \theta_{i}^{\tau }}{\partial \theta_{i}^{e}}(\theta_{i4}^{e},G_{i},4)\right]\frac{d\theta_{i5}^{e*}}{dG_{i}}\;\;+\frac{\partial \theta_{i}^{\tau }}{\partial G_{i}}(\theta_{i5}^{e*},G_{i},5)-\frac{\partial \theta_{i}^{\tau }}{\partial G_{i}}(\theta_{i4}^{e},G_{i},4)\;.$$

The last two terms $\frac{\partial \theta_{i}^{\tau }}{\partial G_{i}}(\theta_{i5}^{e*},G_{i},5)$ and $\frac{\partial \theta_{i}^{\tau }}{\partial G_{i}}(\theta_{i4}^{e},G_{i},4)$ still differ in two of their arguments, namely skill and age at school entry, whereas the other terms only in one. To better highlight the differences at play here, take the linear approximation of $\frac{\partial \theta_{i}^{\tau }}{\partial G_{i}}(\theta_{i5}^{e*},G_{i},5)$ around the entry skill level, $\theta_{i}^{e}=\theta_{i4}^{e}$, and evaluate at $\theta_{i5}^{e*}$, *i.e.*. $\frac{\partial \theta_{i}^{\tau }}{\partial G_{i}}(\theta_{i5}^{e*},G_{i},5)\approx \frac{\partial \theta_{i}^{\tau }}{\partial G_{i}}(\theta_{i4}^{e},G_{i},5)+\frac{\partial^{2}\theta_{i}^{\tau }}{\partial G_{i}\partial \theta_{i}^{e}}(\theta_{i4}^{e},G_{i},5)[\theta_{i5}^{e*}-\theta_{i4}^{e}]$. Inserting this expression leads to equation (11).

20. The sample size for analyses using data collected after age 7 is 15,454 pregnancies (15,589 foetuses), with 14,901 children alive at age 1. For more information on ALSPAC, see Boyd et al. (2013) and Fraser et al. (2013). Details of all the available data can be found through a fully searchable data dictionary and variable search tool.

21. Although parents can now choose to send their child to school a year earlier or hold them back a year, this was not possible for the pupils in our dataset. Note also that children do not repeat school years in England.

22. LDpred adjusts the GWAS weights for LD using a Bayesian approach. We re-weight the SNP effects on the basis of LD and the supposed fraction of causal SNPs, which we set to 1, as is standard practice for behavioural traits (Cesarini and Visscher, 2017).

23. At age 18, study children were sent “fair processing” materials describing ALSPAC’s intended use of their health and administrative records and were given clear means to consent or object via a written form. Data were not extracted for participants who objected, or who were not sent fair processing materials.

24. For KS1, this is an average of the child’s reading, writing, spelling, and mathematics scores; KS2 includes reading, writing, science, and mathematics tests. For KS3 and KS4, the final score is an average of the child’s English, mathematics and science scores.

25. The three-month bandwidth balances the need for efficiency (including many data points) and the potential of bias (staying close to the cut-off). Our results are robust to different bandwidths (seeSupplementary Appendix F).

26. When we only include the parental PGIs, omitting the child PGI, both the maternal and paternal PGI are highly statistically significant and often of relatively similar magnitude (see Supplementary Table G.2 in Appendix G).

27. $\delta_{5}(X_{i},PGI_{i},Treated_{i})$ denotes all interactions between the demeaned covariates (*i.e.* gender and year of birth) and $PGI_{i}$, as well as between the demeaned covariates and $Treated_{i}$; $\delta_{6}(\sum_{p=1}^{10}PC_{i}^{p},PGI_{i},Treated_{i})$ is shorthand notation for all interactions between the ten demeaned principal components and $PGI_{i}$ and between the ten demeaned principal components and $Treated_{i}$.

28. To ensure we keep the same sample as that in equation (13) (without parental PGIs), we use mean imputation if the parental PGIs are missing and include a dummy to indicate these cases
