# Supplementary material for: The Economics and Econometrics of Gene–Environment Interplay
Source: Rev Econ Stud. Author manuscript; Available in PMC 2026 Apr 22. (PMC13098532; doi:10.1093/restud/rdaf034)
Supplement: Supplementary Appendices A to G [file NIHMS2160678-supplement-Supplementary_Appendices_A_to_G.pdf]

A Glossary

In this section, we provide an overview of the genetic terms and concepts used in the paper.

Active gene–environment correlation: An association between genetic variation and an environment resulting from self-selection of genetically different individuals into particular environments.

Alleles: The nucleotides that can be present at a specific location in DNA.

Base pairs: Nucleotides are paired: “A” on one strand of the DNA always binds with “T” on the other strand, and “C” always binds with “G”. These combinations are called base pairs.

Candidate gene: A polymorphism hypothesized to be associated with a particular phenotype.

Chromosome: A long DNA molecule. Every cell in the human body contains 23 pairs of chromosomes (22 so-called autosomal chromosomes and 1 sex chromosome). One of each pair is inherited from the mother and the other from the father.

Copy Number Variant (CNV): A type of genetic variation that refers to the duplication or deletion of a specific segment of DNA in an individual’s genome. These variations can range in size from a few base pairs to large stretches of DNA encompassing multiple genes.

DNA: Human deoxyribonucleic acid (DNA), the sequence of about 3 billion pairs of nucleotide molecules. Its double-helix structure joins two strands of DNA, where the nucleotide “A” binds with “T”, and “G” binds with “C”.

Evocative gene–environment correlation: An association between genetic variation and an environment resulting from an environmental reaction to genetic differences.

Epigenetics: The study of heritable phenotypic variation that does not involve changes in the DNA sequence of nucleotides.

Gene–environment correlation: An association between genetic variation and an environment.

Gene: A sequence of nucleotides in the DNA that encodes for a particular protein or proteins.

Genetic nurture: Parental genes influencing offspring outcomes through environmental pathways.

Genetic variation: Differences in the DNA among individuals; copy-number variants (CNV) and single-nucleotide polymorphisms (SNPs) constitute the most common source of genetic variation.

Genotype: The complete set of genetic material. It can, however, also refer to the specific combination of base pairs (in a chromosome pair) at a particular location in the DNA sequence. If the base pairs are the same, the genotype is homozygous. If they are different, it is heterozygous.

Genome-wide association study (GWAS): A study in which millions of polymorphisms from the whole genome are individually tested for association with a phenotype.

GWAS meta-analysis: Meta-analysis of genome-wide association study (GWAS) results from different samples.

Genome-wide significance: The significance level at which an association is considered statistically significant in a genome-wide association study (GWAS) (5×10^{-8}).

G$\times$E interplay: The interplay between people’s genotype and the (e.g., social, biological, and economic) environment in which they live contributing to intra-individual differences.

Heritability: The proportion of the total variance in a phenotype that can be explained by genetic factors.

Imputation: Imputation of not directly genotyped genetic variation from reference panels based on linkage disequilibrium (LD).

Linkage disequilibrium (LD): The correlation between adjacent nucleotides in the DNA resulting from the co-inheritance of alleles.

Locus: A stretch of nucleotides in strong linkage disequilibrium (LD) with each other.

Major allele: The allele of a single-nucleotide polymorphism (SNP) that is most common in the population.

Manhattan plot: A plot often used to visualize genome-wide association study (GWAS) results, with genomic coordinates on the x axis and the negative logarithm of the associated p value for each SNP on the y axis. An example is Figure B.1.

Minor allele: The allele of a single-nucleotide polymorphism (SNP) that is least common in the population.

Nucleotide: The basic component molecules of DNA. Human DNA is composed of a sequence of about 3 billion pairs of nucleotide molecules. There are four different nucleotides in the DNA: adenine (A), guanine (G), cytosine (C) and thymine (T).

Passive gene–environment correlation: An association between people's genetic makeup and an environment resulting from the correlation between parental genes and the environment in which the child is raised.

Phenotype: An observable trait of an organism.

Pleiotropy: One gene/genetic variant influencing more than one phenotype.

Polygenic index: The best linear genetic predictor of a phenotype, constructed as the linear combination of single-nucleotide polymorphisms (SNPs) weighted by their association with the phenotype as estimated in a genome-wide association study (GWAS). It also also referred to as polygenic score.

Polygenic trait: A trait influenced by many genetic variants, with each having a small effect.

Polymorphism: Locations in the DNA where the nucleotides differ between individuals.

Population stratification: The presence of a systematic difference in allele frequencies between subpopulations within a population.

Principal components: Principal components extracted from the genetic relatedness matrix; can be used to control for subtle population stratification.

Single-nucleotide polymorphism (SNP): A single nucleotide location in the DNA that varies between individuals.

B Primer on genetics

B.1 Genetics in a nutshell

Human DNA is composed of sequences of approximately 3 billion pairs of nucleotide molecules. These nucleotides come in four varieties: adenine (A), guanine (G), cytosine (C) and thymine (T). The nucleotides together constitute the genome. The human genome is divided into 23 pairs of chromosomes (22 autosomal chromosomes and 1 sex chromosome), where for each pair, one chromosome is inherited from the mother and one from the father. Each chromosome contains a single double-stranded piece of DNA. "A" on one strand is always paired with "T" on the other strand, and "C" is always paired with "G". These combinations are called base pairs, and stretches of these pairs coding for a protein are called genes. The human genome consists of around 25,000 genes that code for proteins with a specific function (International Human Genome Sequencing Consortium, 2004). In addition, there are regions in-between genes with important regulatory functions.

Most nucleotides (~99.9%) in human DNA are identical from person to person. The part of DNA where people are different from each other are called polymorphisms; the location of most polymorphisms is well known by now. For many applications, it is therefore not necessary to sequence each individual's full genome. The most common polymorphisms are Copy Number Variants (CNVs)—genetic variations involving duplications or deletions of DNA segments—and Single Nucleotide Polymorphisms (SNP)— one-letter variations at a single-nucleotide locus. These variants of nucleotides are called alleles. In the human genome, there are approximately 85 million SNPs with a minor allele (i.e., the less common allele) frequency of $> 1\%$ (The 1000 Genomes Project Consortium, 2015). Current genotyping arrays measure several million of these SNPs, and many more that are not measured (typically >40 million) can be imputed with high accuracy because of the correlation structure in the genome (so-called linkage disequilibrium, Reich et al., 2001, further explained below) and the availability of large reference panels (Quick et al., 2020).

B.2 Genome-wide association studies

The polygenic nature of most traits was established through genome-wide association studies (GWASs). In a GWAS, one tests for associations between J genetic variants (SNPs) and an outcome of interest without restricting the set of SNPs on theoretical grounds. Specifically, an ideal GWAS relates all SNPs (G_{ij}, coded as 0, 1, or 2, reflecting the number of minor alleles) to a specific outcome (Y_i) for individual i in a regression framework of the form

$$Y_i = \sum_{j=1}^{J} \beta_j G_{ij} + \mathbf{x}_i^{'} \zeta + \varepsilon_i, \tag{B.1}$$

with SNP effects β_j, relevant controls $\mathbf{x}_i$, and an error term ε_i. In practice, however, this ideal model cannot be identified since existing datasets cover fewer individuals than SNPs (Benjamin et al., 2012).[29] GWASs therefore consider all J SNPs by running sequential regressions for each SNP at a time. Thus, in its most basic form, a GWAS regresses the outcome of interest on a single SNP j and repeats this procedure J times, once for each SNP:

$$Y_i = \beta_j^{GWAS} G_{ij} + \mathbf{x}_i^{'} \zeta_j + \varepsilon_{ij}. \tag{B.2}$$

[29] At the time of writing, the biggest sample size of a GWAS is 5.4 million (Yengo et al., 2022).

This produces a list of β_j^{GWAS} coefficients for all J SNPs. The set of control variables $\mathbf{x}_i$ is usually very sparse, typically including age and sex alongside the first (usually ten) principal components (PCs) of the genetic data to account for population stratification (Price et al., 2006).[30] Importantly, due to the sparsity of control variables and the correlation between closely spaced SNPs, β_j^{GWAS} is not necessarily equal to β_j.

The set of β_j^{GWAS} coefficients is a simple linear projection of the outcome of interest on the space spanned by the measured SNPs. Imposing linearity neglects any form of interaction, whether gene–gene or gene–environment, thereby estimating a weighted average of the SNP-outcome associations over multiple environments (e.g., Løken et al., 2012). The consequences for the resulting $PGI \times E$ interaction term are discussed in Section 3.3.

Running so many regressions requires a correction for multiple hypothesis testing. Considering that there are $\sim$1 million approximately independent blocks of SNPs in the human genome (adjacent SNPs are often in linkage disequilibrium, i.e., inherited together), the commonly used criterion for genome-wide statistical significance is $p < 5 \times 10^{-8}$ (i.e., 0.05 divided by 1,000,000). The stringent significance level, in combination with the tiny effect sizes of individual SNPs on outcomes (Rietveld et al., 2013; Chabris et al., 2015), necessitates the use of extremely large samples to ensure adequate power. Legal and privacy reasons usually prohibit the joint analysis of genetic datasets. For this reason, researchers typically pursue a meta-analysis strategy to obtain a sufficiently large discovery sample (Visscher et al., 2017). Consortia such as the Social Science Genetic Association Consortium (SSGAC), Genetic Investigation of ANthropometric Traits (GIANT) and GWAS & Sequencing Consortium of Alcohol and Nicotine Use (GSCAN) have been key to this, coordinating the analyses (i.e., harmonizing the outcomes, quality-controlling consortium datasets) and bringing together the results of large numbers of smaller datasets. In such meta-analyses, only GWAS summary results (the effect sizes for each SNP) are shared between consortium members, addressing the legal and privacy barriers to their joint use. The GWAS meta-analysis approach has made possible an unprecedented surge in genetic discoveries that replicate consistently (Visscher et al., 2017).[31]

GWAS results are typically presented using so-called Manhattan plots. As an example, Figure B.1 provides the Manhattan plot visualizing the results of the second GWAS of educational attainment (Okbay et al., 2016). The x-axis of the Manhattan plot represents the position of the SNP in the genome (the numbers 1-22 reflect the autosomal chromosomes) and the y-axis the strength of the evidence for an association with the outcome variable (as reflected in the p value). The p value is transformed (by taking the negative of the 10 log of the p value) so that higher values represent stronger associations. Specifically, when a dot (representing a single SNP) is above the dashed line in the Manhattan plot (note that $-\log_{10}(5 \times 10^{-8}) = 7.3$), the SNP is genome-wide significant. The Manhattan plot also visualizes the effect of linkage disequilibrium. Because SNPs physically close to one another are more likely to be inherited together (i.e., are

[30] Population stratification is a form of confounding where the genetic makeup of ancestors influences one's genetic makeup as well as the outcome through nongenetic pathways (see also Appendix A). More specifically, if a population is stratified into subpopulations that do not mate randomly and an outcome happens to be more common in one subpopulation for non-genetic reasons, then the outcome will appear to be correlated with any SNPs that also happen to be more common in that sub-population. A commonly used hypothetical example is the "chopstick gene" (Hamer, 2000), where people of Asian descent have different allele frequencies and tend to eat with chopsticks for cultural reasons. A GWAS investigating the genetic basis of chopstick use without controlling for ancestral differences in allele frequencies would then pick up a "chopstick gene". By conditioning on principal components, the researcher essentially compares individuals within a common lineage and from the same genetic pool. For this reason, it is not necessary to include principal components in a within-family analysis.

[31] By contrast, so-called candidate-gene studies, a hypothesis-driven approach, have weak replication records (see e.g., Hewitt, 2012; Chabris et al., 2013).

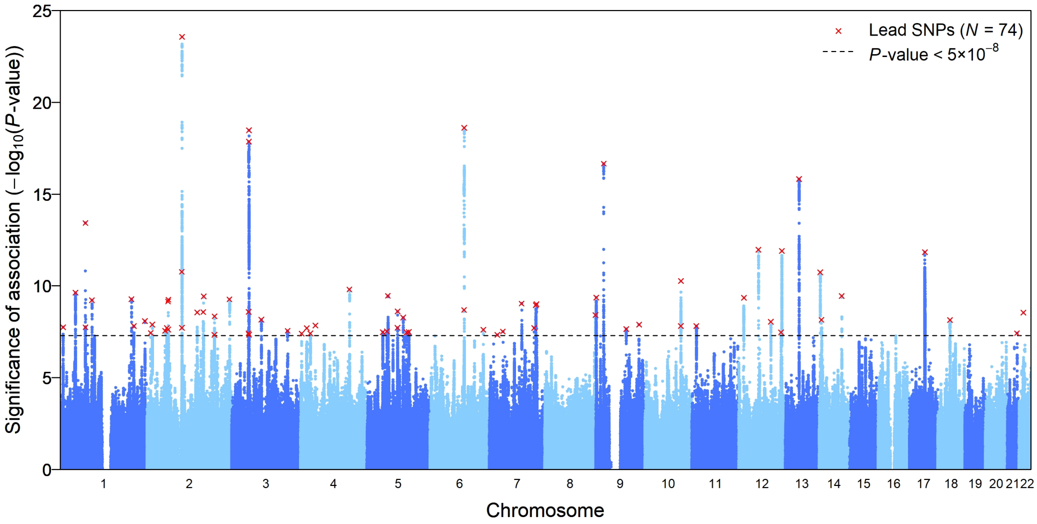

Figure B.1: Manhattan plot visualizing the results of the second genome-wide association study on educational attainment by Okbay et al. (2016).

in linkage disequilibrium), the regression results are very similar for adjacent SNPs. As a result, p values of adjacent SNPs are highly correlated. This is visible in the towers of dots around genome-wide significant SNPs. The result looks like the skyscrapers of Manhattan towering above lower-level buildings.

Because of linkage disequilibrium, each genome-wide significant SNP is correlated with adjacent SNPs. Each block of correlated SNPs is called a genome-wide significant locus. One typically picks a lead SNP: the SNP in a genome-wide significant locus with the smallest p value. By construction, the set of lead SNPs are therefore approximately uncorrelated with each other. The first GWAS meta-analysis of educational attainment used a combined sample of $\sim$125,000 people (Rietveld et al., 2013) and identified 3 genome-wide significant loci. The second GWAS of educational attainment used a sample of $\sim$400,000 people (Okbay et al., 2016) and identified 74 genome-wide significant loci. The third, Lee et al. (2018), used a sample of $\sim$1.1 million individuals to uncover 1,271 lead SNPs, and the fourth GWAS of educational attainment used $\sim$3 million individuals and identified 3,952 lead-SNPs associated with educational attainment (Okbay et al., 2022). This rapid growth in the power of genetic discovery exemplifies the genetics revolution that we are in the midst off.

B.3 Polygenic indices

The tiny explanatory power of individual SNPs has led researchers to develop methods that combine individual SNPs into so-called polygenic indices (PGIs), which have substantially greater explanatory power. A PGI is a weighted sum of individual SNPs and reflects the best linear genetic predictor of the outcome (e.g., Mills et al., 2020; Becker et al., 2021). It is constructed with the aim of predicting the genetic propensity toward a certain trait for individuals in a hold-out sample. For reasons of statistical independence, the hold-out sample cannot have been part of the original GWAS meta-analysis.

In its most basic form, a PGI is constructed as follows:

$$PGI_i = \sum_{j=1}^{J} \beta_j^{GWAS} G_{ij}, \tag{B.3}$$

where G_{ij} is again the number of copies of the minor allele for individual i and SNP j and β_j^{GWAS} are the β coefficients for SNP j (see Equation B.2) from the corresponding GWAS (Dudbridge, 2013). By multiplying SNP j (taking values $x_{ij} = \{0, 1, 2\}$) with its β_j^{GWAS} weight, SNPs with large effect sizes are weighted higher than those with small effect sizes. The simplest PGIs follow Equation B.3 where, given the linkage disequilibrium (LD) between SNPs, only one out of each genome-wide significant locus is maintained in the computation of the PGI.[32]

PGIs that include all available SNPs (i.e., genome-wide significant as well as nonsignificant SNPs) typically explain most variation in the outcome (Ware et al., 2017). The predictive accuracy of a PGI is also an increasing function of the sample size of the GWAS (Dudbridge, 2013). As GWAS samples grow, the estimates of the β_j^{GWAS} coefficients improve, and measurement error in the PGI is reduced. For example, whereas the PGI based on the first successful GWAS on educational attainment ($N \sim 125,000$) explained 3-4% of the variance in educational attainment out-of-sample (Rietveld et al., 2014), the PGI based on the results of a second, third and fourth GWAS explained 6-8%, 11-13% and 13-16% of the variation in educational attainment respectively (Okbay et al., 2016; Lee et al., 2018; Okbay et al., 2022). The maximum explained variance of a PGI is determined by the phenotype's so-called SNP-based heritability. Using methods like Genome-based Restricted Maximum Likelihood (GREML) estimation (Yang et al., 2011), several studies have shown that the SNP-based heritability of educational attainment is around 25% in developed countries (Rietveld et al., 2013). In other words, today's PGI for educational attainment already explains a bit more than half the variation in educational attainment that is achievable.[33] Van Kippersluis et al. (2023) review and assess statistical approaches to correct the coefficient of the PGI for measurement error in a regression. These approaches are well-suited for purging measurement error in the main effect of the PGI in between-family settings, and relevant to use as long as the maximum explained variance of a PGI has not been reached. Because the PGI has no natural metric, its effects are typically reported in standard deviations on an underlying latent scale of (loosely speaking) genetic propensity (Becker et al., 2021).

B.4 Epigenetics

Here, we briefly discuss the relationship between $G \times E$ interplay and epigenetics. Genetic variants are fixed at conception and do not change over the lifetime. This makes them well-suited as right-hand side variables to study their effects on later-life outcomes, possibly in interaction with environments. In contrast, epigenetics is the study of the expression of genes, which by definition varies over time, and often also by tissue (e.g., expression in blood may be different from expression in skin tissue). This makes epigenetics more suited as a left-hand side variable (as done for instance in the epigenome-wide association study on educational attainment; Karlsson Linnér et al. (2017)). If there is gene expression at a certain SNP, then epigenetics could be one mechanism through which $G \times E$ effects come into existence. However, there is no one-on-one translation from epigenetics to $G \times E$ interplay:

[32] More sophisticated construction measures exist that leverage machine learning to account for LD (see, for example, So and Sham, 2017; Vilhjalmsson et al., 2015), ancestry (Marnetto et al., 2020), or biological functioning (Liu et al., 2020; Choi et al., 2023), with typically better predictive power. However, all approaches have in common that they aggregate the genetic contributions of millions of small SNP-effects across the genome and are similar in spirit to the basic (and still used) approach of the linear weighted sum in Equation B.3.

[33] Note that PGIs can also be used to explain variation in other traits than the trait on which it has been calibrated through the GWAS weight. For example, the EA PGI has been used to explain wealth (Papageorge and Thom, 2020) and health (Bolyard and Savelyev, 2024) and in the present work we use it to explain test scores.

- Epigenetics may also affect genetic variants that are not polymorphisms (i.e., that do not vary across humans). In contrast, $G \times E$ interactions can by definition only exist if there is variation in G (SNPs);

- Even if a certain SNP exhibits expression, there could be compensatory investments that neutralize the initial $G \times E$ interaction;

- Even if a certain SNP does not exhibit gene-expression, there could still be $G \times E$ if certain 'type' of individuals simply respond differently to a given environment.

In sum, epigenetics is perfectly compatible with $G \times E$ and it is one of many potential mechanisms through which $G \times E$ may arise. However, it is neither a necessary nor sufficient condition for $G \times E$ (see Baker et al. (2024) for further discussion).

C Discussion and illustrative derivations of biases

The basic data generating process from Section 3.1 is repeated here. The outcome Y_i of child i is a function of her genotype G_i, and the genotype of her mother $G_{m(i)}$ and of her father $G_{f(i)}$:

$$Y_i = \beta_0 + \beta_G G_i + \beta_{G_m} G_{m(i)} + \beta_{G_f} G_{f(i)} + \varepsilon_i, \tag{C.1}$$

where β_0 is a constant term, β_G captures the direct genetic effect of the child's genotype G_i, and ε_i denotes the error term. Kong et al. (2020) further specify the parameters β_{G_m} and β_{G_f} as $\beta_{G_m} = \eta_m + w$ and $\beta_{G_f} = \eta_f + w$, where η_m and η_f denote genetic nurturing effects from the mother and father, respectively, and w captures all confounding effects that have not been adjusted for, including assortative mating, sibling interactions, and contributions from older ancestors.

Our ideal experiment discussed in Section 3.1 includes parental genotypes $G_{m(i)}$ and $G_{f(i)}$ in both the GWAS as well as the $G \times E$ analysis stage. Assume for simplicity that G_i, $G_{m(i)}$ and $G_{f(i)}$ are all scalars with variance 1. Using a parent-child or sibling GWAS then will provide consistent estimates for β_G, β_{G_m} and β_{G_f}. In turn, the true PGIs could be constructed as $PGI_i = \beta_G G_i$, $PGI_{m(i)} = \beta_{G_m} G_{m(i)}$, and $PGI_{f(i)} = \beta_{G_f} G_{f(i)}$. If in the analysis stage PGIs from both the child as well as both parents are included,

$$Y_i = \delta_0 + \delta_G PGI_i + \delta_{G_m} PGI_{m(i)} + \delta_{G_f} PGI_{f(i)} + e_i, \tag{C.2}$$

then the δ parameters all provide unbiased estimates that converge to 1 asymptotically if the analysis sample has a perfect genetic correlation with the GWAS sample, the outcomes are identically measured, and the exact same SNPs are used in both the GWAS and construction of the PGIs. In the following subsections, we discuss various deviations from this ideal case.

C.1 Sibling effects

We first consider the case where there are two siblings and there is a direct effect β_S of one's sibling's genotype on the other siblings' phenotype:

$$\begin{aligned} Y_{1j} &= \beta_{0j} + \beta_G G_{1j} + \beta_S G_{2j} + \varepsilon_{1j} \\ Y_{2j} &= \beta_{0j} + \beta_G G_{2j} + \beta_S G_{1j} + \varepsilon_{2j}. \end{aligned} \tag{C.3}$$

In case we would be controlling for the parental genotypes $G_{m(i)}$ and $G_{f(i)}$, then both β_G and β_S would be estimated without bias, since offspring genotypes are random and conditionally independent. In a family fixed effects specification, however, this is not the case. When taking sibling differences to eliminate the family fixed effects, we obtain:

$$Y_{1j} - Y_{2j} = (\beta_G - \beta_S)(G_{1j} - G_{2j}) + (\varepsilon_{1j} - \varepsilon_{2j}).$$

When β_S is positive (negative), sibling effects cause a downward (upward) bias in the estimate of the effect of one's own genotype G, as measured by β_G.

C.2 Interaction terms in family fixed effects models

Here, we consider the case with interaction terms in a family fixed effects model. If we add an interaction term to Equation 3:

$$\begin{aligned} Y_{ij} &= \beta_{0j} + \beta_G G_{ij} + \beta_E E_{ij} + \beta_{G\times E}(G_{ij} \times E_{ij}) + \varepsilon_{ij} \\ &= \beta_G \left(G_{ij} - \overline{G}_j\right) + \beta_E \left(E_{ij} - \overline{E}_j\right) + \beta_{G\times E}\left[(G_{ij} \times E_{ij}) - \left(\overline{G_{ij} \times E_{ij}}\right)\right]. \end{aligned} \tag{C.4}$$

The main effects of G and E are identified based on within-family variation, but the interaction term is not, since the deviation from the within-family product is not the same as the product of the within-family deviation.

There is no perfect solution to this issue. Indeed, specifying an interaction between the family deviations $\left(G_{ij} - \bar{G}_j\right)$ and $\left(E_{ij} - \bar{E}_j\right)$ (rather than the deviation from the within-family product) is not a fixed effect specification as it would be a nonlinear transformation of the original interaction term since some observations would interact two negative values. It, therefore, generally does not generate meaningful estimates (Shaver, 2019). So-called segmented (or stratified) regressions are sometimes performed (Shaver, 2019), where one runs a within-family analysis to obtain the effect of G stratified by different values of E, and vice versa. These analyses gauge whether the results are consistent with a positive or negative interaction. However, this solution is imperfect since stratification is based on potentially endogenous variables G and E. An arguably more convincing approach would be to control for (imputed) parental genotypes directly in the specification with an interaction term. See, for instance, Muslimova et al. (2024) for an empirical illustration and comparison of these different approaches.

C.3 Not controlling for parental genotypes in GWAS and analysis stage

In this subsection, we derive the consequences of not controlling for parental genotypes in the GWAS and/or analysis stage. Consider Equation C.1 and assume random mating for simplicity. Under random mating, the correlation between the child's genotype G_i and that of her parents is 0.5.[34]

Parental genotypes in GWAS, but *not* in analysis stage Let's first consider the case where one did include parental genotypes in the GWAS stage, but did not include parental PGIs in the analysis stage. In this case, we would estimate the true $PGI_i = \beta_G G_i$. In turn, since we do not include parental PGIs in the analysis stage:

$$\begin{aligned} Y_i &= \delta_0 + \delta_G PGI_i + e \\ &= \delta_0 + \delta_G \beta_G G_i + e. \end{aligned} \tag{C.5}$$

We can now derive that:

$$\begin{aligned} \hat{\delta}_G &= \frac{Cov(Y_i, \beta_G G_i)}{V(\beta_G G_i)} \\ &= \frac{Cov(\beta_0 + \beta_G G_i + \beta_{G_m} G_{m(i)} + \beta_{G_f} G_{f(i)} + \varepsilon_i, \beta_G G_i)}{V(\beta_G G_i)} \\ &= 1 + \frac{Cov(\beta_{G_m} G_{m(i)} + \beta_{G_f} G_{f(i)}, \beta_G G_i)}{V(\beta_G G_i)} \\ &= 1 + \frac{0.5\beta_{G_m} + 0.5\beta_{G_f}}{\beta_G}. \end{aligned} \tag{C.6}$$

Since β_{G_m} and β_{G_f} tend to have the same sign as β_G, coefficient δ_G would be overestimated because of the correlation between PGI_i and the parental genotypes that are left out of the analysis stage.

[34] In practice, the correlation between siblings tends to be a little larger due to assortative mating. For example, Torvik et al. (2022) show how the raw correlation between sibling's EA PGI is around 0.55.

Parental genotypes not in GWAS, nor in analysis stage Now consider the common case where one did not control for parental genotypes in the GWAS stage, and also does not control for parental PGIs in the analysis stage. If we exclude parental genotypes from the GWAS stage, the child's estimated PGI is given by:

$$PGI_i = \left(\beta_G + 0.5\beta_{G_m} + 0.5\beta_{G_f}\right) G_i. \tag{C.7}$$

In turn, since we do not include parental PGIs in the analysis stage:

$$\begin{aligned} Y_i &= \delta_0 + \delta_G PGI_i + e \\ &= \delta_0 + \delta_G \left(\beta_G + 0.5\beta_{G_m} + 0.5\beta_{G_f}\right) G_i + e. \end{aligned} \tag{C.8}$$

We can now derive that:

$$\begin{aligned} \hat{\delta}_G &= \frac{Cov(Y_i, \left(\beta_G + 0.5\beta_{G_m} + 0.5\beta_{G_f}\right) G_i)}{V(\left(\beta_G + 0.5\beta_{G_m} + 0.5\beta_{G_f}\right) G_i)} \\ &= \frac{Cov(\beta_0 + \beta_G G_i + \beta_{G_m} G_{m(i)} + \beta_{G_f} G_{f(i)} + \varepsilon_i, \left(\beta_G + 0.5\beta_{G_m} + 0.5\beta_{G_f}\right) G_i)}{\left(\beta_G + 0.5\beta_{G_m} + 0.5\beta_{G_f}\right)^2 V(G_i)} \\ &= \frac{\beta_G^2 + 0.5\beta_{G_m}\beta_G + 0.5\beta_{G_f}\beta_G + 0.5\beta_{G_m}\beta_G + 0.25\beta_{G_m}^2 + 0.25\beta_{G_m}\beta_{G_f} + 0.5\beta_{G_f}\beta_G + 0.25\beta_{G_f}\beta_{G_m} + 0.25\beta_{G_f}^2}{\left(\beta_G + 0.5\beta_{G_m} + 0.5\beta_{G_f}\right)^2} \\ &= 1. \end{aligned} \tag{C.9}$$

Substituting $\delta_G = 1$ back into Equation C.8 shows that the coefficient of G_i does not just capture the direct genetic effect β_G, but instead also reflects the influence of parental genotypes and hence is overestimated whenever β_{G_m} and β_{G_f} are larger than zero. Thus, as this will typically be the case, not including parental PGIs in the analysis stage will lead to an upward bias in the coefficient of the direct genetic effect.

Parental genotypes not in GWAS, but only in analysis stage Finally, consider the case where one did not control for parental genotypes in the GWAS stage, but does include parental PGIs in the analysis stage. As before, if we exclude parental genotypes from the GWAS stage, the child's estimated PGI is given by equation Equation C.7, and the corresponding parental estimated PGIs are given by:

$$PGI_p = \left(\beta_G + 0.5\beta_{G_m} + 0.5\beta_{G_f}\right) G_p. \qquad p = m, f \tag{C.10}$$

In the analysis stage, we estimate:

$$\begin{aligned} Y_i = \delta_0 &+ \delta_G PGI_i + \delta_{G_m} PGI_m + \delta_{G_f} PGI_f + e \\ &= \delta_0 + \delta_G \left(\beta_G + 0.5\beta_{G_m} + 0.5\beta_{G_f}\right) G_i + \delta_{G_m} \left(\beta_G + 0.5\beta_{G_m} + 0.5\beta_{G_f}\right) G_m \\ &\quad + \delta_{G_f} \left(\beta_G + 0.5\beta_{G_m} + 0.5\beta_{G_f}\right) G_f + e. \end{aligned} \tag{C.11}$$

Comparing Equation C.11 to the true data generating process from Equation C.1, it follows that:

$$\delta_G \left(\beta_G + 0.5\beta_{G_m} + 0.5\beta_{G_f}\right) = \beta_G, \tag{C.12}$$

implying that

$$\delta_G = \frac{\beta_G}{\left(\beta_G + 0.5\beta_{G_m} + 0.5\beta_{G_f}\right)}. \tag{C.13}$$

Substituting δ_G from Equation C.13 back into Equation C.11 shows that one can correctly recover the direct genetic effect δ. Essentially, the coefficient of the misspecified PGI is scaled downwards (since $\delta_G < 1$ in Equation C.13) such that the estimated coefficient of the PGI only reflects the direct genetic effect. This is a special case however, one that corresponds to $\rho_g = 1$ in Trejo and Domingue (2018), where the correlation between the direct genetic effect and the genetic nurture effect is equal to 1. Intuitively, even though the GWAS coefficients used to construct the PGI are wrong, since the direct genetic effect β_G and the genetic nurture effects β_{G_m} and β_{G_f} are proportional to each other (have correlation 1), including both the child's PGI and the parental PGIs enables to accurately scale these coefficients to retrieve the true direct genetic effects and genetic nurture effects.

In general, this proportionality does not hold (Trejo and Domingue, 2018). Consider again a highly stylized model to gain some intuition. We extend the model to two uncorrelated genetic variants G_1 and G_2, and to keep the notation tractable assume just one parent p and ignore individual subscripts. Thus, the new data generating process is given by:

$$Y = \beta_0 + \beta_{G_1} G_1 + \beta_{G_2} G_2 + \beta_{G_{1p}} G_{1p} + \beta_{G_{2p}} G_{2p} + \varepsilon. \tag{C.14}$$

If we exclude parental genotypes from the GWAS stage, the resulting PGI is estimated as

$$PGI = \left(\beta_{G_1} + 0.5\beta_{G_{1p}}\right) G_1 + \left(\beta_{G_2} + 0.5\beta_{G_{2p}}\right) G_2, \tag{C.15}$$

and the parental PGI_p is constructed analogously. If we now include the parental PGI as a control variable in the regression

$$\begin{aligned} Y &= \delta_0 + \delta_G PGI + \delta_{G_p} PGI_p + e \\ &= \delta_0 + \delta_G \Big[\left(\beta_{G_1} + 0.5\beta_{G_{1p}}\right) G_1 + \left(\beta_{G_2} + 0.5\beta_{G_{2p}}\right) G_2 \Big] \\ &\quad + \delta_{G_p} \Big[\left(\beta_{G_1} + 0.5\beta_{G_{1p}}\right) G_{1p} + \left(\beta_{G_2} + 0.5\beta_{G_{2p}}\right) G_{2p} \Big] + e. \end{aligned} \tag{C.16}$$

To correctly retrieve the direct genetic effects β_{G_1} and β_{G_2}, the following equalities should hold:

$$\begin{aligned} \delta_G \left(\beta_{G_1} + 0.5\beta_{G_{1p}}\right) &= \beta_{G_1}, \\ \delta_G \left(\beta_{G_2} + 0.5\beta_{G_{2p}}\right) &= \beta_{G_2}. \end{aligned} \tag{C.17}$$

Or, rearranged,

$$\begin{aligned} \delta_G &= \frac{\beta_{G_1}}{\left(\beta_{G_1} + 0.5\beta_{G_{1p}}\right)} = \frac{1}{1 + 0.5\frac{\beta_{G_{1p}}}{\beta_{G_1}}}, \\ \delta_G &= \frac{\beta_{G_2}}{\left(\beta_{G_2} + 0.5\beta_{G_{2p}}\right)} = \frac{1}{1 + 0.5\frac{\beta_{G_{2p}}}{\beta_{G_2}}}. \end{aligned} \tag{C.18}$$

Clearly, the two equalities of Equation C.18 are only satisfied if

$$\frac{\beta_{G_{1p}}}{\beta_{G_1}} = \frac{\beta_{G_{2p}}}{\beta_{G_2}}. \tag{C.19}$$

Thus, only when for all SNPs the genetic nurture effects are exactly proportional to the direct genetic effects (i.e., their correlation is 1), then it is possible to rescale the PGI coefficient to obtain the correct direct genetic effect using parameter δ_G. In case the correlation between the direct genetic and genetic nurture effects is not perfect, there will be a downward bias in the estimated direct genetic effects. For a more general discussion, see Trejo and Domingue (2018).

C.4 Endogeneity of E

Endogeneity of E may arise from five sources: reverse causality, omitted variable bias (especially from correlations with parental genotypes), measurement error, mediation, and correlation of the GWAS sample selection with the environment E. The last two sources of bias are discussed in the main text in Section 3.2.4. We discuss the other sources of bias here. We focus specifically on the resulting bias in the estimated coefficients of Equation 1 in a setting with gene–environment interplay.

Bias arising from reverse causality: Especially in the case of a non-predetermined E, there may be reverse causality, with the outcome influencing the relevant environment. Such endogeneity will bias the estimated effect of E and $G \times E$ but not the effect of G if parental genotypes are used as controls.

Bias arising from omitted environmental variables: Omitted variable bias can arise because environments typically do not arise in isolation. For instance, education, employment, and income are correlated with each other and with unobserved confounders. Therefore, even if a significant E (or $G \times E$) is found, it is difficult to identify whether the association is driven by income, employment, education, or something else (Boardman et al., 2013). Hence, endogeneity of E implies that the coefficient on $G \times E$ may in fact reflect $G \times E^*$, i.e., a causal effect of some environment E^* that is correlated with E. This is shown in the last two columns of Table 1.

Bias arising from measurement error: A third source of endogeneity in E is measurement error. If there is no rGE, then standard econometric theory explains how classical measurement error in E will lead to attenuation bias. If there is no measurement error in E but there is correlation between G and E, the implications are more subtle: the known measurement error in PGIs as a proxy for G will also lead to measurement error in E. Active rGE implies that G leads to self-selection into certain environments E. If in turn G is measured with error, then not only is there attenuation bias in the coefficient of G, but also the coefficient of E will be biased since the measurement error in G introduces omitted variable bias in E.

D Representative examples from the literature

We here discuss a representative study for each of the cells of Table 1.[35] A first observation from Table 1 is that there are currently no studies that exploit PGIs based on parent-child trio GWASs or within-family GWASs – that is, there are no examples in the first row of the table. The first within-family GWASs (Howe et al., 2022; Tan et al., 2024) were only recently published and to the best of our knowledge no studies exist that employed these summary statistics to construct a within-family PGI in a $G \times E$ analysis. Working from top-left to bottom-right, Muslimova et al. (2024) is a study that uses a within-family design in the UK Biobank to study the interaction between the EA PGI and birth order to explain variation in educational attainment. Following the literature, they interpret birth order as a proxy for parental investments. The inclusion of family fixed effects renders the variation in PGIs across siblings random, and as an additional advantage one's birth order is also plausibly randomly assigned within-families. Indeed, the authors demonstrate that birth order and genetic variation are independent of one another, and that birth order-induced variation in educational attainment is thus environmental in origin. Muslimova et al. (2024) show how both the EA PGI and birth order causally increase educational attainment. Furthermore, there is a non-negligible interaction between the two: firstborns with a polygenic index two standard deviations above the mean complete on average one extra year of education compared to their laterborn siblings with a similar polygenic index. Apparently, firstborn children benefit disproportionally from having a higher EA PGI, which is consistent with a the presence of a complementarity between endowments and investments in human capital production.

Houmark et al. (2022) use the Danish iPSYCH data linked to administrative records on children's reading scores in elementary school to study the interaction between the EA PGI and an index of socioeconomic status (SES) of the family as measured by parental income and education. Their main results reflect between-family comparisons, but they also report findings from a smaller subsample of siblings. The findings from the within-family design are in the same direction, but they often lack statistical power since the sample size is substantially smaller, and the variation in the SES index is very limited across siblings. The authors show that both genetic as well as socioeconomic gaps in reading test scores between children increase throughout elementary school. Given their between-family design, they acknowledge that there exists some correlation across their SES measures and the PGI, but they also provide plausible evidence that the gaps largely open up independently. Their main finding is that the increase in the importance of the EA PGI with grade is completely driven by low SES children. This shows that a high EA PGI eventually leads to higher reading test scores among children from lower SES backgrounds.

Cheesman et al. (2022) draw on the Norwegian Mother, Father and Child Cohort (MoBa) data linked to Norwegian administrative records to study the interaction between the EA PGI and the social context as measured by schools and residential areas, on standardised national test results in maths, reading and English. In their analyses, they are able to control for parental EA PGIs ensuring that the variation in the child's EA PGI is exogenous. The authors use multilevel models where they allow for interactions between the EA PGI and random effects for the school and residential areas (neighborhood, district, and municipality). Their main finding is that the effect of the EA PGI is weaker in higher-performing schools: Differences between schools explain 4% versus 2% of the variance in educational achievement for children scoring 2 standard

[35] Table G.1 in Appendix G includes the full list of studies on $G \times E$ interplay in human capital drawing on the EA PGI; for an extensive review of the cross-disciplinary literature on $G \times E$ interplay we refer to recent reviews (Domingue et al., 2020; Dias Pereira et al., 2022).

deviations below versus 2 standard deviations above the mean of the EA PGI. In contrast, there is no significant interaction between the EA PGI and residential environments. Whereas the study provides convincing evidence of $G \times E$ interactions in education, due to the endogeneity and latent measurement of school quality through random effects, they cannot identify the exact characteristic of the school that drives this pattern.

Moving to the bottom row of Table 1, we have Schmitz and Conley (2017) being one of the earliest examples of a $G \times E$ analysis that exploits a plausibly exogenous E. The authors use the US Health and Retirement Study (HRS) to study the interaction between the EA PGI and Vietnam-era military service on educational attainment. In contrast to the UK Biobank, iPSYCH and MoBa datasets used in the studies reviewed above, the HRS does not include family-members (other than spouses). Therefore, the authors rely on a between-family design in which the EA PGI reflects both direct genetic effects as well indirect genetic effects. Schmitz and Conley (2017) exploit an exogenous source of environmental exposure, the Vietnam-era draft lotteries, and thereby ensure that their G and E measures are not correlated to each other. Although their estimates do not reach statistical significance, possibly because of low statistical power, their findings suggest that draft eligible men with lower EA PGI scores completed fewer years of schooling.

Papageorge and Thom (2020) also use HRS to study the interaction between various measures of parental SES and the EA PGI on educational attainment. The authors transparently show a modest association between the EA PGI and parental SES measures, including father's income and unemployment, and whether the family was financially well-off. Papageorge and Thom (2020) do control for parental education, which may go a long way in reducing concerns of rGE and endogeneity of G. Their main finding is that the EA PGI has a negative interaction with parental SES in obtaining a high school degree, but a positive interaction in obtaining a college degree. Strengths of the relationships differ depending on the model specification. Interestingly, it seems that at lower levels of educational attainment, parental SES may substitute for genetic endowments, while parental SES could complement genetic endowments in college completion.

Finally, Arold et al. (2022) use the U.S. National Longitudinal Study of Adolescent to Adult Health (AddHealth) to study the interaction between the EA PGI and school quality on educational attainment. They measure school quality as a composite measure (a principal component) capturing teacher experience, teacher turnover, teacher education, and class size. Whereas the authors adopt a between-family design in their main analysis and acknowledge that school quality may be endogenous, Arold et al. (2022) carefully address these concerns through a control function approach including rich measures of parental background and auxiliary analyses in a smaller sibling subsample. Their main finding is that the EA PGI and school quality are substitutes: a one-standard deviation increase in school quality reduces the effect of a one-standard deviation increase of the EA PGI by 20%. Thus, high-quality teachers improve the chances of students with low genetic endowments to complete college.

These six representative studies draw on five datasets, from which the UK Biobank and the HRS are currently included in the polygenic index repository (Becker et al., 2021). Together, these six representative studies show that there is robust evidence of non-negligible $G \times E$ interactions in educational attainment. Returning to the motivations for studying $G \times E$ interplay from the introduction, even though all studies provide ex-post some theoretical grounding for the empirical findings, none of them offers a direct test of theoretically substantiated hypothesis. All six studies speculate about the mechanisms through which genetic factors operate, and some provide additional empirical analyses to substantiate such speculations (Muslimova et al., 2024; Houmark et al., 2022; Papageorge and Thom, 2020; Arold et al., 2022). Finally, all studies are informative about treatment effect heterogeneity, for some studies this analysis being the main

motivation (Cheesman et al., 2022; Schmitz and Conley, 2017).

Whereas we acknowledge that this is a selective set of studies that may be subject to publication bias, the estimates are also quantitatively meaningful. Where possible we benchmark the magnitude of the $G \times E$ coefficient to the main effect of E, i.e., the typical focus of studies in economics. In the preferred specification of Muslimova et al. (2024, Table 2, last column), the effect of birth order is 0.428 and the interaction term is estimated as 0.285, which constitutes 67% of the main effect. It is worth noting that this is the only study in which measurement error in G is addressed (using the approach developed by Van Kippersluis et al. (2023)). In Papageorge and Thom (2020, Table B.2 in the Online Appendix), the effect of father's income on college completion is estimated as 0.09 with an interaction term of 0.054 (i.e., 60% of the main effect), and in Arold et al. (2022, Table 3, column 1) the effect of teacher quality is estimated to be 0.128 with an interaction term of -0.062 (i.e., 48% of the main effect). At the lower end of the spectrum, Houmark et al. (2022, Table B1, column 3) estimate a coefficient of SES of 0.261 and an interaction term of 0.079, which represents roughly 30% of the main effect. Finally, Schmitz and Conley (2017, Table 4, column 3) the Vietnam draft has an estimated effect of -0.383 whereas the interaction term is non-significant and estimated to be -0.033 (i.e., 9% of the main effect). Given the random slopes model, the presented estimates in Cheesman et al. (2022) do not directly allow for a quantification of the interaction term with respect to the environmental main effect. Whereas the exact estimates vary depending on the environmental exposure, our selective set of studies shows that the interaction terms are typically a factor 1.5 to 3 smaller than the environmental main effect, but in virtually all cases meaningfully moderate the effect of the environmental exposure on educational attainment.

E Steps in gene-environment interplay analysis

E.1 Checklist

Here, following the steps taken in the empirical application in the main text, we provide a simple checklist for applied researchers interested in estimating a form of gene–environment interplay. We assume that by the time the researcher is going through the checklist, she has already made a decision regarding the construction of the variables Y, G, and E:

1. **Perform power calculations:** Statistical power calculations have been a crucial element in genoeconomic analyses from the onset of the field (Benjamin et al., 2012). Since the anticipated effect sizes of interaction terms are typically an order of magnitude smaller than those of the main effects (see Appendix D), it is strongly advised to conduct ex-ante power calculations before any empirical analysis is done. This avoids underpowered statistical tests and reduces the risk of finding false positives and negatives. Arguably the best way to mimic the actual statistical power of an empirical test for the interaction term is through simulations (Duncan and Keller, 2011). These allow one to specify the exact model that one intends to estimate and can account for possible adjustments to the standard error. In Appendix E.2, we provide the power calculations for our empirical application.

2. **Check for gene–environment correlations** rGE: Check the extent to which genes correlate with the environment of interest (both endogenous and exogenous E if possible). Finding no significant evidence for rGE would help support the assumption that a truly exogenous environment is exploited in the analyses.

3. **Investigate the correct functional form:** Although $G \times E$ analyses are often conducted using linear interaction terms, it is advised to use descriptive analyses to check for nonlinearities in the relationship. To be as nonparametric as possible, one could plot the relationship between G and Y separately for the treated and control groups. Any difference in the Y-G relationship between the treated and control groups is evidence in favor of $G \times E$. Differences in levels suggest that including G and E separately should suffice; differences in slopes between the two groups suggest a simple additive and linear specification with an interaction term; differences in only some parts of the distribution of the PGI suggest a more complex form of $G \times E$ interplay.[36]

4. **Perform $G \times E$ analysis:** Perform the $G \times E$ analyses using the functional form determined in the previous step. Include all relevant control variables (e.g., principal components when within-family designs are not used). Interpret the findings in view of the mechanisms described in Section 4.1 and assess the bias in the estimate based on Table 1. When exogeneity of G or E can only be guaranteed conditional on inclusion of certain control variables, one should always add interaction terms between each control variable and both the G and E terms. These additional interaction terms aim to capture any residual correlation between the controls and either genes or environment and are essential for estimating an unbiased effect of the interaction term (Keller, 2014; Feigenberg et al., 2023).

[36] For a more thorough discussion of nonparametric analyses of gene–environment interplay in the context of a structural equation model (SEM), see Briley et al. (2015). For more flexible functional forms additionally modeling possible heteroskedasticity, see Domingue et al. (2022).

5. **Correct inferences for heteroskedasticity and multiple hypothesis testing:** It is prudent to use robust standard errors in the analysis, and the analyst may wish to conduct heteroskedasticity tests as in Domingue et al. (2022) if the variance of the outcome is expected to vary systematically with the environmental exposure. If the analysis involves many different outcomes explored under a more data-driven approach, one may want to correct for multiple hypothesis testing.

E.2 Power calculations

We use simulations to perform *ex-ante* power calculations to estimate the minimum detectable effect (MDE) for the $G \times E$ coefficient in our setting. Since the interaction term captures a form of nonlinearity in the relationship between G, E and the outcome Y, simulations are arguably the most flexible and reliable way to approximate the actual statistical power of our empirical test (Duncan and Keller, 2011). We simulate the following (simplified) $G \times E$ model 1,000 times and approximate statistical power using the share of coefficients with a p value < 0.05:[37]

$$Y_i = \beta_0 + \beta_G G + \beta_E E + \beta_{G\times E}(G \times E) + \varepsilon. \tag{E.1}$$

We set the parameters based on empirical results in the literature. Since our outcome variable Y_i is always standardized to have mean 0 and standard deviation 1, we assume that $\alpha = 0$ and that the error term is drawn from a standard normal distribution $\varepsilon \sim N(0, 1)$. We also assume that G is standard-normally distributed ($G \sim N(0, 1)$), which is a good approximation of the distribution of the standardized PGI, and following Allegrini et al. (2019), we set $\beta_G = 0.259$. Since approximately half of our sample is treated (i.e., born between September and November), we randomly assign 50% of the simulated observations to have $E = 1$ and the other half $E = 0$. Following Crawford et al. (2010), we assume that $\beta_E = 0.90, 0.60, 0.35, 0.20$ and 0.13 when the outcome is the Entry Assessment) score, KS1, KS2, KS3, and KS4, respectively.[38] Finally, given our ALSPAC data, we set the sample size in the power calculations to $N = 1,000$ for the Entry Assessment score, $N = 3,500$ for Key Stages 1, 2 and 4, and $N = 3,000$ for Key Stage 3.

Figure E.1 shows the statistical power that we can expect for each outcome variable and for different magnitudes of the interaction coefficient $\beta_{G\times E}$ from Equation E.1. Following the literature,[39] we expect the magnitude of the interaction effect to be about one-quarter of the size of the main effect of the environment. Therefore, we expect $\beta_{G\times E} = 0.225, 0.15, 0.0875, 0.05$ and 0.0325 when the outcome is the Entry Assessment score, KS1, KS2, KS3, and KS4, respectively. Hence, we have more than 90% power to estimate the interaction effect for the Entry Assessment score and about 75% power for KS1, although we are underpowered for

[37] The STATA code for these simulations and a simplified example code for simulating the statistical power of the empirical specification is available at our GitHub or Zenodo repository. The example code allows one to specify a binary or continuous environment, different sample sizes, and other expected MDEs.

[38] Note that Crawford et al. (2010) do not report estimates for the Entry Assessment test. We hypothesize this to be 0.90, in line with the pattern of greater effect sizes for younger children. Indeed, as we show below, our results for the Key Stages are remarkably similar to these authors' estimates: 0.70 (KS1), 0.39 (KS2), 0.22 (KS3) and 0.28 (KS4), as shown in Table 5.

[39] See our discussion of the size of $G \times E$ estimates in the existing literature at the end of Appendix D. Regarding treatment effect heterogeneity in school starting age effects, Crawford et al. (2010) do not report this based on either individual or parental characteristics. However, Black et al. (2011) report heterogeneity of effects based on a composite measure of parental background (see Table 7 in their paper). Moving from the bottom 25% to the top 75% of the distribution (a shift equivalent to about 2 standard deviations in our PGI distribution), the effect of early age-at-entry more than doubles for outcomes such as education, earnings at age 35, and teenage pregnancy but does not differ for outcomes such as IQ or mental health. We take a conservative approach and consider a change of about one-quarter of the main effect of the environment.

the other outcomes. More generally, we are well powered ($> 80\%$) to estimate an interaction coefficient greater than 0.1 for the Key Stage outcomes and greater than 0.175 for the Entry Assessment test.

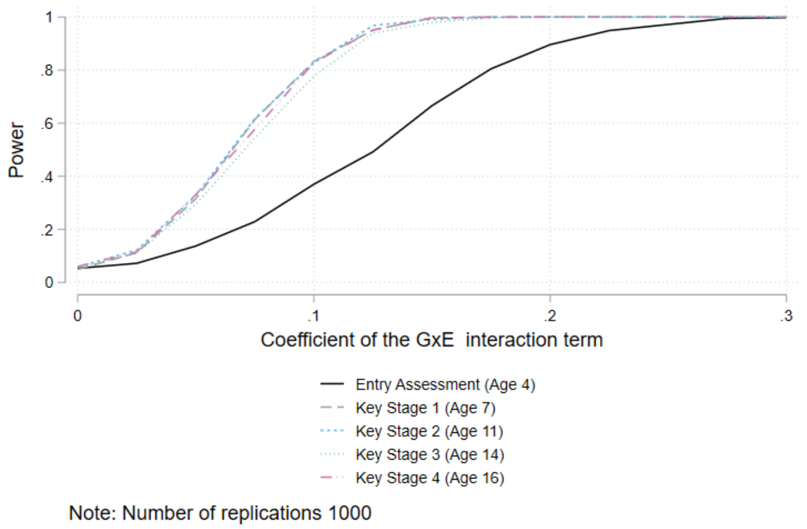

Figure E.1: Power calculations for detecting the interaction coefficients in Table 5.

F Robustness analyses

F.1 Non-linearities in G

In this Appendix, we explore the robustness of our estimates. First, although Figure 3 suggests that we can approximate the relationship between the PGI and the outcome as linear, we explore the robustness of our results to non-linearities in the PGI effect, following Section 4.1.

Table F.1 shows that the coefficient on PGI^2 is insignificantly different from zero in most specifications; the exception being for the KS3 (age 13-14) outcome. This again suggests that a linear relationship between the PGI and the outcome fits the data well. The addition of the square of the child's PGI also does not lead to large differences in the estimates of the control variables. Comparing Table F.1 to the same specification (but without quadratics) in Table 4 and Table 5 shows that the parameter of interest ($\delta_{G\times E}$) is generally robust to the inclusion of the quadratic term, though the addition of the parental PGIs increases the standard error somewhat, reducing the precision of the estimate.

Table F.1: OLS estimates of the main and interaction effects of being old-for-grade (Treated) and the EA PGI on children's test scores, allowing for non-linearities (quadratic) in the PGI effect.

	EA	KS1	KS2	KS3	KS4
Treated	1.148***	0.694***	0.384***	0.213**	0.277***
	(0.102)	(0.033)	(0.024)	(0.054)	(0.022)
PGI Child	-0.055	0.317***	0.353***	0.257***	0.312***
	(0.035)	(0.040)	(0.017)	(0.017)	(0.013)
PGI Child^2	-0.003	0.012	-0.037	-0.050**	0.011
	(0.096)	(0.022)	(0.022)	(0.019)	(0.020)
Treated × PGI Child	0.088*	-0.101	-0.085*	-0.000	-0.040
	(0.043)	(0.069)	(0.034)	(0.045)	(0.021)
MoB	-0.176***	-0.079***	-0.091***	-0.004	-0.036*
	(0.031)	(0.017)	(0.014)	(0.012)	(0.014)
Treated × MoB	0.086	0.036	0.062***	-0.009	0.026
	(0.055)	(0.018)	(0.015)	(0.028)	(0.019)
MoB × PGI Child	-0.070**	0.034***	0.007	-0.022	0.013***
	(0.023)	(0.005)	(0.014)	(0.014)	(0.002)
MoB × PGI Child^2	0.015	-0.004	-0.004	-0.020***	0.001
	(0.031)	(0.009)	(0.009)	(0.003)	(0.008)
MoB × PGI Child × Treated	0.139***	-0.003	0.028	0.067***	0.006
	(0.028)	(0.014)	(0.016)	(0.012)	(0.007)
MoB × PGI Child^2 × Treated	-0.016	0.012	0.018	0.035***	-0.006
	(0.061)	(0.014)	(0.015)	(0.007)	(0.012)
PGI Mother	0.017	0.079*	0.059***	0.113***	0.052***
	(0.054)	(0.037)	(0.012)	(0.022)	(0.005)
PGI Father	0.115*	-0.076	-0.024	0.005	-0.012
	(0.045)	(0.038)	(0.027)	(0.024)	(0.024)
PGI Mother × Treated	0.077	-0.009	0.022	-0.023	-0.004
	(0.061)	(0.058)	(0.016)	(0.035)	(0.033)
PGI Father × Treated	-0.125**	0.036	0.022	0.028	-0.005
	(0.048)	(0.042)	(0.032)	(0.050)	(0.034)
PGI Mother × PGI Child	0.034	-0.018	0.016	0.031	-0.000
	(0.048)	(0.023)	(0.016)	(0.017)	(0.017)
PGI Father × PGI Child	-0.012	-0.006	0.027	0.035	-0.003
	(0.055)	(0.017)	(0.020)	(0.028)	(0.020)
R^2	0.285	0.195	0.160	0.162	0.153
Observations	1094	3436	3610	3073	3579

Notes: The analysis uses a bandwidth of 3 months before and after the September cutoff (i.e., June till November). Additional control variables include gender, year of birth, the first 10 principal components, a dummy for missing parental PGI, and interactions of all covariates with PGI Child and with Treated. Robust standard errors in parentheses, clustered by month of birth. * $p < 0.10$, ** $p < 0.05$, *** $p < 0.01$.

F.2 Bandwidth

In our main specification we set the bandwidth to three months before and after September to balance bias and precision. This choice, however, remains somewhat arbitrary. Here we investigate the robustness of our results against using smaller or larger bandwidths. Table F.2 shows the results specifying different bandwidth for the RDD, including two and four months on either side of the threshold. Our main results are robust to the use of different bandwidths, with the estimate of interest generally being somewhat larger in the analysis with a two-month bandwidth, and smaller in the analysis with a four-months bandwidth. Despite the bigger sample size in the latter, the standard errors are consistently larger, and the estimates are not significantly different from zero.

Table F.2: OLS estimates of the main and interaction effects of being old-for-grade (Treated) and the EA PGI on children's test scores, exploring sensitivity to different bandwidths.

	Bandwidth 2 months					Bandwidth 4 months				
	EA (age 4-5)	KS1 (age 6-7)	KS2 (age 10-11)	KS3 (age 13-14)	KS4 (age 15-16)	EA (age 4-5)	KS1 (age 6-7)	KS2 (age 10-11)	KS3 (age 13-14)	KS4 (age 15-16)
Treated	1.078***	0.728***	0.334***	0.089***	0.226***	1.120***	0.646***	0.335***	0.221***	0.286***
	(0.043)	(0.007)	(0.002)	(0.004)	(0.005)	(0.056)	(0.037)	(0.024)	(0.056)	(0.028)
PGI Child	-0.046	0.325***	0.349***	0.243***	0.277***	0.027	0.249***	0.281***	0.213***	0.252***
	(0.053)	(0.025)	(0.044)	(0.031)	(0.018)	(0.062)	(0.038)	(0.028)	(0.010)	(0.026)
Treated × PGI Child	0.143*	-0.182**	-0.141**	-0.076*	-0.026	0.106	-0.065	-0.068	0.034	-0.004
	(0.045)	(0.032)	(0.034)	(0.028)	(0.019)	(0.060)	(0.062)	(0.037)	(0.034)	(0.032)
Month of Birth (MoB)	-0.005	-0.135***	-0.065***	0.024**	-0.005	-0.116***	-0.056***	-0.057***	-0.026**	-0.028***
	(0.014)	(0.004)	(0.001)	(0.004)	(0.004)	(0.021)	(0.014)	(0.013)	(0.008)	(0.005)
Treated × MoB	-0.145***	0.100***	0.068***	0.071***	0.017*	0.015	0.030*	0.040**	0.016	-0.003
	(0.022)	(0.011)	(0.007)	(0.005)	(0.005)	(0.023)	(0.014)	(0.014)	(0.019)	(0.011)
MoB × PGI Child	-0.114**	0.036**	0.053**	0.026*	0.012*	-0.007	0.005	-0.007	-0.004	0.006
	(0.030)	(0.009)	(0.015)	(0.011)	(0.005)	(0.035)	(0.016)	(0.013)	(0.004)	(0.007)
MoB × PGI Child × Treated	0.145*	0.039*	-0.009	0.034*	-0.004	0.018	0.006	0.032**	0.027**	0.002
	(0.061)	(0.015)	(0.023)	(0.012)	(0.018)	(0.039)	(0.019)	(0.013)	(0.009)	(0.009)
PGI Mother	-0.058	0.066	0.080**	0.132***	0.078***	0.046	0.105***	0.104***	0.161***	0.098***
	(0.031)	(0.030)	(0.014)	(0.019)	(0.004)	(0.053)	(0.029)	(0.017)	(0.016)	(0.016)
PGI Father	0.131**	-0.085**	0.023	0.092	0.047	0.112***	-0.032	0.039*	0.092***	0.065**
	(0.032)	(0.024)	(0.046)	(0.052)	(0.032)	(0.029)	(0.032)	(0.020)	(0.026)	(0.026)
PGI Mother × Treated	0.110**	0.023	0.016	-0.002	-0.005	0.035	-0.008	0.008	-0.011	-0.021
	(0.033)	(0.068)	(0.016)	(0.043)	(0.039)	(0.060)	(0.041)	(0.020)	(0.037)	(0.027)
PGI Father × Treated	-0.137***	0.091**	0.048	0.062	-0.004	-0.136***	0.062	0.045	-0.024	-0.011
	(0.017)	(0.029)	(0.038)	(0.055)	(0.026)	(0.037)	(0.045)	(0.034)	(0.051)	(0.039)
PGI Mother × PGI Child	0.033	-0.018	0.008	0.003	0.013	0.028	-0.011	-0.005	-0.004	-0.010
	(0.054)	(0.025)	(0.013)	(0.016)	(0.020)	(0.034)	(0.013)	(0.017)	(0.022)	(0.013)
PGI Father × PGI Child	-0.024	0.006	-0.002	0.009	-0.000	-0.027*	-0.002	-0.005	0.007	-0.009
	(0.018)	(0.015)	(0.008)	(0.016)	(0.014)	(0.014)	(0.013)	(0.010)	(0.013)	(0.010)
R^2	0.316	0.212	0.167	0.166	0.156	0.247	0.189	0.167	0.163	0.148
Observations	756	2383	2489	2128	2476	1419	4427	4679	4005	4653

Notes: The analysis uses a bandwidth of two or four months before and after the September cutoff. Additional control variables include gender, year of birth, the first 10 principal components, a dummy for missing parental PGI, and interactions of all covariates with PGI Child and with Treated. Robust standard errors in parentheses, clustered by month of birth. * $p < 0.10$, ** $p < 0.05$, *** $p < 0.01$.

G Additional Figures and Tables

Table G.1: Empirical examples of $G \times E$ studies using the EA PGI in gene–environment interaction models.

	Exogenous E	Endogenous E	
		Predetermined E	Non-predetermined E
Exogenous G (parent-child/sibling) & PGI on basis of parent–child GWAS			
Exogenous G (parent-child/sibling) & PGI on basis of regular GWAS	Muslimova et al. (2024, Birth order)<br>Barcellos et al. (2021, Education system)<br>Van den Berg et al. (2023a, Vaccination campaign)	Bates et al. (2018, Family circumstances)<br>Domingue et al. (2015, Family circumstances)<br>Houmark et al. (2022, Family circumstances)<br>Ronda et al. (2022, Family circumstances)<br>Pettersson (2023, Cohort/Family circumstances)	Cheesman et al. (2022, Social context)
Endogenous G (between family data) PGI on basis of regular GWAS	Ahlskog et al. (2024, Education system)<br>Fukushima et al. (2024, Pollution reduction programme)<br>Johnson et al. (2022, Education system)<br>Rimfeld et al. (2018, Collapse political system)<br>Schmitz and Conley (2017, Vietnam draft)<br>Ujma et al. (2022, Collapse political system)<br>Van den Berg et al. (2023b, Prenatal sugar consumption)<br>Von Hinke and Sørensen (2023, London smog)	Cheesman et al. (2020, Adoption status)<br>Conley et al. (2015, Family circumstances)<br>Conley et al. (2016, Cohort)<br>Conley et al. (2019, Birth weight)<br>Dias Pereira (2021, Cohort)<br>Fletcher (2023, Socioeconomic context)<br>Isungset et al. (2022, Family circumstances)<br>Lin (2020, Family circumstances/Cohort)<br>Mills et al. (2018, Socioeconomic context)<br>Muslimova et al. (2023, Cohort)<br>Okbay et al. (2016, Cohort)<br>Papageorge and Thom (2020, Family circumstances)<br>Selzam et al. (2017, Family circumstances)<br>Von Stumm et al. (2020, Family circumstances)<br>Uchikoshi and Conley (2021, Family/School circumstances)<br>Wickrama et al. (2021, Family circumstances)	Allegrini et al. (2020, Various, some predetermined)<br>Arold et al. (2022, Teacher quality)<br>Nagpal et al. (2022, Various)<br>Rajagopal et al. (2020, Mental health)

Notes: G stands for genotype, E for environment, E^* for environments *other than* those of interest, and rGE for gene–environment correlation. A predetermined environment E is defined as an environment not causally influenced by one's genes G yet possibly correlated with other environmental characteristics E^* and potentially shaped by parental genes.

Figure G.1: Densities of children's, mothers' and fathers' EA PGI by treatment status.

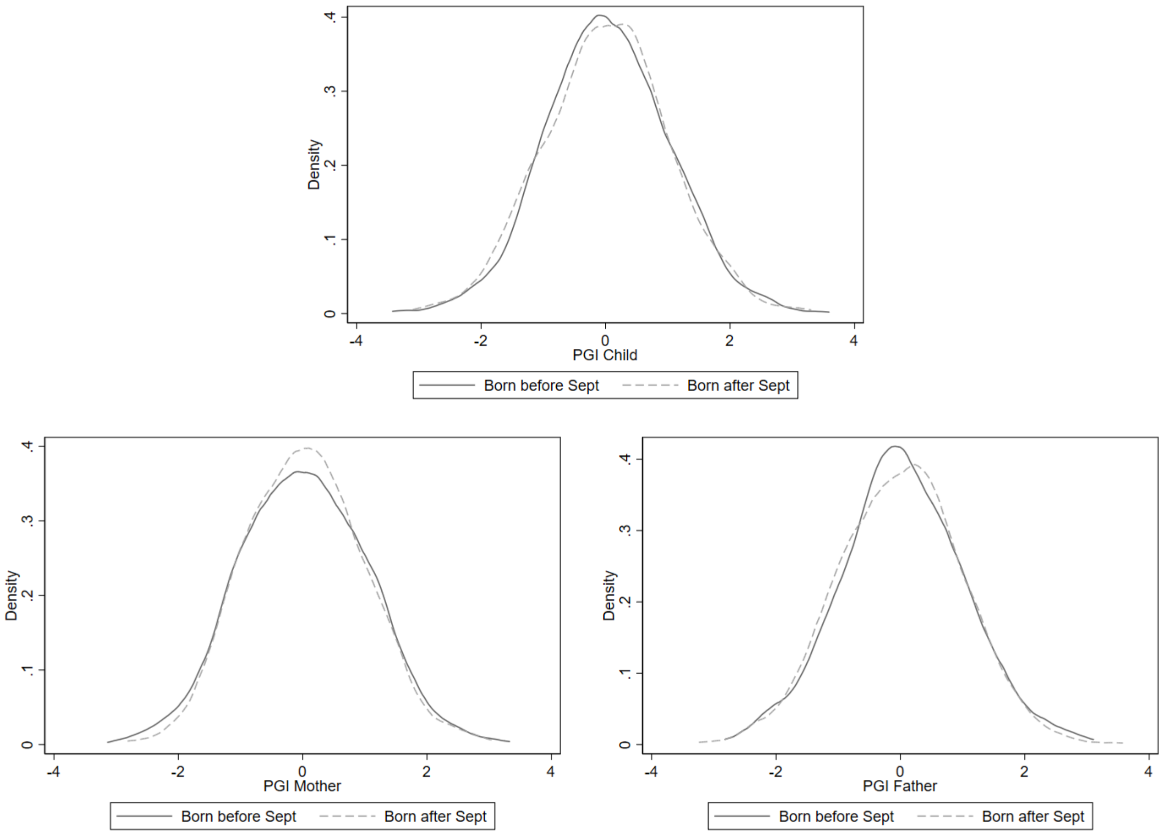

Figure G.2: The $G \times E$ coefficients for the Entry Assessment and Key Stage 1–4 test scores.

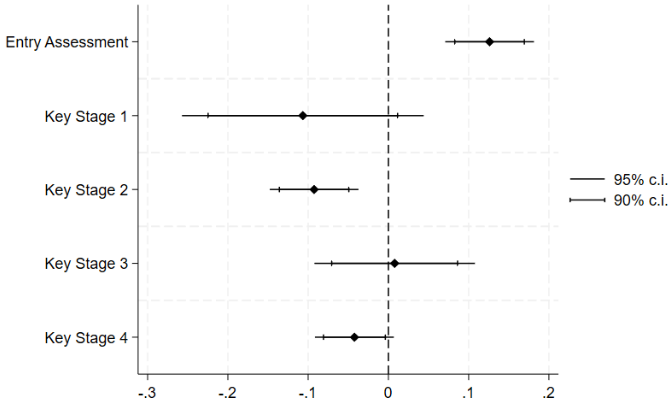

Table G.2: OLS estimates of the effect of the parental PGIs for EA on test scores at different ages.

	Entry Assessment	Key Stage 1	Key Stage 2	Key Stage 3	Key Stage 4
	Age 4-5	Age 6-7	Age 10-11	Age 13-14	Age 15-16
PGI Mother	0.093***	0.180***	0.196***	0.225***	0.176***
	(0.036)	(0.019)	(0.018)	(0.021)	(0.019)
PGI Father	0.084**	0.106***	0.198***	0.218***	0.186***
	(0.034)	(0.020)	(0.019)	(0.021)	(0.019)
R^2	0.073	0.081	0.084	0.102	0.088
Observations	1094	3436	3610	3073	3579

Notes: The test score and the EA PGI are standardized to have mean 0 and standard deviation 1 in the analysis sample. All regressions control for gender and the first ten principal components of the genetic data, as well as a dummy if the parental PGIs are missing. Robust standard errors in parentheses. * $p < 0.10$, ** $p < 0.05$, *** $p < 0.01$.

References (Appendix)

Ahlskog, R., Beauchamp, J., Okbay, A., Oskarsson, S., and Thom, K. (2024). Testing for treatment effect heterogeneity: Educational reform, genetic endowments, and family background. *Social Science Research Network*, 4758247.

Allegrini, A. G., Karhunen, V., Coleman, J. R., Selzam, S., Rimfeld, K., Von Stumm, S., Pingault, J.-B., and Plomin, R. (2020). Multivariable G-E interplay in the prediction of educational achievement. *PLoS Genetics*, 16(11):e1009153.

Allegrini, A. G., Selzam, S., Rimfeld, K., von Stumm, S., Pingault, J. B., and Plomin, R. (2019). Genomic prediction of cognitive traits in childhood and adolescence. *Molecular Psychiatry*, 24(6):819–827.

Arold, B. W., Hufe, P., and Stoeckli, M. (2022). Genetic endowments, educational outcomes and the mediating influence of school investments. *IZA Discussion Papers*, 15430.

Baker, S., Biroli, P., van Kippersluis, H., and Von Hinke, S. (2024). Advantageous early-life environments cushion the genetic risk for ischemic heart disease. *Proceedings of the National Academy of Sciences*, 121(27):e2314056121.

Barcellos, S. H., Carvalho, L., and Turley, P. (2021). The effect of education on the relationship between genetics, early-life disadvantages, and later-life SES. *NBER Working Paper*, 28750.

Bates, T. C., Maher, B. S., Medland, S. E., McAloney, K., Wright, M. J., Hansell, N. K., Kendler, K. S., Martin, N. G., and Gillespie, N. A. (2018). The nature of nurture: Using a virtual-parent design to test parenting effects on children's educational attainment in genotyped families. *Twin Research and Human Genetics*, 21(2):73–83.

Becker, J., Burik, C. A., Goldman, G., (...), Benjamin, D. J., Turley, P., and Okbay, A. (2021). Resource profile and user guide of the Polygenic Index Repository. *Nature Human Behaviour*, 5(1):1744–1758.

Benjamin, D. J., Cesarini, D., Chabris, C. F., (...), Grankvist, A., Hultman, C. M., and Lichtenstein, P. (2012). The promises and pitfalls of genoeconomics. *Annual Review of Economics*, 4(1):627–662.

Black, S. E., Devereux, P. J., and Salvanes, K. G. (2011). Too young to leave the nest? The effects of school starting age. *Review of Economics and Statistics*, 93(2):455–467.

Boardman, J. D., Daw, J., and Freese, J. (2013). Defining the environment in gene-environment research: Lessons from social epidemiology. *American Journal of Public Health*, 103(S1):S64–S72.

Bolyard, A. and Savelyev, P. A. (2024). Understanding the educational attainment polygenic index and its interactions with ses in determining health in young adulthood. Technical report, IZA Discussion Papers.

Briley, D. A., Harden, K. P., Bates, T. C., and Tucker-Drob, E. M. (2015). Nonparametric estimates of Gene×Environment interaction using local Structural Equation Modeling. *Behavior Genetics*, 45(5):581–596.

Chabris, C. F., Lee, J. J., Benjamin, D. J., Beauchamp, J. P., Glaeser, E. L., Borst, G., Pinker, S., and Laibson, D. I. (2013). Why it is hard to find genes associated with social science traits: Theoretical and empirical considerations. *American Journal of Public Health*, 103(S1):S152–66.

Chabris, C. F., Lee, J. J., Cesarini, D., Benjamin, D. J., and Laibson, D. I. (2015). The fourth law of behavior genetics. *Current Directions in Psychological Science*, 24(4):304–312.

Cheesman, R., Borgen, N. T., Lyngstad, T. H., Eilertsen, E. M., Ayorech, Z., Torvik, F. A., Andreassen, O. A., Zachrisson, H. D., and Ystrom, E. (2022). A population-wide gene-environment interaction study on how genes, schools, and residential areas shape achievement.

npj Science of Learning, 7(1):29.

Cheesman, R., Hunjan, A., Coleman, J. R., Ahmadzadeh, Y., Plomin, R., McAdams, T. A., Eley, T. C., and Breen, G. (2020). Comparison of adopted and nonadopted individuals reveals gene–environment interplay for education in the UK Biobank. *Psychological Science*, 31(5):582–591.

Choi, S. W., García-González, J., Ruan, Y., Wu, H. M., Porras, C., Johnson, J., Bipolar Disorder Working group of the Psychiatric Genomics Consortium, Hoggart, C. J., and O'Reilly, P. F. (2023). PRSet: Pathway-based polygenic risk score analyses and software. *PLoS Genetics*, 19(2):e1010624.

Conley, D., Domingue, B. W., Cesarini, D., Dawes, C., Rietveld, C. A., and Boardman, J. D. (2015). Is the effect of parental education on offspring biased or moderated by genotype? *Sociological Science*, 2(1):82.

Conley, D., Laidley, T. M., Boardman, J. D., and Domingue, B. W. (2016). Changing polygenic penetrance on phenotypes in the 20th century among adults in the us population. *Scientific Reports*, 6(1):30348.

Conley, D., Sotoudeh, R., and Laidley, T. (2019). Birth weight and development: bias or heterogeneity by polygenic risk factors? *Population Research and Policy Review*, 38(6):811–839.

Crawford, C., Dearden, L., and Meghir, C. (2010). When you are born matters: The impact of date of birth on educational outcomes in England. *IFS Working Papers*, 10/06.

Dias Pereira, R. (2021). Genetic advantage and equality of opportunity in education: Two definitions and an empirical application. *bioRxiv*, 472565.

Dias Pereira, R., Biroli, P., Galama, T., von Hinke, S., van Kippersluis, H., Rietveld, C. A., and Thom, K. (2022). Gene–environment interplay in the social sciences. In *Oxford Research Encyclopedia of Economics and Finance*. Oxford University Press.

Domingue, B. W., Belsky, D. W., Conley, D. C., Harris, K. M., and Boardman, J. D. (2015). Polygenic influence on educational attainment: New evidence from the National Longitudinal Study of Adolescent to Adult Health. *AERA Open*, 1(3):1–13.

Domingue, B. W., Kanopka, K., Mallard, T. T., Trejo, S., and Tucker-Drob, E. M. (2022). Modeling interaction and dispersion effects in the analysis of gene-by-environment interaction. *Behavior Genetics*, 52(1):56–64.

Domingue, B. W., Trejo, S., Armstrong-Carter, E., and Tucker-Drob, E. M. (2020). Interactions between polygenic scores and environments: Methodological and conceptual challenges. *Sociological Science*, 7(1):465–486.

Dudbridge, F. (2013). Power and predictive accuracy of polygenic risk scores. *PLoS Genetics*, 9(3):e1003348.

Duncan, L. E. and Keller, M. C. (2011). A critical review of the first 10 years of candidate gene-by-environment interaction research in psychiatry. *American Journal of Psychiatry*, 168(10):1041–1049.

Feigenberg, B., Ost, B., and Qureshi, J. A. (2023). Omitted variable bias in interacted models: A cautionary tale. *Review of Economics and Statistics*, in press.

Fletcher, J. (2023). Decoupling genetics from attainments: The role of social environments. *Economics & Human Biology*, 50(1):101259.

Fukushima, N., Von Hinke, S., and Sørensen, E. N. (2024). The long-term human capital and health impacts of a pollution reduction programme. *arXiv*, 2409.11839.

Hamer, D. H. (2000). Beware the chopsticks gene. *Molecular Psychiatry*, 5(1):11–13.

Hewitt, J. K. (2012). Editorial policy on candidate gene association and candidate gene-by-environment interaction studies of complex traits. *Behavior Genetics*, 42(1):1–2.

Houmark, M., Ronda, V., Agerbo, E., Mortensen, P., and Rosholm, M. (2022). Genetic and socioeconomic achievement gaps in elementary school. *IZA Discussion Paper*, 15418.

Howe, L. J., Nivard, M. G., Morris, T. T., (...) Brumpton, B., Hemani, G., and Davies, N. M. (2022). Within-sibship genome-wide association analyses decrease bias in estimates of direct genetic effects. *Nature Genetics*, 54(5):581–592.

International Human Genome Sequencing Consortium (2004). Finishing the euchromatic sequence of the human genome. *Nature*, 431(7011):931–45.

Isungset, M. A., Conley, D., Zachrisson, H. D., Ystrom, E., Havdahl, A., Njølstad, P. R., and Lyngstad, T. H. (2022). Social and genetic associations with educational performance in a scandinavian welfare state. *Proceedings of the National Academy of Sciences*, 119(25):e2201869119.

Johnson, R., Sotoudeh, R., and Conley, D. (2022). Polygenic scores for plasticity: A new tool for studying gene–environment interplay. *Demography*, 59(3):1045–1070.

Karlsson Linnér, R., Marioni, R. E., Rietveld, C. A., (...), Deary, I. J., Koellinger, P. D., and Benjamin, D. J. (2017). An epigenome-wide association study meta-analysis of educational attainment. *Molecular Psychiatry*, 22(12):1680–1690.

Keller, M. C. (2014). Gene×Environment interaction studies have not properly controlled for potential confounders: The problem and the (simple) solution. *Biological Psychiatry*, 75(1):18–24.

Kong, A., Benonisdottir, S., and Young, A. I. (2020). Family analysis with Mendelian imputations. *bioRxiv*, 185181.

Lee, J. J., Wedow, R., Okbay, A., (...), Visscher, P. M., Benjamin, D. J., and Cesarini, D. (2018). Gene discovery and polygenic prediction from a 1.1-million-person GWAS of educational attainment. *Nature Genetics*, 50(8):1112–1121.

Lin, M.-J. (2020). The social and genetic inheritance of educational attainment: Genes, parental education, and educational expansion. *Social Science Research*, 86(1):102387.

Liu, W., Li, M., Zhang, W., Zhou, G., Wu, X., Wang, J., Lu, Q., and Zhao, H. (2020). Leveraging functional annotation to identify genes associated with complex diseases. *PLoS Computational Biology*, 16(11):e1008315.

Løken, K. V., Mogstad, M., and Wiswall, M. (2012). What linear estimators miss: The effects of family income on child outcomes. *American Economic Journal: Applied Economics*, 4(2):1–35.

Marnetto, D., Pärna, K., Läll, K., Molinaro, L., Montinaro, F., Haller, T., Metspalu, M., Mägi, R., Fischer, K., and Pagani, L. (2020). Ancestry deconvolution and partial polygenic score can improve susceptibility predictions in recently admixed individuals. *Nature Communications*, 11(1):1628.

Mills, M., Barban, N., and Tropf, F. (2018). Gene-neighborhood interactions in educational attainment. *Population Association of America 2018 Annual Meeting*.

Mills, M., Barban, N., and Tropf, F. C. (2020). *An introduction to statistical genetic data analysis*. MIT Press.

Muslimova, D., Dias Pereira, R., Von Hinke, S., Van Kippersluis, H., Rietveld, C. A., and Meddens, S. F. W. (2023). Rank concordance of polygenic indices. *Nature Human Behaviour*, 7(5):802–811.

Muslimova, D., Van Kippersluis, H., Rietveld, C. A., Von Hinke, S., and Meddens, S. F. W. (2024). Gene-environment complementarity in educational attainment. *Journal of Labor Economics*, in press.

Nagpal, S., Tandon, R., and Gibson, G. (2022). Canalization of the polygenic risk for common diseases and traits in the UK Biobank cohort. *Molecular Biology and Evolution*, 39(4):msac053.

Okbay, A., Beauchamp, J. P., Fontana, M. A., (...), Koellinger, P. D., Cesarini, D., and Benjamin, D. J. (2016). Genome-wide association study identifies 74 loci associated with educational attainment. *Nature*, 533(7604):539–542.

Okbay, A., Wu, Y., Wang, N., (...), Beauchamp, J. P., Benjamin, D. J., and Young, A. I. (2022). Polygenic prediction of educational attainment within and between families from genome-wide association analyses in 3 million individuals. *Nature Genetics*, 54(4):437–449.

Papageorge, N. W. and Thom, K. (2020). Genes, education, and labor market outcomes: Evidence from the Health and Retirement Study. *Journal of the European Economic Association*, 18(3):1351–1399.

Pettersson, O. (2023). Genetic influences on educational attainment through the lens of the evolving swedish welfare state: A cross-level gene-environment interaction study based on polygenic indices and longitudinal register data. *bioRxiv*, 565287.

Price, A. L., Patterson, N. J., Plenge, R. M., Weinblatt, M. E., Shadick, N. A., and Reich, D. (2006). Principal components analysis corrects for stratification in genome-wide association studies. *Nature Genetics*, 38(8):904–909.

Quick, C., Anugu, P., Musani, S., (...), Sidore, C., Boehnke, M., and Fuchsberger, C. (2020). Sequencing and imputation in GWAS: Cost-effective strategies to increase power and genomic coverage across diverse populations. *Genetic Epidemiology*, 44(6):537–549.

Rajagopal, V. M., Trabjerg, B. B., Grove, J., (...), Agerbo, E., Borglum, A. D., and Demontis, D. (2020). Polygenic prediction of school performance in children with and without psychiatric disorders. *bioRxiv*, 203661.

Reich, D. E., Cargill, M., Bolk, S., Ireland, J., Sabeti, P. C., Richter, D. J., Lavery, T., Kouyoumjian, R., Farhadian, S. F., Ward, R., et al. (2001). Linkage disequilibrium in the human genome. *Nature*, 411(6834):199–204.

Rietveld, C. A., Esko, T., Davies, G. E., (...), Benjamin, D. J., Cesarini, D., and Koellinger, P. D. (2014). Common genetic variants associated with cognitive performance identified using the proxy-phenotype method. *Proceedings of the National Academy of Sciences*, 111(38):13790–13794.

Rietveld, C. A., Medland, S. E., Derringer, J., (...), Benjamin, D. J., Cesarini, D., and Koellinger, P. D. (2013). GWAS of 126,559 individuals identifies genetic variants associated with educational attainment. *Science*, 340(6139):1467–1471.

Rimfeld, K., Krapohl, E., Trzaskowski, M., Coleman, J. R. I., Selzam, S., Dale, P. S., Esko, T., Metspalu, A., and Plomin, R. (2018). Genetic influence on social outcomes during and after the Soviet era in Estonia. *Nature Human Behaviour*, 2(4):269–275.

Ronda, V., Agerbo, E., Bleses, D., Mortensen, P. B., Børglum, A., Mors, O., Rosholm, M., Hougaard, D. M., Nordentoft, M., and Werge, T. (2022). Family disadvantage, gender, and the returns to genetic human capital. *Scandinavian Journal of Economics*, 124(2):550–578.

Schmitz, L. L. and Conley, D. C. (2017). The effect of Vietnam-era conscription and genetic potential for educational attainment on schooling outcomes. *Economics of Education Review*, 61(1):85–97.

Selzam, S., Krapohl, E., Von Stumm, S., O'Reilly, P. F., Rimfeld, K., Kovas, Y., Dale, P., Lee, J., and Plomin, R. (2017). Predicting educational achievement from DNA. *Molecular Psychiatry*, 22(2):267–272.

Shaver, J. M. (2019). Interpreting interactions in linear fixed-effect regression models: When fixed-effect estimates are no longer within-effects. *Strategy Science*, 4(1):25–40.

So, H.-C. and Sham, P. C. (2017). Improving polygenic risk prediction from summary statistics by an empirical bayes approach. *Scientific Reports*, 7(1):41262.

Tan, T., Jayashankar, H., Guan, J., (...), Okbay, A., Benjamin, D. J., and Young, A. S. (2024).

Family-gwas reveals effects of environment and mating on genetic associations. *medRxiv*, 24314703.

The 1000 Genomes Project Consortium (2015). A global reference for human genetic variation. *Nature*, 526(7571):68–74.

Torvik, F. A., Eilertsen, E. M., Hannigan, L. J., (...), Njølstad, P. R., Havdahl, A., and Ystrom, E. (2022). Modeling assortative mating and genetic similarities between partners, siblings, and in-laws. *Nature Communications*, 13(1):1108.

Trejo, S. and Domingue, B. W. (2018). Genetic nature or genetic nurture? Introducing social genetic parameters to quantify bias in polygenic score analyses. *Biodemography and Social Biology*, 64(3-4):187–215.

Uchikoshi, F. and Conley, D. (2021). Gene-environment interactions and school tracking during secondary education: Evidence from the US. *Research in Social Stratification and Mobility*, 76(1):100628.

Ujma, P. P., Eszlári, N., Millinghoffer, A., Bruncsics, B., Petschner, P., Antal, P., Deakin, B., Bagdy, G., and Juhász, G. (2022). Genetic effects on educational attainment in hungary. *Brain and Behavior*, 12(1):e2430.

Van den Berg, G. J., von Hinke, S., and Vitt, N. (2023a). Early life exposure to measles and later-life outcomes: Evidence from the introduction of a vaccine. *arXiv*, 10558.

Van den Berg, G. J., von Hinke, S., and Wang, R. A. H. (2023b). Prenatal sugar consumption and late-life human capital and health: Analyses based on postwar rationing and polygenic scores. *arXiv*, 9982.

Van Kippersluis, H., Biroli, P., Galama, T., Von Hinke, S., Meddens, S. F. W., Muslimova, D., Dias Pereira, R., Slob, E. A. W., de Vlaming, R., and Rietveld, C. A. (2023). Overcoming attenuation bias in regressions using polygenic indices. *Nature Communications*, 14(1):4473.

Vilhjalmsson, B. J., Yang, J., Finucane, H. K., (...), Kraft, P., Patterson, N., and Price, A. L. (2015). Modeling linkage disequilibrium increases accuracy of polygenic risk scores. *American Journal of Human Genetics*, 97(4):576–592.

Visscher, P. M., Wray, N. R., Zhang, Q., Sklar, P., McCarthy, M. I., Brown, M. A., and Yang, J. (2017). 10 years of GWAS discovery: Biology, function, and translation. *American Journal of Human Genetics*, 101(1):5–22.

Von Hinke, S. and Sørensen, E. N. (2023). The long-term effects of early-life pollution exposure: Evidence from the London smog. *Journal of Health Economics*, 92(1):102827.

Von Stumm, S., Smith-Woolley, E., Ayorech, Z., McMillan, A., Rimfeld, K., Dale, P. S., and Plomin, R. (2020). Predicting educational achievement from genomic measures and socioeconomic status. *Developmental Science*, 23(3):e12925.

Ware, E. B., Schmitz, L. L., Faul, J. D., Gard, A., Mitchell, C., Smith, J. A., Zhao, W., Weir, D., and Kardia, S. L. R. (2017). Heterogeneity in polygenic scores for common human traits. *bioRxiv*, 106062.

Wickrama, K. A., O'Neal, C. W., Lee, T. K., and Lee, S. (2021). Early life course processes leading to educational and economic attainment in young adulthood: Contributions of early socioeconomic adversity and education polygenic score. *PLoS ONE*, 16(10):e0256967.

Yang, J., Lee, S. H., Goddard, M. E., and Visscher, P. M. (2011). GCTA: A tool for genome-wide complex trait analysis. *American Journal of Human Genetics*, 88(1):76–82.

Yengo, L., Vedantam, S., Marouli, E., (...), Wood, A. R., Visscher, P. M., and Hirschhorn, J. N. (2022). A saturated map of common genetic variants associated with human height. *Nature*, 610(7933):704–712.
